# Genome-scale metabolic network guided engineering of *Streptomyces tsukubaensis* for FK506 production improvement

**DOI:** 10.1186/1475-2859-12-52

**Published:** 2013-05-24

**Authors:** Di Huang, Shanshan Li, Menglei Xia, Jianping Wen, Xiaoqiang Jia

**Affiliations:** 1 Department of Biochemical Engineering, School of Chemical Engineering and Technology, Tianjin University, Tianjin 300072, PR China; 2 Ministry of Education, Key Laboratory of Systems Bioengineering, Tianjin 300072, PR China; 3 TEDA School of Biological Sciences and Biotechnology, Nankai University, TEDA, Tianjin 300457, PR China

**Keywords:** *Streptomyces tsukubaensis*, FK506, Genome-scale metabolic model, Target prediction, Metabolic engineering, Combinatorial modification

## Abstract

**Background:**

FK506 is an important immunosuppressant, which can be produced by *Streptomyces tsukubaensis*. However, the production capacity of the strain is very low. Hereby, a computational guided engineering approach was proposed in order to improve the intracellular precursor and cofactor availability of FK506 in *S. tsukubaensis*.

**Results:**

First, a genome-scale metabolic model of *S. tsukubaensis* was constructed based on its annotated genome and biochemical information. Subsequently, several potential genetic targets (knockout or overexpression) that guaranteed an improved yield of FK506 were identified by the recently developed methodology. To validate the model predictions, each target gene was manipulated in the parent strain D852, respectively. All the engineered strains showed a higher FK506 production, compared with D852. Furthermore, the combined effect of the genetic modifications was evaluated. Results showed that the strain HT-ΔGDH-DAZ with *gdhA*-deletion and *dahp*-, *accA2*-, *zwf2*-overexpression enhanced FK506 concentration up to 398.9 mg/L, compared with 143.5 mg/L of the parent strain D852. Finally, fed-batch fermentations of HT-ΔGDH-DAZ were carried out, which led to the FK506 production of 435.9 mg/L, 1.47-fold higher than the parent strain D852 (158.7 mg/L).

**Conclusions:**

Results confirmed that the promising targets led to an increase in FK506 titer. The present work is the first attempt to engineer the primary precursor pathways to improve FK506 production in *S. tsukubaensis* with genome-scale metabolic network guided metabolic engineering. The relationship between model prediction and experimental results demonstrates the rationality and validity of this approach for target identification. This strategy can also be applied to the improvement of other important secondary metabolites.

## Background

**Figure 1:**
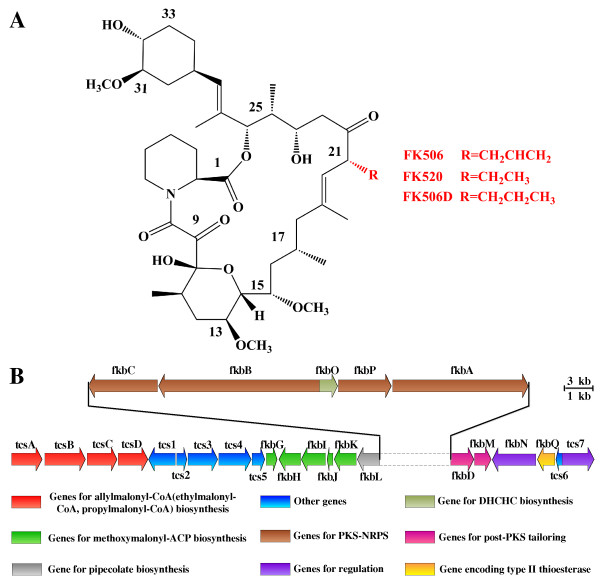
**Structures and biosynthetic gene cluster of FK506 and byproducts (FK520, FK506D) in *S. tsukubaensis*.** (**A**) Structures of FK506 and byproducts FK520, FK506D. The allyl side chain at C-21 of FK506 is replaced by an ethyl group in FK520 and a propyl group in FK506D. (**B**) Schematic representation of FK506 and analogues (FK520, FK506D) biosynthetic gene cluster in *S. tsukubaensis*.

FK506 (tacrolimus), which is produced by *Streptomyces tsukubaensis*, is a 23-membered polyketide macrolide (Figure 1A). It has been used as an immunosuppressant after the transplantation of allogeneic kidney, liver and bone marrow as well as for the treatment of inflammatory skin diseases and eczema [1-3]. FK506 structural cluster contains polyketide synthase (PKS) and non-ribosomal peptide synthetase (NRPS) (Figure 1B), which belongs to an amide bond-containing macrolide family with ascomycin and rapamycin [4-7]. PKS is responsible for assembling specific carboxylic acid-derived extender units into polyketide chains and catalyzing subsequent reductive reactions. NRPS incorporates various amino acids or amino acid derivatives into non-ribosomally synthesized peptides.

Recently, tremendous efforts based on the random mutagenesis and selection approaches have been dedicated to the development of efficient FK506 synthesis. As a major bottleneck, the FK506 yield in fermentation process is relatively low, which may be due to the limited level of intracellular precursors. Thus, it is essential to engineer the strain to improve the requirement of precursors for product formation. Indeed, metabolic engineering efforts aimed at improving intracellular precursors have regulated the key pathways of natural products such as polyketide and polypetide. For example, it was reported that overexpression of propionyl-CoA carboxylase, methylmalonyl-CoA mutase, and malonyl/methylmalonyl-CoA ligase led to the accumulation of methylmalonyl-CoA, a key precursor of FK506 [8]. Besides, the precursor pathway enhancement has been performed by strengthening the supply of unusual polyketide extender units [9].

Genetic manipulations may improve the precursor synthesis and product titer. Nevertheless, the approach is always uncertain and blind for target genes validation. Additionally, as cells have highly interconnected metabolic pathways, overexpression or knockout of the specific pathway may generate an effect on other pathways and subsequently on the cell growth. Importantly, since the scope of engineering is local, there may exist some limitations for strain improvement. Therefore, it is challenging to evaluate the cellular behaviors comprehensively and identify the accurate target genes for efficient strain improvement from the systems-level.

Developed by the techniques such as flux balance analysis (FBA) and minimization of metabolic adjustment (MOMA), systems biology plays an important role in strain metabolic engineering by changing metabolic flux distribution within a microorganism based on systematic strategies. As more microorganisms have been sequenced, the reconstruction of a genome-scale metabolic model (GSMM) becomes necessary in gaining comprehensive insight into microbial physiology [10]. Stoichiometric models-based GSMM can be employed to interpret cellular metabolic response to genetic perturbation and unravel the underlying reasons of undesired phenotypes. This approach saves time, labor, and research expenditure by decreasing the amounts of wet-experiments. Currently, GSMMs have been used to identify metabolic engineering targets for many important industrial products such as biofuels, vitamins, amino acids and secondary metabolites [11-14].

In the past decades, various computational strain design algorithms, such as MOMA, ROOM, OptKnock, OptReg, OptORF, RobustKnock, OptStrain and OptForce have been developed to efficiently predict target genes for improved product yield [15-22]. Among the above approaches, intracellular flux distribution can be calculated by maximization of cell biomass or minimization of metabolic adjustments. The calculated results which largely reveal the physiological state of the wild-type strain can be used to inactivate the target pathways for improved production. In addition to the knockout prediction, the overexpression prediction algorithm has also been recently developed to direct application of product overproduction [23,24].

In this study, a GSMM of *S. tsukubaensis* was reconstructed to simulate the intracellular flux distribution. Guided by FBA and MOMA prediction, several genetic targets that were outside of the secondary metabolic pathways were identified. These targets were then screened and validated experimentally through metabolic engineering as well as subsequent batch fermentations. Moreover, the gene knockout and overexpression combinatorial strategy was implemented to enhance the FK506 production up to 435.9 mg/L of final titer, an approximately 2-fold increase relative to the parent strain.

## Results

### GSMM reconstruction for *S. tsukubaensis*

The first genome sequence draft of the FK506 producer, *S. tsukubaensis* NRRL 18488 has been reported recently [25]. In the present work, GSMM was reconstructed using an automated procedure as well as manual refinement (see Materials and methods section). By integrating the genome annotation results, a draft model including 865 reactions and 621 metabolites was obtained. Here, the biomass composition was obtained by integrating the corresponding results related to other *Streptomyces* from experiments and references. The energetic coefficients for both cell growth and maintenance were also evaluated based on the previous report [26].

**Figure 2:**
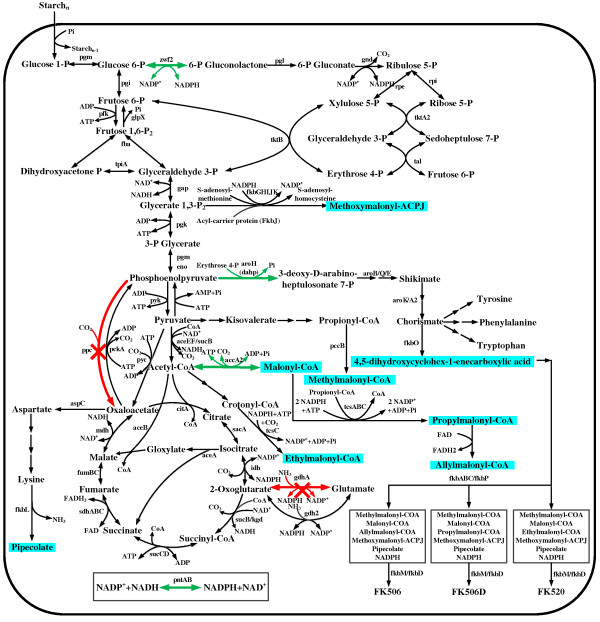
**Schematic representation of metabolic pathways for *S. tsukubaensis*.** The predicted targets for improved production are shown in green (amplification) and red (knockout). The shaded boxes represent precursors of FK506 biosynthesis.

Major metabolic pathways for *S. tsukubaensis*, including the glycolytic pathway, pentose phosphate pathway (PPP), tricarboxylic acid (TCA) cycle, and FK506 biosynthesis, are illustrated in Figure 2. Additionally, the details of the biosynthetic pathways for FK506 precursors are also presented (Additional file 1: Text S1, Additional file 2: Text S2). It can provide some advantages about how carbon is diverted to these pathways, thus resulting in an efficient FK506 production in *S. tsukubaensis*.

### Potential target genes identification based on *in silico* simulations

#### Gene knockout simulation

**Figure 3:**
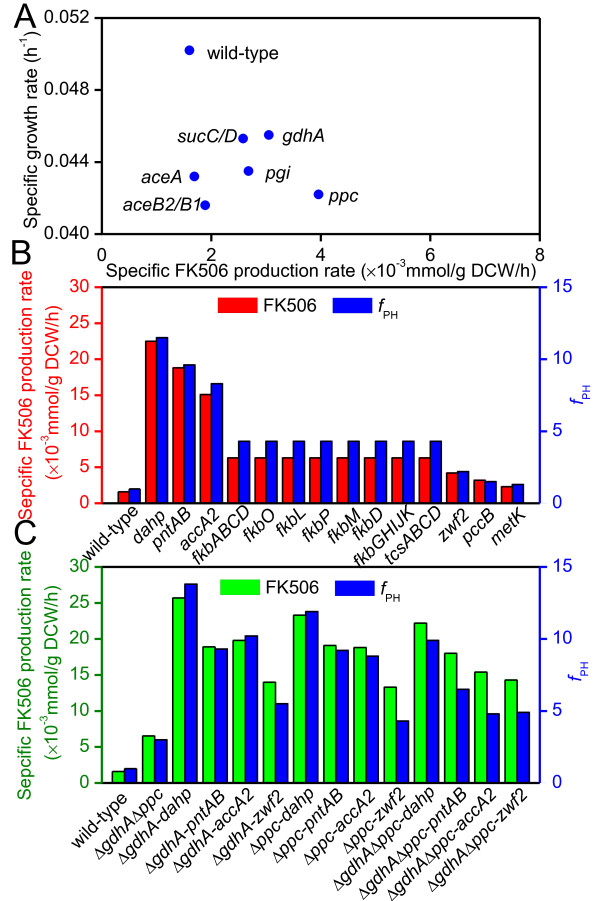
**Single gene knockout, overexpression and knockout-overexpression combinations target identification in *S. tsukubaensis*.** (**A**) The effect of single gene knockout on the specific FK506 production rate and the specific growth rate. (**B**) The effect of single gene overexpression on the specific FK506 production and *f*_PH_. (**C**) The effect of knockout-overexpression combinations on the specific FK506 production and *f*_PH_. The enzymes encoded by these genes are as follows: *aceA*, isocitrate lyase; *aceB2/B1*, malate synthase; *sucC/D*, succinyl-CoA synthase; *gdhA*, glutamate dehydrogenase; *pgi*, glucose-6-phosphate isomerase; *ppc*, phosphoenolpyruvate carboxylase; *dahp*, 3-deoxy-D-arabino-heptulosonate-7-phosphate synthase; *pntAB*, pyridine nucleotide transhydrogenase; *accA2*, acetyl-CoA carboxylase; *fkbA*, *fkbB*, *fkbC*, polyketide synthase; *fkbO*, chorismatase, 4,5-dihydroxycyclohex-1-enecarboxylic acid (DHCHC) synthesis; *fkbL*, lysine cyclodeaminase; *fkbP*, non-ribosomal peptide synthetase; *fkbM*, 31-O-methyltrasferase; *fkbD*, C9 hydroxylase; *fkbG*, *fkbH*, *fkbI*, *fkbJ*, *fkbK*, genes for methoxymalonyl-ACP synthesis; *tcsA*, *tcsB*, *tcsC*, *tcsD*, genes for allymalonyl-CoA synthesis; *zwf2*, glucose-6-phosphate dehydrogenase; *pccB*, propionyl-CoA carboxylase; *metK*, S-adenosylmethionine synthetase.

First, FK506 was assumed as the extracellular metabolite here for convenient calculation, although it belonged to the intracellular product. Then the experimental specific production rate of FK506 was determined to be 1.61 ± 0.10 μmol/g DCW/h and was set as the lower bound for FK506 transport flux. In this work, glucose was not implemented as the principal carbon source during the *in silico* calculation due to the fact that *S. tsukubaensis* could not produce the high efficient of FK506 in glucose-based medium. Instead, our previous experiences proved that starch was beneficial to FK506 production. Since the carbon source (starch) harbored long-chain whose molecular formula was uncertain, the specific uptake rate of carbon source was defined as the production rate of glucose-1-phosphate derived from starch which was 3.472 mmol/g DCW/h. The upper bound of the specific uptake rates of twenty L-amino acids was set to 0.050 mmol/g DCW/h based on our experimental data (the specific consumption rates of all amino acids were between 0 and 0.050 mmol/g DCW/h during initial exponential growth phase and late exponential growth phase). Using these experimental constrains, the specific growth rate was predicted to be 0.0495 h^-1^, consistent with the experimental data (0.0502 h^-1^). For a higher productivity of FK506, potential gene knockout targets were identified by MOMA algorithm (Figure 3A). Initially, several single gene knockouts were identified. Here, we discussed the cases that specific growth rate was more than 0.04 h^-1^. Among these promising target genes, the gene *aceB2*/*B1* or *aceA*, was predicted to inactivate the glyoxylate shunt and thus avoided the consumption of acetyl-CoA. The flux from acetyl-CoA to biomass was partially diverted towards the precursors of FK506 biosynthesis. As shown in Figure 2, acetyl-CoA can be transformed to malonyl-CoA and subsequently allylmalonyl-CoA, both of which are the direct precursors of FK506. Deletion of *sucC*/*D* could result in a reduction of the TCA cycle activity, thereby reducing the consumption of the acetyl-CoA pool and indirectly enhancing the precursors supply (malonyl-CoA and allylmalonyl-CoA). Knockout of *pgi* gene could divert the carbon flux from glycolytic pathway to PPP, which would improve the NADPH availability, since the FK506 biosynthesis need many NADPH (Figure 2, Additional file 2: Text S2). In the following analysis, *sucC*/*D* was excluded from knockout candidates. Although blocking the TCA cycle at succinyl-CoA synthase can indirectly affect the carbon flows towards the methylmalonyl-CoA pool, it also results in two fewer moles of ATP. Additionally, *aceA* or *aceB2*/*B1* or *pgi* was not selected because the level of FK506 enhancement and cell growth were not the optimal. Therefore, after removing these targets, *gdhA* and *ppc* were selected as candidates for the following experimental validation as they both allowed an increased specific FK506 production rate with less reduction in the specific growth rate. The *gdhA* gene directly impact the supply of FK506 redox cofactor since its deletion could increase the availability of NADPH [27] for other NADPH coupled enzymes such as ketoreductase and enoylreductase. This strain optimization strategy not only increased FK506 production rate by approximately 80%, up to 3.05 μmol/g DCW/h, compared with the wild type (1.61 μmol/g DCW/h), but also exerted a small effect on cell growth (the maximum growth rate of 0.0455 h^-1^). Another screened target was *ppc* gene encoding the phosphoenolpyruvate carboxylase (PPC). Inactivation of *ppc* gene would directly enhance the phosphoenolpyruvate (PEP) pool, thus providing more precursors to synthesize 4,5-dihydroxycyclohex-1-enecarboxylic acid (DHCHC, starter of FK506 formation, Figure 2). Noted that although initial metabolic flux distribution in FBA simulation were undetermined, the *in silico* prediction for targets was unique. These simulated results indicated that secondary metabolite production could be improved by balancing the precursors and lowering the growth rate [11]. This insight into the *S. tsukubaensis* metabolism offered a mechanism of how this strain was redesigned towards efficient FK506 synthesis.

#### Gene overexpression simulation

Using the *f*_PH_, overexpression targets for improving FK506 production were identified [24]. Figure 3B shows the *f*_PH_ of several important reactions leading to FK506 overproduction. Among these targets, *dahp* had the highest ratio (*f*_PH_ =11.5), indicating that increasing carbon flux through this pathway may facilitate FK506 production. In fact, the primary shikimate pathway played a key role in the supply of precursor chorismate, which was used as the amino donor in DHCHC biosynthesis [28]. The second target suggested that an increase in NADPH biosynthesis may accommodate polyketide chain enoyl- and keto- reduction. Therefore, it was necessary to overexpress *pntAB* for achieving optimal NADPH levels to improve FK506 biosynthesis. Similarly, *accA2* also had a large *f*_PH_, indicating the pathway as a potential overexpression target. Overexpressing this gene would increase the precursor (malonyl-CoA) level of FK506.

In addition, Figure 3B also shows that overexpressing the secondary pathway genes such as *fkbABCD*, *fkbL*, *fkbM*, *fkbO*, *fkbP*, *fkbGHIJK*, *tcsABCD*, can improve levels of FK506 flux, signifying a need for increased metabolic flux through the secondary pathway. These strategies can increase the available precursor pool. However, compared with the above targets, these targets showed a little increase in FK506 production rate since overexpressing the individual gene may lead to precursor shortage of the other pathways. Additionally, there existed some difficulty in multiple genes manipulation. Therefore, genes of the secondary metabolic pathway were not considered in this work. The next target was to enhance the PPP through overexpressing *zwf2* gene. In fact, *zwf2* also played a key role in the supply of NADPH for FK506 biosynthesis. In the *zwf2* overexpression strain, the increase in PPP flux indicated an enhanced drain of carbon to erythrose-4-phosphate (E4P), direct precursor of shikimate pathway. In addition, glucose-6-phosphate dehydrogenase (G6PDH) enhancement by overexpressing *zwf2* could result in an increase in intracellular PPP flux and precursor pool, thus improving the product titer [29,30]. Lastly, *pccB* and *metK* encoding propionyl-CoA carboxylase and S-adenosylmethionine synthetase, respectively, were also predicted to exert a beneficial effect on FK506 production. Here, they were not selected for the following genetic modifications, since the former was demonstrated to result in a limited improvement [8] while the latter was different from *fkbM* which remained unknown and required a more in-depth investigation in future. Further simulations, however, showed no other potential overexpression targets leading to higher FK506 production. Therefore, the genes *dahp*, *pntAB*, *accA2* and *zwf2* were identified as overexpression targets for the enhanced production of FK506.

#### Multiple genes knockout-overexpression combinations simulation

According to the result of single gene knockout or overexpression simulation, we also attempted to simulate the effect of multiple genes knockout-overexpression on the FK506 production and cell growth. Here, *gdhA* and *ppc* were selected as knockout targets, *dahp*, *pntAB*, *accA2* and *zwf2* as overexpression targets. Due to the limitation of the algorithm, multiple genes overexpression results could not be predicted. This was because that when genes A and B were amplified by 2-fold simultaneously, it was difficult to control them in the same fold increase in real experiment (overexpressing a specified gene based on a ten-copy plasmid did not mean ten-fold the transcription level of a one-copy plasmid, nor did it mean ten-fold the protein amount, nor did it mean ten-fold metabolic flux value through the reaction). In this study, we simulated *gdhA* and *ppc* double knockout, combination of *gdhA* (or *ppc*) and single overexpression. As shown in Figure 3C, the specific growth rate of *gdhA* and *ppc* double knockout was 0.037 h^-1^, and the specific FK506 production rate was 6.53 μmol/g DCW/h. When the single gene knockout combined with the single gene overexpression, all the mutants showed an increase in the specific FK506 production rate, compared with the cases of single gene manipulation (knockout or overexpression). Besides, the *in silico gdhA* mutants (HT-ΔGDH-DAHP, HT-ΔGDH-ACC, HT-ΔGDH-PNT, HT-ΔGDH-ZWF) displayed higher specific FK506 production rate and *f*_PH_ than *in silico ppc* mutants (HT-ΔPPC-DAHP, HT-ΔPPC-ACC, HT-ΔPPC-PNT, HT-ΔPPC-ZWF). Especially, *gdhA* inactivation and *dahp* overexpression (*in silico* HT-ΔPPC-DAHP) gave rise to the highest *f*_PH_. For double genes knockout combined with single gene overexpression, it could not further improve the specific FK506 production rate and *f*_PH_.

### Experimental validation of predicted targets

#### Impact of single gene disruption on cell growth and FK506 synthesis

Gene deletion was conducted to experimentally investigate the prediction of the above knockout simulations. The target genes *gdhA* and *ppc* were inactivated by the kanamycin and thiostrepton resistance cassette, respectively, without affecting the expression of the downstream genes.

**Figure 4:**
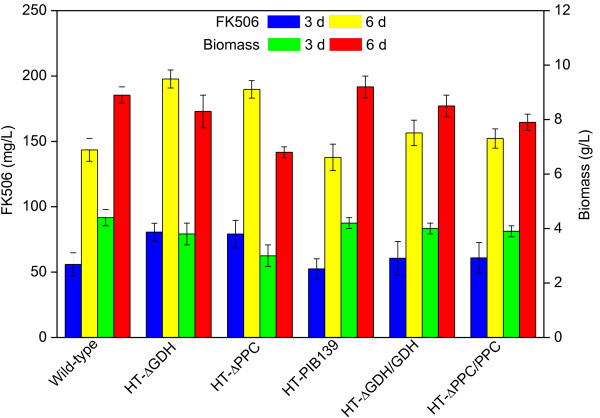
**The effect of single gene knockout and complementation on FK506 production and cell growth.** The data are the average values of at least three series of three parallel tests, and error bars represent standard deviations.

**Figure 5:**
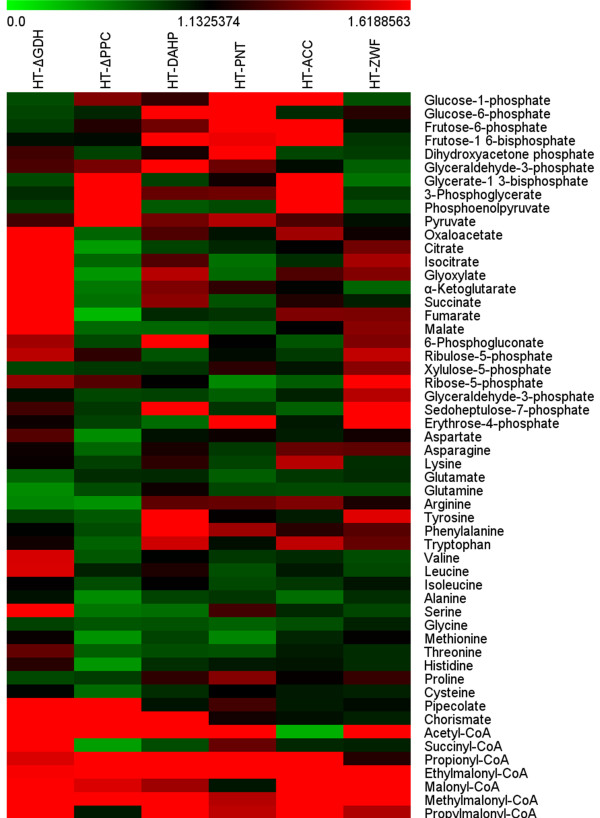
**Intracellular metabolite profiles at 72 h for engineered strains.** Heat map visualizes most intracellular metabolites from glycolysis, TCA, PPP and amino acid pathways of engineered strains under the same fermentation condition. The color code indicates an increased (red) or a decreased (green) availability as compared to the reference wild-type strain D852, as indicated by the color legend as aside the graph. Availability for each metabolite was calculated as ratio of the concentration of each engineered strain to that of the wild-type strain D852. The data are the average values of at least five series of five parallel tests.

The positive mutants were confirmed by PCR amplification and sequencing of the disrupted region. Results showed that there was no change in morphological phenotype between the *gdhA* or *ppc* single deletion mutant and the parent strain D852. However, the growth of HT-ΔGDH strain was slightly affected, with a lower biomass concentration (8.1 ± 0.6 g/L and 3.4 ± 0.1 g/L for 6 d and 3 d, respectively) as compared with the parent strain D852 (8.9 ± 0.3 g/L and 4.4 ± 0.3 g/L for 6 d and 3 d, respectively) (Figure 4). Additionally, just as expected, the NADPH-dependent glutamate dehydrogenase (GDH1) activities of HT-ΔGDH strain at 72 h and 144 h were assayed to 0.25 ± 0.05 U/mg and 0.13 ± 0.04 U/mg, respectively, while these were 55.85 ± 1.36 U/mg and 36.47 ± 1.09 U/mg of D852 (Table 1), indicating that the target enzyme was replaced by the resistance cassette. Nevertheless, it was noted that NADH-dependent glutamate dehydrogenase (GDH2) which also catalyzes α-ketoglutarate and ammonia to glutamate (Figure 2) increased from 60.75 ± 5.21 U/mg to 79.55 ± 1.66 U/mg at 72 h (Table 1). This observation might be ascribed to the fact that the inactivated GDH1 activity up-regulated the GDH2 activity for glutamate biosynthesis. The intracellular glutamate decreased, whereas the inactivated GDH1 enzyme resulted in intracellular accumulation of the substrate α-ketoglutarate (Figure 5). As an intermediate of TCA cycle, α-ketoglutarate could be converted to other organic acids such as succinate and fumarate. Therefore, the levels of these TCA intermediates were also improved as shown in Figure 5. During the batch cultivation, total sugar consumption rate was decreased by 10%, from 0.49 ± 0.03 g/L/h to 0.44 ± 0.02 g/L/h, while FK506 production was increased from 143.5 ± 8.7 mg/L to 197.7 ± 7.9 mg/L at 6 d, compared with parent strain D852 (Figure 4). Besides, the NADPH level in the mutant was enhanced as expected (Table 2), which revealed the important role of the *gdhA* gene in redox cofactor biosynthesis.

**Table 1:** **Specific activity of enzymes by parent strain*S. tsukubaensis*D852 and recombinants in batch cultures**

**Enzyme activities (U/mg protein)**	**Time (h)**	**D852**	**HT-ΔGDH**	**HT-ΔPPC**	**HT-DAHP**	**HT-PNT**	**HT-ACC**	**HT-ZWF**
GDH1	72	55.85 ± 1.36	0.25 ± 0.05	-	-	-	-	-
	144	36.47 ± 1.09	0.13 ± 0.04	-	-	-	-	-
GDH2	72	60.75 ± 5.21	79.55 ± 1.66	-	-	-	-	-
	144	50.32 ± 3.98	54.67 ± 4.42	-	-	-	-	-
PPC	72	0.85 ± 0.04	-	0.08 ± 0.05	-	-	-	-
	144	0.32 ± 0.11	-	0.10 ± 0.04	-	-	-	-
PK	72	0.86 ± 0.02	-	1.12 ± 0.03	-	-	-	-
	144	0.47 ± 0.03	-	0.60 ± 0.02	-	-	-	-
PC	72	2.25 ± 0.22	-	2.49 ± 0.16	-	-	-	-
	144	1.77 ± 0.13	-	1.89 ± 0.12	-	-	-	-
DAHP	72	0.15 ± 0.03	-	-	0.26 ± 0.02	-	-	-
	144	0.11 ± 0.03	-	-	0.19 ± 0.04	-	-	-
PNT	72	0.07 ± 0.02	-	-	-	0.98 ± 0.02	-	-
	144	0.19 ± 0.05	-	-	-	1.69 ± 0.09	-	-
ACC	72	Not detected	-	-	-	-	Not detected	-
	144	Not detected	-	-	-	-	Not detected	-
G6PDH	72	0.13 ± 0.03	-	-	-	-	-	0.29 ± 0.04
	144	0.08 ± 0.02	-	-	-	-	-	0.15 ± 0.05

Results are represented as mean ± SD of three independent observations.

**Table 2:** **Intracellular concentrations of NAD**^**+**^**, NADH, NADP**^**+**^**and NADPH during exponential growth in batch cultures**

**Strains**	**Intracellular concentration (μmol/g biomass)**				**NADH/ NAD**^**+**^**ratio**	**NADPH/ NADP**^**+**^**ratio**
	**NAD**^**+**^	**NADH**	**NADP**^**+**^	**NADPH**		
D852	2.33 ± 0.05	0.85 ± 0.03	0.72 ± 0.04	1.89 ± 0.05	0.36 ± 0.03	2.63 ± 0.22
HT-ΔGDH	1.31 ± 0.05	0.59 ± 0.10	0.65 ± 0.06	3.31 ± 0.09	0.45 ± 0.10	5.09 ± 0.67
HT-ΔPPC	2.67 ± 0.02	0.73 ± 0.06	0.95 ± 0.06	1.38 ± 0.07	0.27 ± 0.03	1.45 ± 0.18
HT-DAHP	2.58 ± 0.02	0.88 ± 0.05	0.68 ± 0.07	1.80 ± 0.02	0.34 ± 0.02	2.65 ± 0.33
HT-PNT	2.55 ± 0.09	0.59 ± 0.04	0.47 ± 0.03	2.19 ± 0.06	0.23 ± 0.03	4.66 ± 0.45
HT-ACC	2.47 ± 0.04	0.95 ± 0.02	0.67 ± 0.07	1.68 ± 0.04	0.38 ± 0.02	2.50 ± 0.37
HT-ZWF	2.35 ± 0.09	0.91 ± 0.04	0.34 ± 0.03	2.28 ± 0.06	0.39 ± 0.03	6.71 ± 0.84

Results are represented as mean ± SD of three independent observations.

As for the *ppc* mutant strain HT-ΔPPC, the PPC activity was almost reduced to zero, compared with the value of the parent strain D852 (0.85 ± 0.04 U/mg at 72 h) (Table 1), indicating that the enzyme was completely inactivated. In addition, another anaplerotic pathway enzyme pyruvate carboxylase (PC) was slightly increased for the *ppc* mutant strain; whereas, the activity of pyruvate kinase (PK), which catalyzes the reaction from PEP to pyruvate, was increased to 1.12 ± 0.03 U/mg at 72 h in HT-ΔPPC strain, compared with 0.86 ± 0.02 U/mg at 72 h in D852 (Table 1). These observations indicated that PEP precursor was partially channeled towards the pyruvate formation and TCA cycle was maintained through pyruvate anaplerotic pathway. According to the batch culture profile, the HT-ΔPPC strain exhibited a longer lag time (24 h compared to 12 h in parent strain), and was retarded during exponential phase growth, with 6.8 ± 0.2 g/L biomass at 6 d (Figure 4). Despite the suppressed cell growth, FK506 production were increased to 79.1 ± 10.5 mg/L and 189.8 ± 6.7 mg/L at 3 d and 6 d, almost 1.4- and 1.3-fold of the parent strain (55.9 ± 8.9 mg/L and 143.5 ± 8.7 mg/L), respectively. Furthermore, it was worth noting that intracellular PEP and pyruvate concentration in HT-ΔPPC mutant increased by approximately 5-fold and 1.8-fold, respectively, compared with D852 (Figure 5), indicating that carbon flux from PEP node was redistributed and converted to more precursors (chorismate) for FK506 production.

Besides, the specific FK506 production rate for HT-ΔGDH strain and HT-ΔPPC strain were 1.88 ± 0.05 and 1.92 ± 0.08 μmol/g DCW/h, respectively, higher than the parent strain D852 (1.61 ± 0.10 μmol/g DCW/h) (Additional file 3: Table S1), but lower than the predicted value (Figure 3A). Furthermore, to explore the relationship between the reduced intermediate and cell growth, we tested the growth of these mutants with addition of intermediate precursors (glutamate or glutamine for HT-ΔGDH strain, TCA cycle intermediates such as succinate, fumarate or malate for HT-ΔPPC strain). Results showed that the mutant strains restored cell growth (Additional file 4: Figures S1 and S2).

#### Complementation of the disruption mutants

It was assumed that increased FK506 production was ascribed to the accumulation of the precursors in the *gdhA* or *ppc* disruption mutant. To confirm this, complementation experiments were carried out in the mutant strains HT-ΔGDH and HT-ΔPPC by introducing *gdhA* and *ppc*, plus their native RBS sites using pIB139 plasmid, respectively. First, a control experiment was performed that the parent strain D852 was transferred by pIB139 plasmid. As expected, the control strain HT-PIB139 produced an unchanged level of FK506 production and biomass compared with D852, indicating that the plasmid did not influence the fermentation characteristics of the strain D852. Subsequently, the constructed plasmids pGDH and pPPC were introduced into HT-ΔGDH and HT-ΔPPC, respectively. As shown in Figure 4, FK506 in both HT-ΔGDH/GDH and HT-ΔPPC/PPC strains were reduced by 25% than the corresponding mutant (HT-ΔGDH and HT-ΔPPC) at 6 d, which was almost the same as those produced by the parent strain D852. Additionally, the cell growth was improved but still lower than the level of D852, which might be due to the metabolic burden to the host. The above results provided evidence that the targeted disruption of *gdhA* or *ppc* gene correlated with the increased production of FK506.

#### Impact of single gene overexpression on cell growth and FK506 synthesis

To validate the overexpression targets selected by *f*_PH_, the target genes were cloned into pIB139 carrying the ermE* promoter and subsequently introduced into the *S. tsukubaensis* D852 via conjugation from *Escherichia coli* ET12567/pUZ8002 (see details in Materials and methods). In this section, four positive targets (*dahp* and *accA2* from *Streptomyces roseosporus*, *pntAB* and *zwf2* from *Streptomyces coelicolor*) were heterologously overexpressed in *S. tsukubaensis* D852.

**Figure 6:**
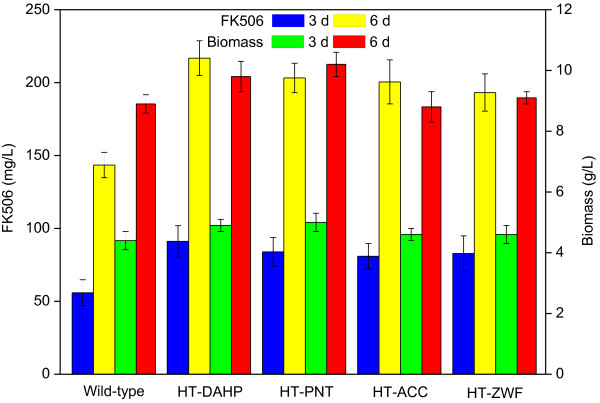
**The effect of single gene overexpression on FK506 production and cell growth.** The data are the average values of at least three series of three parallel tests, and error bars represent standard deviations.

First, target gene *dahp* encoding 3-deoxy-D-arabino-heptulosonate-7-phosphate (DAHP) synthase was overexpressed in D852. The DAHP synthase activity of the resulting strain HT-DAHP was 0.26 ± 0.02 U/mg and 0.19 ± 0.04 U/mg at 72 h and 144 h, respectively, approximately doubled those of wild-type D852 (0.15 ± 0.03 U/mg and 0.11 ± 0.03 U/mg at 72 h and 144 h, respectively) (Table 1), confirming that heterologous *dahp* gene functioned well. The cell growth was changed little for the engineered strain (9.8 ± 0.5 g/L at 6 d) (Figure 6). Importantly, overexpression of DAHP synthase enhanced FK506 production by approximately 50%, up to 216.8 ± 11.9 mg/L at 6 d, compared with the parent strain D852 (143.5 ± 8.7 mg/L). Accordingly, the specific FK506 production rate (2.56 ± 0.09 μmol/g DCW/h) for HT-DAHP was higher than that for the corresponding wild-type D852 (1.61 ± 0.10 μmol/g DCW/h) (Additional file 3: Table S3), indicating that *dahp* gene overexpression played a positive role in FK506 production.

Another predicted gene *pntAB* encoding membrane-bound transhydrogenase was also amplified. In *Streptomyces*, there exist only one membrane-bound transhydrogenase, instead of two enzymes in *E. coli*[31]. The pyridine nucleotide transhydrogenase (PNT) enzymes are composed of α and β subunits encoded by the *pntA* and *pntB* genes, respectively. In our work, PNT enzymes were introduced into the native strain. The specific transhydrogenase activity assay showed that the HT-PNT possessed 0.98 ± 0.02 U/mg at 72 h, compared with 0.07 ± 0.02 U/mg in parent strain D852 (Table 1). Besides, the differences in the concentrations of four redox cofactors were observed during the exponential growth phase (Table 2). The NADPH/NADP^+^ ratio was approximately 20-fold higher than the NADH/NAD^+^ ratio in HT-PNT (Table 2). The NADPH/NADP^+^ ratio in HT-PNT was increased from 2.63 to 4.66, while the NADH/NAD^+^ ratio was decreased from 0.36 to 0.23 compared with D852, indicating that the transhydrogenase converted more NADH into NADPH. Furthermore, the concentration of NADPH in HT-PNT increased, while the concentration of NADH decreased, compared with D852. However, the total concentration of four redox cofactors remained unchanged. It should be noted that biomass synthesis was enhanced up to 10.2 ± 0.4 g/L in HT-PNT. Besides, within 72 h, HT-PNT produced 83.9 ± 9.9 mg/L FK506 (Figure 6) with the specific production rate of 2.34 ± 0.11 μmol/g DCW/h (Additional file 3: Table S1).

The third target gene *accA2* was overexpressed in D852, generating HT-ACC strain. Although the enzyme activity assay was repeated several times with independent-prepared extracts, acetyl-CoA carboxylase (ACC) in mutant strain HT-ACC and the parent strain D852 could not be detected (Table 1), which might be due to the lower value of detection limit. Even so, the intracellular metabolite profile displayed an expected increase (approximately three-fold) in intracellular malonyl-CoA concentration compared with D852 (Figure 5). On the contrary, the level of acetyl-CoA in HT-ACC was only 25% of the parent strain (Figure 5), suggesting that overexpressing ACC promoted the transformation of acetyl-CoA to malonyl-CoA for FK506 biosynthesis. Moreover, overexpression of *accA2* resulted in an approximately 40% improvement in FK506 production (200.5 ± 15.1 mg/L at 6 d), in comparison with the levels in D852 (143.5 ± 8.7 mg/L at 6 d) (Figure 6). Different from HT-DAHP and HT-PNT, no difference was observed in biomass yield between HT-ACC and D852 (Figure 6), indicating that overexpressing the *accA2* gene did not influence the cell growth.

The last modification gene *zwf2* was chosen as a target to amplify in D852. As expected, the G6PDH activity of HT-ZWF at 72 h and 144 h was 0.29 ± 0.04 and 0.15 ± 0.05 U/mg protein, respectively, which was increased by 123.1% and 87.5% compared with D852 (0.13 ± 0.03 and 0.08 ± 0.02 U/mg protein) (Table 1). The fermentation profiles showed that overexpression of *zwf2* resulted in an approximately 35% improvement of FK506 production (up to 193.2 ± 12.1 mg/L at 6 d), whereas a slight improvement of biomass concentration was achieved in *zwf2* overexpressed recombinant (up to 9.1 ± 0.2 g/L), compared with D852 (Figure 6). In addition, the glycolytic intermediates such as glycerate-1,3-bisphosphate and PEP in the mutant strain HT-ZWF decreased by 38% and 25%, respectively, in comparison with D852, as shown in Figure 5. On the contrary, the corresponding PPP intermediates such as E4P concentration in HT-ZWF was about 3-fold higher than that of the parent strain D852 owing to the increase of enzyme activity of G6PDH. Moreover, since enhancing the PPP can increase the intracellular reducing power NADPH, similarly with HT-PNT, a 33.5% increase in NADPH concentration was achieved in HT-ZWF (Table 2). The NADPH/NADP^+^ ratio was also increased from 2.63 to 6.71, but the NADH/NAD^+^ ratio was almost kept constant, compared with D852, indicating that the *zwf2* gene overexpression had no influence on NAD(H) balance.

#### Effect of combined gene knockout and overexpression on FK506 synthesis

**Figure 7:**
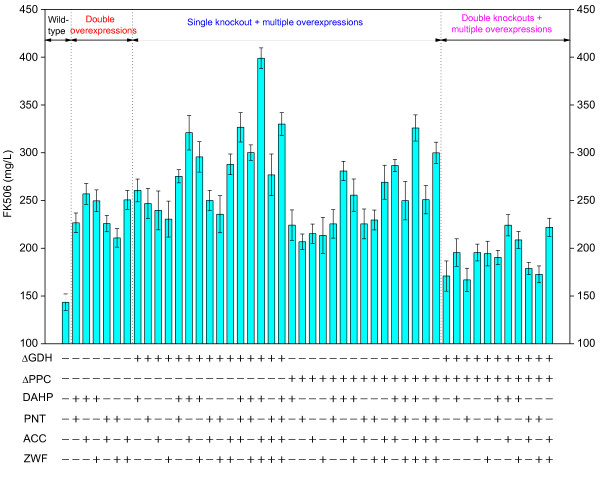
**The effect of combined gene knockout and overexpression on FK506 production.** Plus or minus symbols denote presence or absence of the indicated gene(s) manipulation. The data are the average values of at least three series of three parallel tests, and error bars represent standard deviations.

Since the single knockout or overexpression experiment improved FK506 production at various levels, the cumulative effect of multiple genetic manipulations on FK506 production was carried out by combining the gene knockout and overexpression. Here we explored the performance of these constructed strains to gain a better understanding of the underlying mechanisms about which pathway governed the FK506 biosynthesis. As shown in Figure 7, when the single gene knockout combined with the other overexpression genes, the ΔGDH mutants could produce higher titer of FK506 than that of ΔPPC, consistent with the *in silico* prediction (Figure 3C). It was suggested that the *gdhA* inactivation generated a positive effect in the combined strain. Furthermore, the strains which possessed an extra *pntAB* copy produced lower FK506 than the mutants without it, indicating that the other genes manipulation have saturated the NADPH capability available for FK506 production and further modification may generate the detrimental side effects. From another perspective, the above results implied that the redox balance played a key role in FK506 production and *pntAB* gene just converted the excess NADH into NADPH when necessary. Particularly, the strain overexpressing *dahp*, *accA2* and *zwf2* in the ΔGDH mutant background was able to produce 398.9 ± 14.8 mg/L FK506, a 1.8-fold increase compared with the parent strain D852. In addition, combination of double knockouts by HT-ΔGP resulted in as low as 67% of the FK506 production level achieved by HT-ΔGDH. Moreover, additional modifications did not further increase FK506 production compared with HT-ΔGDH-DAZ.

### Metabolic characterization of HT-ΔGDH-DAZ strain in fed-batch fermentation

**Figure 8:**
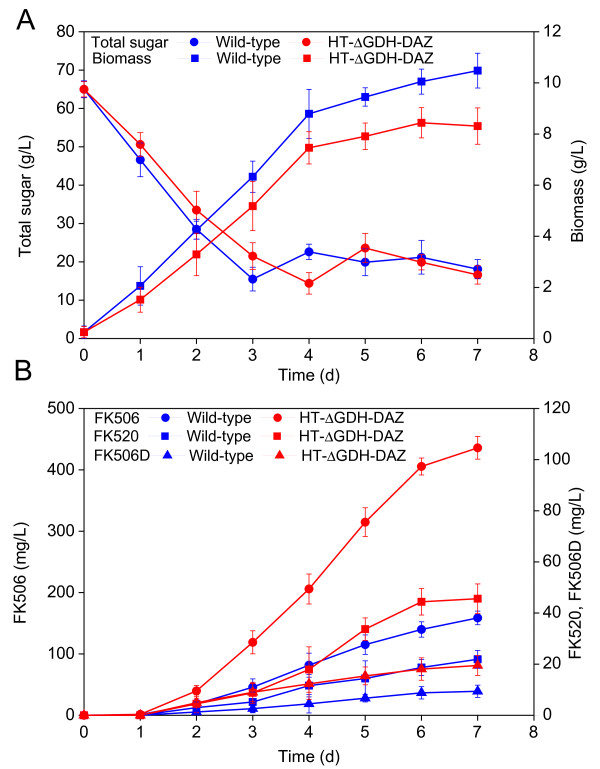
**The production performance profiles of wild-type strain D852 and engineered strain HT-ΔGDH-DAZ in fed-batch fermentation.** (**A**) Total sugar and biomass profiles; (**B**) FK506 and by-products profiles. The data are the average values of at least three series of three parallel tests, and error bars represent standard deviations.

To evaluate the potential FK506 production capacity for the constructed strain HT-ΔGDH-DAZ, fed-batch fermentation was carried out. Result showed that the substrate uptake rate and biomass concentration of HT-ΔGDH-DAZ were 0.44 ± 0.02 g/L/h and 8.5 ± 0.6 g/L, compared with the parent strain D852 (0.52 ± 0.03 g/L/h and 10.5 ± 0.7 g/L) (Figure 8A). The HT-ΔGDH-DAZ accumulated 435.9 ± 18.5 mg/L FK506 during the stationary phase, 1.47-fold higher than the parent strain D852 (158.7 ± 11.1 mg/L) (Figure 8B). These results demonstrated that FK506 biosynthetic performance of the rational engineered HT-ΔGDH-DAZ strain could be further improved during the fed-batch fermentation. Furthermore, it was observed that the production of by-products FK520 and 37,38-dihydro-FK506 (FK506D) in HT-ΔGDH-DAZ were also increased by approximately 1.1-fold, up to 45.6 ± 5.8 mg/L and 22.4 ± 4.3 mg/L, respectively, compared with the values of D852 (21.9 ± 3.5 mg/L and 12.2 ± 3.4 mg/L). It was considered that the improvement of by-products resulted from the common precursors and similar pathways with FK506 under the same genetic context and culture condition [32].

## Discussion

Despite many efforts aimed at optimizing different microorganisms for industrial FK506 production [8,9], there has been no report of a genetically engineered FK506 over-producer that can compete with the traditionally producing strains. GSMM provides an approach for strain optimization—systems metabolic engineering. The approach can be used to identify the bottleneck in the intracellular behaviors thus improve product yield on the basis of metabolic engineering. Specifically, the GSMM can facilitate the *in silico* prediction of previously unknown key metabolic nodes, thus enable synthesize more precursors and cofactors towards FK506. In fact, this strategy has been successfully used to identify target genes for the improvement of biochemical products [23,24,33,34]. In the present work, the GSMM of *S. tsukubaensis* were used to identify gene targets for improved FK506 production.

Among the initially predicted targets, NADPH-dependent glutamate dehydrogenase encoded by *gdhA* and phosphoenolpyruvate carboxylase encoded by *ppc* were chosen as knockout targets. The introduced genetic modification of *gdhA* influenced several fluxes through the central carbon metabolism (Figure 2). One of these flux changes was the reaction supplying cofactor NADPH for FK506. The pathway has been manipulated to improve important secondary metabolites such as lycopene and sesquiterpene [11,35]. Although the *gdhA* plays a key role in the ammonium uptake and amino acids metabolism, the carbon flux through GDH2 compensates for the loss of GDH1. In this study, HT-ΔGDH showed an approximately 40% increase in FK506 titer compared with D852. In this regard, deletion of *gdhA* could lead to a new redistribution of carbon flux that could be partially diverted into FK506 biosynthesis instead of glutamate family biosynthetic pathway, thus acquire an overall increase in yield.

Deletion of *ppc* reduced the flux from PEP to oxaloacetate [36]. Due to a decreased flux through PPC, the net flux from PEP towards shikimate pathway increased, which directed more precursors towards FK506 biosynthesis (Figure 2). From this perspective, the substantial PPC flux in D852 blocked efficient carbon conversion to DHCHC precursor for FK506 production. Thus, it was necessary to delete the PPC encoding gene *ppc*. In fact, it has been demonstrated that PPC is not an indispensable enzyme in *Corynebacterium glutamicum*[37]. Besides, in *B. subtilis* pyruvate carboxylase can replace PPC to maintain the anaplerotic reaction [38]. In our study, the *ppc* disruption resulted in an approximately 45% increase in the final FK506 titer. However, deletion of this gene also caused a 24% decrease in the biomass compared with D852, indicating that the pathway exerted a negative effect on the primary metabolism [39]. Moreover, it was observed that when the two selected knockout genes were manipulated in combination, the FK506 production decreased, compared with the single knockout strain (HT-ΔGDH or HT-ΔPPC) (Figure 7). It may be primarily ascribed to the unbalanced metabolism between cell growth and product formation, which would be explored in our future research.

In addition, several overexpression targets were identified using the algorithm raised by [24]. Overexpression of *dahp* gene showed an advantageous effect on the final FK506 titer and a slight effect on the cell growth. Interestingly, *dahp* overexpression has been previously applied to improve balhimycin yields by enhancing shikimate pathway [40]. However, the yield increment was not so much as predicted by the algorithm. In addition to the reasons of prediction, the bottleneck may be that in the primary metabolism aromatic amino acid biosynthesis is strictly controlled by feedback inhibition mechanisms. In *E. coli*, there are three isoenzymes of DAHP synthase, encoded by *aroF* (tyrosine-sensitive), *aroG* (phenylalanine-sensitive), and *aroH* (tryptophan-sensitive) [41]. The *aroF* and *aroG* can be inhibited by only 0.1 mM of tyrosine and phenylalanine, respectively. Thus, it is necessary to apply specific metabolic adaptations to resist feedback inhibition [42]. Fortunately, it has been revealed that some antibiotic biosynthetic clusters contain another similar gene, which is not inhibited by aromatic amino acids [43-45]. Therefore, it provides an opportunity to further optimize FK506 production.

The direct precursors of FK506 biosynthesis are malonyl-CoA, methylmalonyl-CoA, methoxymalonyl-ACP and allymalonyl-CoA [7,46,47]. Therefore, the predicted *accA2* gene was overexpressed, as it catalyzed the conversion of acetyl-CoA to malonyl-CoA (Figure 2). In *S. tsukubaensis*, the acetyl-CoA pool as well as sources of malonyl-CoA are limited. Overexpression of ACC enhanced the biosynthesis of malonyl-CoA (Figure 5), leading to the high production of FK506. Overexpression of ACC has also resulted in an improved malonyl-CoA biosynthesis and flavanone overproduction [34]. Malonyl-CoA availability has been shown to be a rate-limiting factor for many chain initiation and elongation reactions such as fatty acid, flavonoid and polyketide biosynthesis [48,49]. These facts clearly showed the beneficial effect of *accA2* overexpression in enhancing the malonyl-CoA pool available for FK506 biosynthesis.

With the aid of *f*_PH_ value, targets *pntAB* and *zwf2* were identified to play an important role in regulating the redox metabolism. They were both responsible for the provision of NADPH as a cofactor of FK506 biosynthesis. In order to improve NADPH availability, a NADP-dependent glyceraldehyde-3-phosphate dehydrogenase from *Clostridium acetobutylicum* was introduced into *E. coli* to replace the native NAD-dependent enzyme, which exerted an improvement in the lycopene and ϵ-caprolactone production [50]. In addition, it was reported that inactivation of the phosphofructokinase gene (*pfkA*) directed more carbon flow towards the PPP, leading to actinorhodin and undecylprodigiosin overproduction in *S. coelicolor* A3(2) [51]. In our work, the *zwf2* and *pntAB* genes were overexpressed both individually and combinatorially. The single overexpression strain (HT-PNT or HT-ZWF) produced an improved FK506 in comparison to wild-type strain D852. Consequently, it was considered that enhancing NADPH availability may improve flux towards the FK506 biosynthesis pathway. However, the recombinant strain HT-PZ expressing both genes showed lower concentration than HT-PNT or HT-ZWF (Figure 7). This may be attributed to the fact that additional redox gene caused the metabolic imbalance which was closely related with the energy, amino acids, lipids and nucleotides metabolism [52]. It was indicated that synthesis of FK506 may be strictly regulated by the intracellular NADPH level. In fact, it has been reported that sufficient NADPH concentrations and proper redox balance are necessary for NADPH-dependent biosynthetic processes [53]. Thus, it seemed that reducing equivalents were not transferred from NADH to NADPH via PNT in HT-PZ, and the net reducing equivalents available for FK506 production could not exceed a certain value. These results demonstrated that, on one hand, overexpression of gene *pntAB* or *zwf2* increased NADPH availability; on the other hand, a proper and balanced redox state was necessary for the efficient FK506 production.

Combination of multiple genes knockout and overexpression manipulation was also analyzed in this study. The performance of these strains could aid in understanding the relationship between the impact of single gene manipulation and combinatorial genes manipulation on the FK506 biosynthesis pathway. As shown in Figure 7, the *gdhA* knockout mutants combined with the other overexpression genes were beneficial to the improvement of FK506 synthesis, while *ppc* mutants combinations enhanced FK506 production to a lesser extent. The rational designed strain *S. tsukubaensis* HT-ΔGDH-DAZ with overexpression of *dahp*, *accA2*, *zwf2* and deletion of *gdhA* was a promising cell factory which may be applied to produce FK506 efficiently in industry fermentation. The strain HT-ΔGDH-DAZ displayed a high specific FK506 production rate (2.65 and 2.74 μmol/g DCW/h for 500-mL flask batch culture and 3-L bioreactor fed-batch cultivation, respectively). Besides, the genetic stability result indicated that the hereditary character of the strain HT-ΔGDH-DAZ was stable. Therefore, in the laboratory-scale, our engineered strain HT-ΔGDH-DAZ can compete with traditional overproducers. It was interesting to see that impact of multiple genes modification on metabolic characteristic of strain may be related to the interactions among the interconnected pathways. Recently, this observation has been clearly elucidated to be the synergistic effect (positive or negative cooperativity) [54], which would guide the selection of combinatorial genes modification for strain optimization.

Based on the experimental validation, the predicted gene targets directed more carbon flux or redox cofactor towards FK506 production. However, the observed improvement in the specific FK506 production rate (Additional file 3: Table S1) was much lower compared to the predicted improvement. These differences might be due to the fact that our metabolic network did not take into account the kinetic and thermodynamic constraints on the possible flux changes, the interaction of pathways as well as complex regulatory mechanisms. Therefore, there existed some limitations in our constraint-based metabolic model. Even so, the observed improvement in the FK506 production proved the validity of the metabolic engineering target. The intracellular metabolite data obtained from this study can be used to investigate the thermodynamic and kinetic parameters of the pathways, which are imposed as additional constraints on the model for the next round of metabolic engineering. Besides, since the true pathway fluxes are still unknown, it is necessary to quantify intracellular fluxes by ^13^C-metabolic flux analysis, which will further improve algorithm predictability.

## Conclusions

In this work, the GSMM-guided metabolic engineering strategy was employed to improve the FK506 production of *S. tsukubaensis*. Based on the single gene knockout and overexpression simulation, potential targets (*gdhA* and *ppc* for knockout; *dahp*, *pntAB*, *accA2* and *zwf2* for overexpression) were identified. The strains were designed according to each predicted target. Fermentation characterization of the engineered strains with single gene knockout or overexpression showed the improved capacities of FK506 production. Moreover, the combinatorial genes modification indicated that strain HT-ΔGDH-DAZ with *gdhA*-deletion and *dahp*-, *accA2*-, *zwf2*-overexpression produced 435.9 mg/L FK506 during fed-batch fermentation, 1.47-fold higher than the parent strain D852 (158.7 mg/L). Our results demonstrate the validity of application of *in silico* modelling tools for biopharmaceuticals overproduction. The intracellular metabolite data would be used for computational analysis to investigate kinetics and thermodynamics of the pathways. The precursors or cofactors availability would be further investigated to regulate the accumulation of intermediates and to enhance efficient conversion into FK506. Moreover, since FK506 biosynthetic cluster influenced the titre, enhancing the catalysed efficiency of the cluster might be essential for future work.

## Materials and methods

### Genome-scale metabolic network reconstruction

The procedure of the genome-scale metabolic network reconstruction for the *S. tsukubaensis* can be described as follows. The gene products of *S. tsukubaensis* were analyzed and annotated function by sequence alignment against protein sequences from related organisms based on the sequence homology search. In this work, a functional sequence by the bidirectional BLASTp was screened after attaining three thresholds: e-value ≤ 10^-30^, matching length ≥ 70% of the query sequence, amino acid sequence identity ≥ 40%. Various isoenzymes or enzyme complexes were identified with KEGG, resulting in the initial network. Public databases, such as KEGG, BRENDA, UniProt and MetaCyc, were used to manually refine the draft metabolic network, including addition of specific reactions, deletion of incorrect reactions, balance of mass and charge information, filling of metabolic gaps. Some reactions which were obtained from the experimental data and published literature were also supplemented in the network. Besides, biosynthetic reactions for the biomass and FK506 were included. Since there was no detailed information on the biomass composition of *S. tsukubaensis*, the protein, DNA, RNA, lipids, small molecules and cell wall components (peptidoglycan, carbohydrate and teichoic acid) were partly measured and partly referred from literature data. As for the FK506 synthesis, the synthesis of specific precursors and overall reaction were added (Figure 1B). The detailed information of the biomass composition and FK506 synthesis are described in (Additional file 1: Text S1). The genes, proteins, reactions, and metabolites are listed in (Additional file 2: Text S2) in detail.

### Computational procedure

The resulting model was analyzed using Constraint-Based Reconstruction and Analysis (COBRA). Gene knockout or overexpression prediction was performed using the COBRAToolbox-2.0 in MATLAB, with GLPK and CPLEX as the optimization programming solvers [55].

For the knockout targets identification, FBA algorithm was first employed to obtain an initial flux distribution with the maximization of the specific growth rate as the objective function. The constrains contained experimental specific consumption and production rates. Second, using MOMA algorithm [16], the knockout targets which resulted in a higher specific FK506 production rate than the value of the parent strain D852 were identified. Here, metabolic flux distribution of knockout strain changed minimal with respect to the flux distribution of the parent strain. The knockout targets were screened according to the *in silico* specific FK506 production rate.

For the overexpression targets identification, the algorithm developed by [24] was employed. In brief, an FBA problem with the maximization of the specific growth rate was calculated, generating an initial flux distribution for the whole network. This step was the same as the first step of knockout simulation. Subsequently, each non-zero reaction flux was amplified to some extent (for instance 2-fold), and the quadratic programming problem was solved by MOMA. The overexpression targets were identified through comparing a fraction value, *f*_PH_ (the ratio of weighted and dimensionless specific growth rate and specific FK506 production rate).

$$\begin{array}{l} f_{\mathrm{PH}}\equiv \left(f_{\text{biomass}}\right)\left(f_{\mathrm{FK}506}\right)=\left(\frac{v_{\text{biomass},\text{overexpression}}}{v_{\text{biomass},\text{wild}}}\right)\\ \;\times \left(\frac{v_{\mathrm{FK}506,\text{overexpression}}}{v_{\mathrm{FK}506,\text{wild}}}\right) \end{array}$$

Overexpressing genes that had the higher *f*_PH_ were the better candidates to manipulate experimentally.

### Bacterial strains, plasmids and cultivation conditions

All strains and plasmids used in this study are summarized in (Additional file 5: Table S2). The parent strain *S. tsukubaensis* D852 was a stock of our laboratory and deposited in China General Microbiological Culture Collection Center with the accession number CGMCC 7180. All *Streptomyces* mutants were derived from D852. *E. coli* JM109 was used to propagate all plasmids. *E. coli* ET12567/pUZ8002 was used as the nonmethylating plasmid donor strain [56] for intergeneric conjugation with *S. tsukubaensis* D852. *E. coli* strains were cultured in Luria-Bertani (LB) medium at 37°C. The integrative *E. coli*–*Streptomyces* vector pIB139 containing the ermE* promoter (P_ermE_*) [57] was used for gene overexpression in *S. tsukubaensis* D852, and *E. coli*–*Streptomyces* vector pKC1139 [56] was used for in-frame gene knockout. Spores and seed culture of *S. tsukubaensis* D852 were prepared as described by [56,58]. Batch cultivation for FK506 production was carried out by inoculating 1 mL of seed culture into 100 mL of production medium in a 500-mL flask at pH 7.0 and then culturing at 220 rpm for 6 days at 28°C. The production medium contained 60 g/L starch, 2 g/L yeast extract, 2.5 g/L peptone, 5 g/L soybean meal, 0.5 g/L K_2_HPO_4_, 0.5 g/L CaCO_3_, 0.5 g/L MgSO_4_, pH 7.0. Fed-batch fermentation was performed at 28°C in a 3-L BIOFLO 110 bioreactor (New Brunswick Scientific Company, USA) with the working volume of 1.5 L. The aeration rate was set to 1 vvm by a mass flow controller. The dissolved oxygen level was kept above 20% of air saturation by automatically regulating the agitation speed. The pH was controlled at 6.8 by automatic addition of 0.5 M HCl and 0.5 M NaOH. Antibiotics were added appropriately as follows: ampicillin 100 μg/mL, apramycin 50 μg/mL, thiostrepton 20 μg/mL, kanamycin 25 μg/mL and chloromycin 25 μg/mL. *S. coelicolor* A3(2) and *S. roseosporus* ATCC 11379 were cultivated in YEME for genomic DNA isolation [56].

### Gene cloning, plasmid construction and transformation

All DNA manipulations were performed according to the standard protocols [59]. All primers used in this work are listed in (Additional file 5: Table S3). DAHP synthase gene (*aroH*, here we defined *dahp*) (accession number ZP_04707751) and ACC gene (*accA2*) (accession number ZP_04710929) were amplified from *S. roseosporus* ATCC11379 genomic DNA. PNT gene (*pntAB*) (accession number CAC16724 and CAC16725) and G6PDH gene (*zwf2*) (accession number CAB50762) were amplified from *S. coelicolor* genome. Each gene was digested by *Nde*I–*Xba*I and cloned into pIB139 to yield pDAHP, pACC, pPNT, pZWF, respectively.

To delete the *Bam*HI restriction site, pUC18 was excised with *Bam*HI, linearized, blunt-ended and ligated, generating pUC18M. For the construction of pDP carrying double genes, PCR product of *dahp* was excised with *Nde*I–*Xba*I and transferred to the same sites of pUC18M, generating pUC18M-D. Then PCR product of *pntAB* was digested with *Bgl*II–*Xba*I and transferred to the *Bam*HI–*Xba*I sites of pUC18M-D, generating pUC18M-DP. The *Nde*I–*Xba*I fragment of the *dahp* and *pntAB* genes were excised from the pUC18M-DP and ligated into pIB139 to yield pDP. The other plasmids pDA, pDZ, pPA, pPZ and pAZ were constructed using the similar method. For the plasmids construction of three genes overexpression, pUC18M-DP, pUC18M-DA and pUC18M-PA were digested with *Bam*HI–*Xba*I and ligated with *Bgl*II–*Xba*I of PCR product containing the *accA2*, *zwf2* gene, respectively, yielding pUC18M-DPA, pUC18M-DPZ, pUC18M-DAZ and pUC18M-PAZ. Then the resulting plasmids were excised with *Nde*I–*Xba*I and the long fragment containing the three genes were transferred to the same sites of pIB139. The plasmid pUC18M-DPA was excised with *Bam*HI–*Xba*I and ligated with *Bgl*II–*Xba*I of PCR product containing the *zwf2* gene. The generated pUC18M-DPAZ was excised with *Nde*I–*Xba*I and inserted into the same sites of pIB139. Each constructed plasmid was transferred into *E. coli* ET12567/pUZ8002, which was subsequently introduced into *S. tsukubaensis* D852 through conjugal transfer [56]. The positive exconjugants were verified by PCR amplification and DNA sequencing with primer pair pIB-F/pIB-R.

To delete NADPH-dependent GDH1 gene *gdhA*, deletion plasmid was constructed by amplifying the upstream and downstream flanking regions from genomic DNA of *S. tsukubaensis* (accession number EIF89180). the upstream and downstream flanking regions of *gdhA* were amplified using the primers *gdhA*-LF/*gdhA*-LR and *gdhA*-RF/*gdhA*-RR, respectively. The above two fragments were excised with *Xba*I–*Bam*HI and *Kpn*I–*Eco*RI, respectively, ligated sequentially into the pUC119-Kan^R^ which possessed the kanamycin resistance cassette. After digestion with the restriction enzymes *Xba*I–*Eco*RI, the fragments were ligated into pKC1139 that had been digested with the same restriction enzymes, resulting in the deletion plasmid pΔGDH (Additional file 5: Table S3). This plasmid was then transferred into *S. tsukubaensis* D852 using the procedure as described above. The double crossover mutant was selected as described previously [32], verified by PCR amplification and DNA sequencing. As for PPC gene *ppc*, the method was similar with the *gdhA* with the exception that thiostrepton resistance cassette was used as the selected marker.

For complementation experiment, the pGDH and pPPC plasmids were constructed according to the procedure similar with the pDAHP and subsequently introduced into the HT-ΔGDH and HT-ΔPPC, respectively.

### Analytical methods

The biomass was determined by filtering 10 mL fermentation broth through a pre-weighed 0.45 μm pore size filter (Satorius), washing twice and drying at 80°C to constant weight. The residual total sugar in fermentation broth was quantified using phenol-sulfuric acid method [60]. For the detection of FK506 as well as the by-products FK520 and FK506D, the sample was mixed with equal volume of methanol, shaken intermittently in water bath at 50°C for 2 h. After centrifugation at 6,000 × g for 10 min, the supernatant was analyzed by HPLC equipped with a Venusil XDB-C_18_ column (5 μm, 250 mm × 4.6 mm). The mobile phase contained 0.1% phosphoric acid in water and acetonitrile (35:65, v/v). The flow rate was 1 mL/min, the column temperature was 60°C and the detection wavelength was 210 nm.

### Quantification of intracellular metabolites

The intracellular metabolites were determined in the exponential phase in batch cultivations by triplicate. For each point, 10 mL of culture broth was immediately filtered through a 0.8 μm pore size cellulose acetate membrane. On the filter disc, the cells were subsequently washed with 10 mL 0.9% (w/v) NaCl solution. Next, the filter was immediately transferred into a 50 mL tube containing 25 mL of pre-chilled methanol solution (60%, v/v) (−40°C) to stop cell metabolism. The above procedure was controlled within 10–15 s. Subsequently, the mixture was centrifuged at 3,000 × g for 10 min at −20°C. After removing the supernatant, 2.5 mL cold aqueous methanol solution (60%, v/v, −40°C) was added to the sample tube and stored at −80°C until metabolites extraction. The metabolites were extracted by thawing the above samples in an ice bath for 4 min. Then the mixture was vigorously shaken for 1 min and frozen for 30 min at −80°C. After repeating three times of freeze-thaw, the sample was then centrifuged at 10,000 × g for 10 min at −20°C and the supernatant was used for quantifying metabolites. Intracellular metabolites of central carbon metabolism (glycolysis, PPP and TCA cycle) and cofactors were determined by liquid chromatography-tandem quadrupole mass spectrometry, as described by [61]. Amino acids and CoA esters were measured by gas chromatography–mass spectrometry and liquid chromatography-electrospray ionization-tandem mass spectrometry, as described by [62] and [63], respectively. The measurements of intracellular metabolites were normalized to the biomass.

### Analysis of in vitro enzyme activities

The culture samples were harvested at the exponential phase (72 h) and the stationary phase (144 h), and prepared as our previous procedures [30]. The standard assay for DAHP synthase activity was spectrophotometrically (549 nm) monitored by following the oxidation with NaIO_4_ and reaction with thiobarbituric acid at 100°C [64]. As a membrane-bound protein, the PNT activity was determined in cell extracts without centrifugation. The activity of PNT was measured spectrophotometrically at 375 nm by the reduction of 3-acetylpyridine-NAD^+^ as reported previously [65]. ACC activity was measured as described by [66]. G6PDH activity assay was performed based on the NADPH synthesis according to the method of [67]. GDH1 and GDH2 were determined by oxidizing NADPH or NADH at 340 nm in the presence of α-ketoglutarate and ammonia [68]. PPC activity was measured spectrophotometrically by monitoring the oxidation of NADH in a coupled assay with malate dehydrogenase [69]. PK activity was monitored by the decrease in absorbance of NADH at 340 nm, with lactate dehydrogenase as the coupling enzyme [70]. PC was assayed by coupling the oxidation reaction of NADH with malate dehydrogenase towards the oxaloacetate synthesis and measured spectrophotometrically at 340 nm [71]. Total protein concentrations were quantified by Bradford assay with a reagent solution (Quick Start Bradford Dye, BioRad). The assays of enzyme activities were normalized to the total protein.

## Competing interests

The authors declare that they have no competing interests.

## Authors’ contributions

DH conceived and designed the research, and constructed the metabolic network model. DH performed the main experiments, the statistical analysis and drafted the manuscript. DH, SSL and MLX performed strain cultivation, enzyme assays, sample preparation and metabolites analysis. DH, SSL and XQJ contributed to manuscript revision. JPW supervised the research and revised the manuscript. All authors read and approved the final manuscript.

## Supplementary Material

Additional file 1 **Text S1.** The detailed descriptions of the biomass composition and FK506 biosynthesis by *S. tsukubaensis*. Click here for file

Additional file 2 **Text S2.** The reconstructed genome-scale metabolic model of *S. tsukubaensis*, including reactions and metabolites. Click here for file

Additional file 3: Table S1 The specific FK506 production rate of various engineered strains. Click here for file

Additional file 4: Figure S1 The effect of glutamate or glutamine addition on cell growth for HT-ΔGDH strain. **Figure S2.** The effect of succinate, fumarate or malate addition on cell growth for HT-ΔPPC strain. Click here for file

Additional file 5: Table S2 Strains and plasmids used in this study. **Table S3.** Primers used in this work. Click here for file
