# Supplementary material for: Genome-scale metabolic network guided engineering of Streptomyces tsukubaensis for FK506 production improvement
Source: Microb Cell Fact. 2013 May 24;12:52. doi: 10.1186/1475-2859-12-52 (PMC3680238; doi:10.1186/1475-2859-12-52)
Supplement: Additional file 1 — Text S1. The detailed descriptions of the biomass composition and FK506 biosynthesis by S. tsukubaensis. [file 1475-2859-12-52-S1.pdf]

Additional file 2

Text S1. The detailed descriptions of the biomass composition and FK506 biosynthesis by *S. tsukubaensis*

1. Biomass composition

The macromolecular composition of the cell was partly measured and partly estimated from literature data. Dry weight was measured by washing cells with 0.9% NaCl solution and drying them at 80 ℃ on a pre-weighted filter until a steady weight was achieved. Biomass components contain protein, RNA, DNA, lipids, small molecules, cell wall components (peptidoglycan, carbohydrate and teichoic acid). Total protein was determined using the Bradford method [1], which was estimated based on a standard curve of bovine serum albumin (25–200 μg/mL). The protein content of samples for our experiments was within the range 40–50% of dry cell mass. Pellet hydrolysates were used to measure amino acid composition. The amino acid composition of the protein fraction has also been determined by Agilent HPLC systems (Agilent 1200, USA) with Agilent Zorbax Eclipse column (4.6 × 150 mm). No differences were observed in composition within the dilution rate range of 0.1-0.5 h^{-1}. Total carbohydrate was measured by the phenol/sulfuric acid method [2]. Cellular composition was estimated based on a standard curve of glucose (10–200 μg/mL). The carbohydrate content of samples was within the range 10–15% of dry cell mass, thus 1250 μg/mL of freeze-dried biomass was used in order to acquire a carbohydrate concentration that would be within the mid-standard range. Total DNA was determined by the diphenylamine method using a hot perchloric acid extraction of freshly-harvested biomass [3]. Samples were estimated based on a calf-thymus DNA standard solutions (100–500 μg/mL). Total RNA content was determined using an KOH/UV-based procedure based on calf liver RNA standard solutions (100–500 μg/mL) [4]. Ash composed of inorganic compounds and ions, as well as intracellular metabolites, were not taken into account in the model. The biomass equation consists of biomass components and the growth-associated ATP consumption. Based on the data shown in Table S1.1, the biomass equation used in the model is:

0.446 protein + 0.163 RNA + 0.042 DNA + 0.027 phospholipid + 0.018 triacylglycerol + 0.030 small molecules + 0.137 peptidoglycan + 0.0082 teichoic acids + 0.055 carbohydrate + 47 ATP -> biomass + 47 ADP + 47 Pi

The stoichiometric coefficients have unit g/g DCW.

Table S1.1: Macromolecular composition (biomass formation) of *S. tsukubaensis*.

**Biomass component**	**Component (g/g DCW)**
Protein	0.4460[a]
DNA	0.0420[a]
RNA	0.1630[a]
Phospholipid	0.0273[b]
Triacylglycerol	0.0182[b]
Small molecules	0.0300[b]
Peptidoglycan	0.1368[b]
Carbohydrate	0.0547[a]
Teichoic acid	0.0820[b]

[a] Calculated for an average macromolecular composition of *S. tsukubaensis* with starch as carbon sources and few yeast extract, peptone as nitrogen sources. Biomass composition was experimentally measured at the exponential growth phase of aerobic batch cultivation (specific growth rate: 0.0502 h^{-1}).

[b] Data was summarized from [5,6].

The amino acid composition was determined by an Agilent HPLC systems at 354 nm.

Table S1.2: Protein composition of *S. tsukubaensis*.

**Amino acid type**	**Molecular weight (g/mol)**	**Composition (%, g/g protein)**	**Composition (mmol/g)**
Alanine	71.09	10.52	1.508
Arginine	156.20	3.89	0.256
Asparagine	114.12	5.08	0.445
Aspartate	115.10	5.15	0.448
Cysteine	103.15	2.02	0.196
Glutamate	129.13	7.80	0.612
Glutamine	128.15	4.41	0.345
Glycine	57.07	7.66	1.378
Histidine	137.16	2.66	0.194
Isoleucine	113.17	6.82	0.603
Leucine	113.17	3.84	0.339
Lysine	128.19	2.22	0.173
Methionine	131.21	3.84	0.301
Phenylalanine	147.19	4.83	0.328
Proline	97.13	8.80	0.916
Serine	87.09	4.51	0.518
Threonine	101.12	6.55	0.658
Tryptophan	186.23	1.48	0.097
Tyrosine	163.19	2.59	0.159
Valine	99.15	5.33	0.539

Energy requirement for polymerization (mmol ATP/g) = 40.04

The DNA composition was calculated based on the genome information of *S. tsukubaensis*. GC content of *S. tsukubaensis* is 71.5%, which is then used to calculate the ratio of the nucleic acids in the DNA. The polymerization energy was assumed to be the same as in *E. coli* [7].

Table S1.3: DNA composition of *S. tsukubaensis*.

DNTP type	Molecular weight (g/mol)	Composition (%mol)	Composition (mmol/g)
DAMP	313.24	14.25	0.4611
DCMP	289.18	35.75	1.1567
DTMP	304.20	14.25	0.4611
DGMP	329.21	35.75	1.1567

Energy requirement for polymerization (mmol ATP/g) = 4.4

The RNA composition was determined from genomic data assuming the following composition: 5% mRNA, 75% rRNA and 20% tRNA [5]. The polymerization energy was assumed to be the same as *E. coli* [7].

Table S1.4: RNA composition of *S. tsukubaensis*.

	mol/mol RNA					
**NTP type**	**mRNA 5%**	**rRNA 75%**	**tRNA 20%**	**Molecular weight(g/mol)**	**Composition (mol/mol RNA)**	**Composition (mmol/g RNA)**
AMP	0.139	0.195	0.198	329.21	0.192	0.600
GMP	0.361	0.238	0.342	345.21	0.265	0.826
CMP	0.361	0.340	0.289	305.18	0.331	1.031
UMP	0.139	0.228	0.171	306.17	0.212	0.662

Energy requirement for polymerization (mmol ATP/g) = 1.25

The composition of phospholipids was assumed to be the same as *E. coli*. Biosynthesis of phospholipids and fatty acids components are included in the reaction set.

Table S1.5: Phospholipid composition of *S. tsukubaensis*.

Phospholipids	Molecular weight[a] (g/mol)	Composition[b] (g/g)	Composition (mmol/g)
Phosphatidylethanolamine	690.84	0.75	1.086
Cardiolipin	1349.59	0.07	0.052
Phosphatidylinositol	824.90	0.18	0.218

Energy requirement for polymerization (mmol ATP/g) = 2.63

[a] The molecular weight was calculated from chemical structure of the phospholipid by assuming an average molecular weight of different fatty acids.

[b] Three components in phospholipids were assumed to be the same with the compositions existing in *S. coelicolor* model [5].

Table S1.6: Fatty acid composition of *S. tsukubaensis* for synthesis of phospholipid.

Fatty acids	Molecular weight (g/mol)	Composition[a] %(w/w)	Composition (mmol/g)	Composition (mol/mol)
C14:0	227.37	1.2	0.05	0.013
C15:0	241.40	42.6	1.77	0.450
C16:0	255.43	11.3	0.44	0.113
C17:0	269.45	44.5	1.65	0.421
C18:1	267.44	0.4	0.01	0.004

[a] Data was taken from *S. coelicolor* composition [5].

Table S1.7: Triacylglycerol composition of *S. tsukubaensis*.

Fatty acids	Molecular weight (g/mol)	Composition[a] (mol/mol)	Composition (mmol/g)
Glycerol-3-phosphate	174.08	1	1.244
C14:0	227.37	0.040	0.050
C15:0	241.40	1.349	1.677
C16:0	255.43	0.338	0.421
C17:0	269.45	1.262	1.570
C18:1	267.44	0.011	0.014

Energy requirement for polymerization (mmol ATP/g) = 1.24

[a] Data was taken from *S. coelicolor* composition [5].

It was assumed that the selected small molecules were equally presented (w/w) in the pool.

Table S1.8: Small molecules pool composition of *S. tsukubaensis*.

**Molecules**	**Molecular weight (g/mol)**	**Pool of small molecules (g/g)**	**Composition (mmol/g)**
NAD	664.438	0.125	0.188
NADP	744.418	0.125	0.168
COA	767.534	0.125	0.163
ACP	10689	0.125	0.012
MK	853.365	0.125	0.146
THF	445.434	0.125	0.281
FMN	456.348	0.125	0.274
FAD	785.557	0.125	0.159

Table S1.9: Peptidoglycan composition of *S. tsukubaensis*.

Peptidoglycan composition	Molecular weight[a] (g/mol)	Ratio[b] (mol/mol)	Composition (mmol/g)
N-acetylmuramic acid	275.26	0.88	1.0069
N-acetylglucosamine	203.19	1.05	1.1969
Alanine	71.08	1.67	1.8999
Diaminopimelic acid	154.07	1.00	1.1399
D-glutamate	129.12	0.89	1.0145
Glycine	39.04	0.85	0.9727

Energy requirement for polymerization (mmol ATP/g) = 5.026

[a] Glycine and diaminopimelic acid were required for peptidoglycan polymerization with molecule of water. Other components were required without molecule of water.

[b] Data was taken from average molecular composition of peptidoglycan from *S. coelicolor* [5].

Table S1.10: Carbohydrate composition of *S. tsukubaensis*.

Carbohydrate composition	Molecular weight (g/mol)	Ratio[a] (mol/mol)	Composition (mmol/g)
UDP-N-acetylglucosamine	203.19	1	1.897
UDP-D-galactose	162.14	2	3.794

[a] Data was taken from *S. coelicolor* composition [5].

Table S1.11: Teichoic acids composition of *S. tsukubaensis*.

Teichoic acids composition	Molecular weight (g/mol)[a]	Ratio[b] (mol/mol)	Composition (mmol/g)
Polyglycerophosphate chain	1848.71	1	0.5177
Lysine	128.17	0.25	0.1294
N-acetylglucosamine	203.19	0.25	0.1294

Energy requirement for polymerization (mmol ATP/g) = 0.129

[a] The molecular weight of polyglycerophosphate chain was calculated using $(C_3H_7O_5P)_{12}$ as molecular formula [5].

[b] Data was taken from *S. coelicolor* composition [5].

2. FK506 biosynthesis

Biosynthesis of FK506 macrolide is composed of polyketide synthase (PKS) and nonribosomal peptide synthetase (NRPS), similar with FK520 and rapamycin. Generally, FkbA, FkbB and FkbC modules consist of three common elements, including ketoacylsynthase, acyltransferase (AT) and acyl carrier protein (ACP), which are responsible for chain elongation. Some modules also contain dehydratase (DH), enoylreductase (ER) and ketoreductase (KR), which catalyze the reduction reactions. After using a chorismate-derived 4,5-dihydroxycyclohex-1-enecarboxylic acid (DHCHC) as the starter unit, the PKSs assemble ten two-carbon extensions into chains [8]. The ten extender units contain two malonyl-CoA, five methylmalonyl-CoA, two methoxymalonyl-ACP and an unusual allylmalonyl-CoA. After the condensation, a lysine-derived pipecolate catalyzed by FkbL, incorporates into the resulting polyketide intermediate with an amide bond [9]. This reaction is catalyzed by the FkbP peptide synthetase, which cyclizes the 23-membered core. For the last step, post-PKS processing reactions are catalyzed by a specific 31-O-methyl transferase FkbM and a P_{450} C_9 hydroxylase FkbD.

For ten extender precursors, the biosynthetic pathways are different. Malonyl-CoA can be transformed from acetyl-CoA by acetyl-CoA carboxylase, similar with fatty acid biosynthesis pathway. For methylmalonyl-CoA synthesis, there are four metabolic pathways according to presented literatures. First, propionyl-CoA can be catalyzed to methylmalonyl-CoA by propionyl-CoA carboxylase [10,11]. Second, conversion of succinyl-CoA to methylmalonyl-CoA is catalyzed by two steps, encoded by methylmalonyl-CoA mutase and epimerase, respectively [12,13]. Next, isobutyryl-CoA which is originated from valine catabolism need several steps of oxidation (3-methyl-2-oxobutanoate dehydrogenase, butyryl-CoA dehydrogenase, 3-hydroxyacyl-CoA dehydrogenase, methylmalonate-semialdehyde dehydrogenase, aldehyde dehydrogenase) [14]. Lastly, the exogenous methylmalomate can be transported into cells via dicarboxylate carrier protein and malonyl/methylmalonyl-CoA synthetase pathway [15]. Of course, the last step is also applied to malonyl-CoA due to the shared enzyme.

Besides, the PKSs in FK506 biosynthesis incorporate two unusual extender units named methoxymalonyl-ACP and allylmalonyl-CoA. The methoxymalonyl-ACP biosynthesis utilizes glycolytic pathway intermediate 1,3-biphosphoglycerate as the starter. An unusual ACP protein FkbJ is loaded onto glyceroyl by FkbH, generating glyceroyl-ACP. Subsequently, two oxidases, FkbK (oxidizes 3-hydroxyacyl-CoA compounds to 3-oxoacyl-CoA compounds, mediated by NAD^+) and FkbI (oxidizes saturated acyl-CoA compounds to 2,3-enoyl-CoA compounds, mediated by FAD), and an O-methyltransferase FkbG modify the glyceroyl-ACP to methoxymalonyl-ACP [16]. The *tcsABCD* identified from the FK506 biosynthetic gene cluster have been demonstrated to catalyze the allylmalonyl-CoA synthesis [17,18]. In detail, the propionyl-acylated TcsB catalyzes the condensation of the propionyl-CoA with malonate loaded on TcsA, generating 3-oxopentanoyl-ACP, and subsequently is converted to 2-pentenyl-ACP by KR and DH of fatty acid synthase. Then, the 2-pentenyl-ACP is further reduced by TcsC (mediated by NADPH) following a carboxylation step. Last step is a dehydrogenation by TcsD (mediated by FAD), generating the allylmalonyl-ACP, which is subsequently converted to allylmalonyl-CoA, a unique extender unit for the FK506 side chain. Similar with the allylmalonyl-CoA, the propylmalonyl-CoA is biosynthesized by TcsA, TcsB and TcsC, but without TcsD [17,18]. The TcsC also catalyzes crotonyl-CoA to ethylmalonyl-CoA by a similar way [18]. The propylmalonyl-CoA and ethylmalonyl-CoA can be incorporated into the initial polyketide chain to form 37,38-dihydro-FK506 and FK520, respectively.

Detailed reactions can be found in Additional file 3.

References

1. Bradford MM: **A rapid and sensitive method for the quantitation of microgram quantities of protein utilizing the principle of protein-dye binding.** *Anal Biochem* 1976, **72:**248-254.
2. Herbert D, Phipps PJ, Strange RE: **Chapter III Chemical Analysis of Microbial Cells.** In *Methods in Microbiology. Volume* Volume 5, Part B. Edited by Norris JR, Ribbons DW: Academic Press; 1971: 209-344
3. Burton K: **A study of the conditions and mechanism of the diphenylamine reaction for the colorimetric estimation of deoxyribonucleic acid.** *Biochem J* 1956, **62:**315-323.
4. Benthin S, Nielsen J, Villadsen J: **A simple and reliable method for the determination of cellular RNA content.** *Biotechnol Tech* 1991, **5:**39-42.
5. Borodina I, Krabben P, Nielsen J: **Genome-scale analysis of *Streptomyces coelicolor* A3(2) metabolism.** *Genome Res* 2005, **15:**820-829.
6. Olukoshi ER, Packter NM: **Importance of stored triacylglycerols in *Streptomyces*: possible carbon source for antibiotics.** *Microbiology* 1994, **140:**931-943.
7. Ingraham JL, Maaløe O, Neidhardt FC: **Growth of the bacterial cell.** *Sinauer Associates, Inc, Massachusetts* 1983.
8. Andexer JN, Kendrew SG, Nur-e-Alam M, Lazos O, Foster TA, Zimmermann AS, Warneck TD, Suthar D, Coates NJ, Koehn FE, et al: **Biosynthesis of the immunosuppressants FK506, FK520, and rapamycin involves a previously undescribed family of enzymes acting on chorismate.** *Proc Natl Acad Sci U S A* 2011, **108:**4776-4781.
9. Gatto GJ, Boyne MT, Kelleher NL, Walsh CT: **Biosynthesis of pipecolic acid by RapL, a lysine cyclodeaminase encoded in the rapamycin gene cluster.** *J Am Chem Soc* 2006, **128:**3838-3847.
10. Bramwell H, Hunter IS, Coggins JR, Nimmo HG: **Propionyl-CoA carboxylase from *Streptomyces coelicolor* A3(2): cloning of the gene encoding the biotin-containing subunit.** *Microbiology* 1996, **142 (Pt 3):**649-655.
11. Rodríguez E, Gramajo H: **Genetic and biochemical characterization of the α and β components of a propionyl-CoA carboxylase complex of *Streptomyces coelicolor* A3(2).** *Microbiology* 1999, **145:**3109-3119.
12. Reeves AR, Brikun IA, Cernota WH, Leach BI, Gonzalez MC, Weber JM: **Effects of methylmalonyl-CoA mutase gene knockouts on erythromycin production in carbohydrate-based and oil-based fermentations of *Saccharopolyspora erythraea*.** *J Ind Microbiol Biotechnol* 2006, **33:**600-609.
13. Reeves AR, Brikun IA, Cernota WH, Leach BI, Gonzalez MC, Mark Weber J: **Engineering of the methylmalonyl-CoA metabolite node of *Saccharopolyspora erythraea* for increased erythromycin production.** *Metab Eng* 2007, **9:**293-303.
14. Tang L, Zhang YX, Hutchinson CR: **Amino acid catabolism and antibiotic synthesis: valine is a source of precursors for macrolide biosynthesis in *Streptomyces ambofaciens* and *Streptomyces fradiae*.** *J Bacteriol* 1994, **176:**6107-6119.
15. Murli S, Kennedy J, Dayem LC, Carney JR, Kealey JT: **Metabolic engineering of *Escherichia coli* for improved 6-deoxyerythronolide B production.** *J Ind Microbiol Biotechnol* 2003, **30:**500-509.
16. Wu K, Chung L, Revill WP, Katz L, Reeves CD: **The FK520 gene cluster of *Streptomyces hygroscopicus* var. ascomyceticus (ATCC 14891) contains genes for biosynthesis of unusual polyketide extender units.** *Gene* 2000, **251:**81-90.

17. Mo S, Kim DH, Lee JH, Park JW, Basnet DB, Ban YH, Yoo YJ, Chen SW, Park SR, Choi EA, et al: **Biosynthesis of the allylmalonyl-CoA extender unit for the FK506 polyketide synthase proceeds through a dedicated polyketide synthase and facilitates the mutasynthesis of analogues.** *J Am Chem Soc* 2011, **133:**976-985.
18. Kosec G, Goranovič D, Mrak P, Fujs Š, Kuščer E, Horvat J, Kopitar G, Petković H: **Novel chemobiosynthetic approach for exclusive production of FK506.** *Metab Eng* 2012, **14:**39-46.
