# Supplementary material for: Genome-scale metabolic network guided engineering of Streptomyces tsukubaensis for FK506 production improvement
Source: Microb Cell Fact. 2013 May 24;12:52. doi: 10.1186/1475-2859-12-52 (PMC3680238; doi:10.1186/1475-2859-12-52)
Supplement: Additional file 2 — Text S2. The reconstructed genome-scale metabolic model of S. tsukubaensis, including reactions and metabolites. [file 1475-2859-12-52-S2.pdf]

Additional file 3

Text S2. The reconstructed genome-scale metabolic model of *S. tsukubaensis*, including reactions and metabolites.

Detailed reactions list

**Reaction Number**	**Pathway**	**EC-number**	**Enzyme definition**	**Reversibility**	**Gene**	**Reaction**
1	Glycolysis	2.7.1.2	glucokinase-glc	IRR		1 GLC + 1 ATP -> 1 G6P + 1 ADP
2	Glycolysis	2.7.1.2	glucokinase-bglc	IRR		1 bGLC + 1 ATP -> 1 bG6P + 1 ADP
3	Glycolysis	5.4.2.2	Phosphoglucomutase_g1p	REV	pgm	1 G1P -> 1 G6P
4	Glycolysis	5.3.1.9	Glucose-6-phosphate isomerase_1	REV	pgi	1 G6P -> 1 F6P
5	Glycolysis	5.3.1.9	Glucose-6-phosphate isomerase_2	REV	pgi	1 bG6P -> 1 G6P
6	Glycolysis	5.3.1.9	Glucose-6-phosphate isomerase_3	REV	pgi	1 bG6P -> 1 F6P
7	Glycolysis	2.7.1.11	6-phosphofructokinase	IRR	(pfkA3 OR pfkA OR pfkA2)	1 ATP + 1 F6P -> 1 FDP + 1 ADP
8	Glycolysis	3.1.3.11	Fructose-bisphosphatase	IRR	glpX	1 FDP + 1 H2O -> 1 F6P + 1 PI
9	Glycolysis	4.1.2.13	Fructose-bisphosphate aldolase	REV	fba	1 FDP -> 1 DHAP + 1 GAP
10	Glycolysis	5.3.1.1	Triosephosphate isomerase	REV	tpiA	1 GAP -> 1 DHAP
11	Glycolysis	1.2.1.12	Glyceraldehyde 3-phosphate dehydrogenase (phosphorylating)	REV	(gap1 OR gap2 OR gap2)	1 GAP + 1 PI + 1 NAD -> 1 DPG + 1 NADH + 1 H
12	Glycolysis	2.7.2.3	Phosphoglycerate kinase	REV	pgk	1 DPG + 1 ADP -> 1 3PG + 1 ATP
13	Glycolysis	5.4.2.1	Phosphoglycerate mutase	REV	(pgm2 OR pgm)	1 3PG -> 1 2PG

14	Glycolysis	4.2.1.11	Phosphopyruvate hydratase	REV	(eno OR eno2)	1 2PG -> 1 PEP + 1 H2O
15	Glycolysis	2.7.1.40	Pyruvate kinase	IRR	(pyk1 OR pyk2)	1 PEP + 1 ADP -> 1 PYR + 1 ATP
16	Pyruvate metabolism	1.1.1.28	D-lactate dehydrogenase	REV	(dldh2 OR dldh)	1 NADH + 1 PYR + 1 H -> 1 NAD + 1 LAC
17	Pyruvate metabolism	6.2.1.1	acetate--CoA ligase	IRR	acsA	1 COA + 1 AC + 1 ATP -> 1 ACCOA + 1 PPI + 1 AMP
18	Pyruvate metabolism	1.1.1.38	Malate dehydrogenase (oxaloacetate decarboxylating)	IRR		1 NAD + 1 MAL -> 1 NADH + 1 CO2 + 1 PYR + 1 H
19	Pyruvate metabolism	1.1.1.40	Malate dehydrogenase (oxaloacetate decarboxylating) (NADP+)	IRR		1 NADP + 1 MAL -> 1 NADPH + 1 CO2 + 1 PYR + 1 H
20	Pyruvate metabolism	2.7.9.2	Pyruvate,H2O dikinase	IRR		1 PYR + 1 ATP + 1 H2O -> 1 PI + 1 PEP + 1 AMP
21	Pyruvate metabolism	2.7.9.1	Pyruvate,phosphate dikinase	IRR		1 PYR + 1 ATP + 1 PI -> 1 PEP + 1 AMP + 1 PPI
22	Pyruvate metabolism	4.1.1.31	Phosphoenolpyruvate carboxylase	IRR	ppc	1 PEP + 1 CO2 + 1 H2O -> 1 PI + 1 OA
23	Pyruvate metabolism	4.1.1.49	Phosphoenolpyruvate carboxykinase (ATP)	IRR		1 OA + 1 ATP -> 1 CO2 + 1 PEP + 1 ADP
24	Pyruvate metabolism	1.1.1.1	alcohol dehydrogenase	REV		1 ACAL + 1 NADH + 1 H -> 1 ETH + 1 NAD
25	Pyruvate metabolism	1.1.1.2	Alcohol dehydrogenase (NADP+)	REV		1 NADP + 1 ETH -> 1 NADPH + 1 ACAL + 1 H
26	Pyruvate metabolism	1.2.2.2	Pyruvate dehydrogenase (cytochrome)	IRR		1 PYR + 1 UBIQON + 1 H2O -> 1 UBIQOL + 1 AC + 1 CO2
27	Pyruvate	1.2.1.3	Aldehyde dehydrogenase (NAD+)	IRR	thcA	1 ACAL + 1 NAD + 1 H2O -> 1 AC + 1 NADH + 1 H

	metabolism					
28	Pyruvate metabolism	2.7.2.1	Acetate kinase	REV	ackA	1 AC + 1 ATP -> 1 ACETYLEP + 1 ADP
29	Pyruvate metabolism	2.3.1.8	Phosphate acetyltransferase	REV	pta	1 PI + 1 ACCOA -> 1 ACETYLEP + 1 COA
30	Pyruvate metabolism	3.6.1.7	Acylphosphatase	IRR		1 PI + 1 AC -> 1 ACETYLEP + 1 H2O
31	Pyruvate metabolism	1.2.4.1	pyruvate dehydrogenase (acetyl-transferring)	IRR	aceE1	1 PYR + 1 LIPO -> 1 ADLIPO + 1 CO2
32	Pyruvate metabolism	2.3.1.12	dihydrolipoyllysine-residue acetyltransferase	REV	sucB	1 COA + 1 ADLIPO -> 1 DLIPO + 1 ACCOA
33	Pyruvate metabolism	6.4.1.2	putative acetyl CoA carboxylase (alpha and beta subunits)	REV	accA2	1 HCO3 + 1 H + 1 ACCOA + 1 ATP -> 1 MALCOA + 1 PI + 1 ADP
34	Pyruvate metabolism	6.4.1.2	putative acetyl CoA carboxylase (alpha and beta subunits)	REV	accA2	1 CCCP + 1 ACCOA + 1 H -> 1 MALCOA + 1 BCCP
35	TCA cycle	6.4.1.1	Pyruvate carboxylase	IRR	pyc	1 HCO3 + 1 PYR + 1 ATP -> 1 PI + 1 OA + 1 ADP
36	TCA cycle	4.1.1.32	Phosphoenolpyruvate carboxykinase (GTP)	IRR		1 CO2 + 1 PEP + 1 GDP -> 1 OA + 1 GTP
37	TCA cycle	2.3.3.1	Citrate (si)-synthase	IRR	citA	1 OA + 1 ACCOA + 1 H2O -> 1 CIT + 1 COA
38	TCA cycle	4.1.3.6	Citrate lyase	IRR		1 CIT -> 1 AC + 1 OA
39	TCA cycle	4.2.1.3	Citrate lyase	REV	sacA	1 CIT -> 1 ICIT
40	TCA cycle	1.1.1.42	Isocitrate dehydrogenase (NADP+)	REV	idh	1 ICIT + 1 NADP -> 1 NADPH + 1 AKG + 1 CO2 + 1 H
41	TCA cycle	1.2.4.2	Oxoglutarate dehydrogenase (lipoamide)	IRR	kgd	1 AKG + 1 LIPO -> 1 SDLIPO + 1 CO2
42	TCA cycle	2.3.1.61	Dihydrolipoamide S-succinyltransferase	IRR	sucB	1 COA + 1 SDLIPO -> 1 DLIPO + 1 SUCCOA
43	TCA cycle	1.2.7.3	2-oxoglutarate synthase	IRR		2 FERI + 1 COA + 1 AKG -> 2 FERO + 1 CO2 + 1 SUCCOA + 2 H

44	TCA cycle	6.2.1.5	succinyl-CoA synthetase (ADP-forming)	REV	(sucC AND sucD)	1 SUCCOA + 1 ADP + 1 PI -> 1 SUCC + 1 COA + 1 ATP
45	TCA cycle	1.3.99.1	Succinate dehydrogenase	REV	(sdhC OR sdhB OR sdhA)	1 SUCC + 1 FAD -> 1 FUM + 1 FADH2
46	TCA cycle	1.3.99.1	Succinate dehydrogenase	REV	(sdhC OR sdhB OR sdhA)	1 SUCC + 1 UBIQON -> 1 FUM + 1 UBIQOL
47	TCA cycle	4.2.1.2	Fumarate hydratase	REV	(fumC OR fumB)	1 FUM + 1 H2O -> 1 MAL
48	TCA cycle	1.1.1.37	Malate dehydrogenase	REV	mdh	1 MAL + 1 NAD -> 1 OA + 1 NADH + 1 H
49	Glyoxylate shunt	4.1.3.1	Isocitrate lyase	IRR	aceA	1 ICIT -> 1 GLX + 1 SUCC
50	Glyoxylate shunt	2.3.3.9	malate synthase	IRR	(aceB2 OR aceB1)	1 ACCOA + 1 GLX + 1 H2O -> 1 MAL + 1 COA
51	PPP	1.1.1.47	D-glucose dehydrogenase (NAD(P)+)	IRR		1 GLC + 1 NAD -> 1 GLCLAC + 1 NADH
52	PPP	3.1.1.17	Gluconolactonase	IRR		1 GLCLAC + 1 H2O -> 1 GLUC
53	PPP	2.7.1.12	Gluconokinase	IRR		1 ATP + 1 GLUC -> 1 D6PGC + 1 ADP
54	PPP	1.1.1.49	Glucose-6-phosphate 1-dehydrogenase	REV	(zwf2 OR zwf)	1 G6P + 1 NADP -> 1 D6PGL + 1 NADPH + 1 H
55	PPP	3.1.1.31	6-phosphogluconolactonase	IRR	pgl	1 D6PGL + 1 H2O -> 1 D6PGC
56	PPP	1.1.1.44	phosphogluconate dehydrogenase	IRR	gnd	1 D6PGC + 1 NAD -> 1 RL5P + 1 CO2 + 1 NADH + 1 H
57	PPP	5.1.3.1	ribulose phosphate 3-epimerase	REV	rpe	1 RL5P -> 1 X5P
58	PPP	5.3.1.6	Ribose 5-phosphate epimerase	REV	rpi	1 RL5P -> 1 R5P
59	PPP	5.4.2.2	Phosphoglucomutase_r1p	REV	pgm	1 R5P -> 1 R1P
60	PPP	5.4.2.2	Phosphoglucomutase_dr1p	REV	pgm	1 R5P -> 1 DR1P
61	PPP	2.2.1.1	Transketolase	REV	tktA2	1 R5P + 1 X5P -> 1 S7P + 1 GAP
62	PPP	2.2.1.1	Transketolase	REV	tatB	1 X5P + 1 E4P -> 1 F6P + 1 GAP
63	PPP	2.2.1.2	Transaldolase	REV	tal1 OR tal2	1 GAP + 1 S7P -> 1 F6P + 1 E4P
64	PPP	2.7.1.15	ribokinase	IRR		1 RIB + 1 ATP -> 1 R5P + 1 ADP

65	PPP	2.7.1.15	Ribokinase	IRR		1 DRIB + 1 ATP -> 1 DR5P + 1 ADP
66	PPP	4.1.2.4	Deoxyribose-phosphate aldolase	IRR		1 DR5P -> 1 ACAL + 1 GAP
67	PPP	4.1.2.13	fructose-bisphosphate aldolase_2	REV	fba	1 E4P + 1 GAP -> 1 S17BP
68	PPP	3.1.3.37	Phosphoric-monoester hydrolases	IRR		1 S17BP + 1 H2O -> 1 S7P + 1 PI
69	PPP	2.7.1.45	2-dehydro-3-deoxygluconokinase	IRR		1 KDG + 1 ATP -> 1 KDPG + 1 ADP
70	PPP	4.1.2.14	2-dehydro-3-deoxyphosphogluconate aldolase	IRR		1 KDPG -> 1 GAP + 1 PYR
71	Glutamate, glutamine biosynthesis	6.3.1.2	glutamine synthetase	IRR		1 NH3 + 1 GLT + 1 ATP -> 1 GLN + 1 ADP + 1 PI
72	Glutamate, glutamine biosynthesis	1.4.1.4	Glutamate dehydrogenase (NADP+)	REV	gdhA	1 NH3 + 1 AKG + 1 NADPH + 1 H -> 1 GLT + 1 NADP + 1 H2O
73	Glutamate, glutamine biosynthesis	1.4.1.2	Glutamate dehydrogenase (NAD+)	REV	gdh2	1 NH3 + 1 AKG + 1 NADH + 1 H -> 1 GLT + 1 NAD + 1 H2O
74	Glutamate, glutamine biosynthesis	1.4.1.14	glutamate synthase (NADH)	REV		2 GLT + 1 NAD -> 1 GLN + 1 AKG + 1 NADH + 1 H
75	Glutamate, glutamine biosynthesis	1.4.1.13	glutamate synthase (NADPH)	REV		1 GLN + 1 AKG + 1 NADPH + 1 H -> 2 GLT + 1 NADP
76	Glutamate, glutamine biosynthesis	3.5.1.2	Glutaminase	IRR		1 GLN + 1 H2O -> 1 NH3 + 1 GLT
77	Proline	2.7.2.11	Glutamate 5-kinase	IRR	ProB	1 GLT + 1 ATP -> 1 GLU5P + 1 ADP

	biosynthesis					
78	Proline biosynthesis	1.2.1.41	Glutamate-5-semialdehyde dehydrogenase	IRR	proA	1 GLU5P + 1 NADPH + 1 H -> 1 GLUGSAL + 1 PI + 1 NADP
79	Proline biosynthesis	spnts4	spontaneous	IRR		1 GLUGSAL -> 1 P5C + 1 H2O
80	Proline biosynthesis	1.5.1.2	pyrroline-5-carboxylate reductase	IRR		1 P5C + 1 NADPH + 1 NADH -> 1 PRO + 1 NADP + 1 NAD
81	Arginine biosynthesis	2.3.1.1	Amino-acid N-acetyltransferase	IRR	argJ	1 GLT + 1 ACCOA -> 1 NAGLU + 1 COA
82	Arginine biosynthesis	2.7.2.8	Acetylglutamate kinase	IRR	argB	1 NAGLU + 1 ATP -> 1 NAGLUP + 1 ADP
83	Arginine biosynthesis	1.2.1.38	N-acetyl-gamma-glutamyl-phosphate reductase	REV	argC	1 NAGLUP + 1 NADPH + 1 H -> 1 NAGLUS + 1 NADP + 1 PI
84	Arginine biosynthesis	2.6.1.11	Acetylornithine aminotransferase	REV	argD	1 NAGLUS + 1 GLT -> 1 NAACORN + 1 AKG
85	Arginine biosynthesis	2.3.1.35	Glutamate N-acetyltransferase	REV	argJ	1 GLT + 1 NAACORN -> 1 AGLU + 1 ORN
86	Arginine biosynthesis	2.1.3.3	Ornithine carbamoyltransferase	REV	arcB	1 ORN + 1 CAP -> 1 CITR + 1 PI
87	Arginine biosynthesis	6.3.4.5	argininosuccinate synthetase	IRR	argG	1 ASP + 1 CITR + 1 ATP -> 1 ARGSUCC + 1 PPI + 1 AMP
88	Arginine biosynthesis	4.3.2.1	Argininosuccinate lyase	REV	argH	1 ARGSUCC -> 1 ARG + 1 FUM
89	Aspartate biosynthesis	2.6.1.1	Aspartate aminotransferase	REV	aspC	1 OA + 1 GLT -> 1 ASP + 1 AKG

90	Asparagine biosynthesis	6.3.5.4	asparagine synthetase (glutamine-hydrolysing)	IRR		1 GLN + 1 ASP + 1 ATP + 1 H2O -> 1 GLT + 1 ASN + 1 PPI + 1 AMP
91	Homoserine biosysnthesis	2.7.2.4	Aspartate kinase	REV	ask	1 ASP + 1 ATP -> 1 BASP + 1 ADP
92	Homoserine biosysnthesis	1.2.1.11	Aspartate-semialdehyde dehydrogenase	REV	asd1	1 NADPH + 1 BASP + 1 H -> 1 NADP + 1 PI + 1 ASPSA
93	Homoserine biosysnthesis	1.1.1.3	Homoserine dehydrogenase	REV	thrA	1 NADPH + 1 ASPSA + 1 H -> 1 NADP + 1 HOMOSER
94	Lysine biosynthesis	4.2.1.52	dihydrodipicolinate synthase	IRR		1 PYR + 1 ASPSA -> 2 H2O + 1 DDCOL
95	Lysine biosynthesis	1.3.1.26	Dihydrodipicolinate reductase	IRR	dapB	1 DDCOL + 1 NADPH + 1 H -> 1 D1PDICARBOXYLATE + 1 NADP
96	Lysine biosynthesis	2.3.1.117	2,3,4,5-tetrahydropyridine-2-carboxylate N-succinyltransferase	IRR		1 D1PDICARBOXYLATE + 1 SUCCOA + 1 H2O -> 1 SAOPIM + 1 COA
97	Lysine biosynthesis	2.6.1.17	Succinyldiaminopimelate aminotransferase	REV	argD	1 GLT + 1 SAOPIM -> 1 AKG + 1 SDAPIM
98	Lysine biosynthesis	3.5.1.18	Succinyl-diaminopimelate desuccinylase	IRR	dapE	1 SDAPIM + 1 H2O -> 1 DAPIM + 1 SUCC
99	Lysine biosynthesis	5.1.1.7	Diaminopimelate epimerase	REV	dapF	1 DAPIM -> 1 MDAPIM
100	Lysine biosynthesis	4.1.1.20	Diaminopimelate decarboxylase	IRR	lysA	1 MDAPIM -> 1 CO2 + 1 LYS
101	Methionine biosynthesis	2.3.1.46	homoserine O-succinyltransferase	IRR		1 HOMOSER + 1 SUCCOA -> 1 OSLHSER + 1 COA
102	Methionine biosynthesis	2.5.1.48	cystathionine gamma-synthase	IRR	metB	1 CYS + 1 OSLHSER -> 1 CYSTATHIONINE + 1 SUCC

103	Methionine biosynthesis	2.5.1.48	cystathionine gamma-synthase	IRR	metB	1 OSLHSER + 1 H2S -> 1 HOMOCYC + 1 SUCC
104	Methionine biosynthesis	4.4.1.8	Cystathionine beta-lyase	IRR		1 CYSTATHIONINE + 1 H2O -> 1 NH3 + 1 PYR + 1 HOMOCYC
105	Methionine biosynthesis	2.1.1.10	L-homocysteine S-methyltransferase	IRR	metH	1 HOMOCYC + 1 SAMET -> 1 MET + 1 SAH
106	Methionine biosynthesis	2.1.1.13	5-methyltetrahydrofolate--homocysteine S-methyltransferase	IRR	metH	1 HOMOCYC + 1 MTHF -> 1 MET + 1 THF
107	Methionine biosynthesis	2.1.1.14	5-methyltetrahydropteroyltriglutamate--homocysteine S-methyltransferase	IRR	metE	1 M5THTGLU + 1 HOMOCYC -> 1 THTGLU + 1 MET
108	Threonine biosynthesis	2.7.1.39	Homoserine kinase	IRR	thrB	1 HOMOSER + 1 ATP -> 1 PHSER + 1 ADP
109	Threonine biosynthesis	4.2.3.1	Threonine synthase	IRR		1 PHSER + 1 H2O -> 1 PI + 1 THR
110	Threonine biosynthesis	4.1.2.5	L-Allothreonine acetaldehyde-lyase	REV		1 GLY + 1 ACAL -> 1 THR
111	Serine biosynthesis	1.1.1.95	Phosphoglycerate dehydrogenase	IRR	serA	1 3PG + 1 NAD -> 1 PHP + 1 NADH + 1 H
112	Serine biosynthesis	2.6.1.52	Phosphoserine aminotransferase	REV		1 PHP + 1 GLT -> 1 SER3P + 1 AKG
113	Serine biosynthesis	3.1.3.3	Phosphoserine phosphatase	IRR		1 SER3P + 1 H2O -> 1 SER + 1 PI
114	Glycine biosynthesis	2.1.2.1	Glycine hydroxymethyltransferase	IRR	glyA1	1 SER + 1 THF -> 1 METHTHF + 1 GLY + 1 H2O
115	Cysteine biosynthesis	2.3.1.30	Serine O-acetyltransferase	REV		1 SER + 1 ACCOA -> 1 ASER + 1 COA

116	Cysteine biosynthesis	2.5.1.47	Cysteine synthase	IRR		1 ASER + 1 H2S -> 1 CYS + 1 AC
117	Cystine biosynthesis	4.2.1.22	Cystathionine beta-synthase	IRR		1 HOMOCYC + 1 SER -> 1 CYSTATHIONINE + 1 H2O
118	Cystine biosynthesis	4.4.1.1	Cystathionine gamma-lyase	IRR	cysA	1 CYSTATHIONINE + 1 H2O -> 1 NH3 + 1 OXOBUTANOATE + 1 CYS
119	Cystine biosynthesis	2.5.1.48	cystathionine gamma-synthase	IRR	metB	1 OSLHSER + 1 H2O -> 1 NH3 + 1 OXOBUTANOATE + 1 SUCC
120	Valine biosynthesis	2.2.1.6	Acetolactate synthase	IRR		2 PYR -> 1 ALAC + 1 CO2
121	Valine biosynthesis	1.1.1.86	Ketol-acid reductoisomerase	IRR	(ilvC OR ilvC2)	1 ALAC + 1 NADPH + 1 H -> 1 DIVALER + 1 NADP
122	Valine biosynthesis	4.2.1.9	Dihydroxy-acid dehydratase	IRR		1 DIVALER -> 1 KISOVALERATE + 1 H2O
123	Valine biosynthesis	2.6.1.42	Branched-chain amino acid aminotransferase	REV		1 KISOVALERATE + 1 GLT -> 1 VAL + 1 AKG
124	Isoleucine biosynthesis	4.3.1.19	Threonine dehydratase	IRR	ilvA	1 SER -> 1 PYR + 1 NH3
125	Isoleucine biosynthesis	4.3.1.19	Threonine dehydratase	IRR	ilvA	1 THR -> 1 OXOBUTANOATE + 1 NH3
126	Isoleucine biosynthesis	2.2.1.6	Acetolactate synthase	IRR		1 PYR + 1 OXOBUTANOATE -> 1 AHBUT + 1 CO2
127	Isoleucine biosynthesis	1.1.1.86	Ketol-acid reductoisomerase	IRR	ilvC	1 AHBUT + 1 NADPH + 1 H -> 1 KMV + 1 NADP
128	Isoleucine biosynthesis	4.2.1.9	Dihydroxy-acid dehydratase	IRR		1 KMV -> 1 KMVALERATE + 1 H2O

129	Isoleucine biosynthesis	2.6.1.42	Branched-chain amino acid aminotransferase	REV		1 KMVALERATE + 1 GLT -> 1 ILE + 1 AKG
130	Leucine biosynthesis	2.3.3.13	2-isopropylmalate synthase	IRR		1 KISOVALERATE + 1 ACCOA -> 1 CHISOCARPOATE + 1 COA
131	Leucine biosynthesis	4.2.1.33	3-isopropylmalate dehydratase	REV		1 CHISOCARPOATE + 1 H2O -> 1 THCISOCAPROATE
132	Leucine biosynthesis	1.1.1.85	3-isopropylmalate dehydrogenase	IRR		1 THCISOCAPROATE + 1 NAD -> 1 CMO + 1 NADH + 1 H
133	Leucine biosynthesis	spnts3	spontaneous	IRR		1 CMO -> 1 CO2 + 1 MPENTANOATE
134	Leucine biosynthesis	2.6.1.42	Branched-chain amino acid aminotransferase	REV	(SCL11.02c OR ilvE)	1 MPENTANOATE + 1 GLT -> 1 LEU + 1 AKG
135	Histidine biosynthesis	2.4.2.17	ATP phosphoribosyltransferase	IRR	hisG	1 ATP + 1 PRPP -> 1 PRBATP + 1 PPI
136	Histidine biosynthesis	3.6.1.31	Phosphoribosyl-ATP pyrophosphatase	IRR	hisE	1 PRBATP + 1 H2O -> 1 PRBAMP + 1 PPI
137	Histidine biosynthesis	3.5.4.19	Phosphoribosyl-AMP cyclohydrolase	IRR	hisL	1 PRBAMP + 1 H2O -> 1 PRFP
138	Histidine biosynthesis	5.3.1.16	N-(5'-phospho-D-ribosylformimino)-5-amino-1-(5"-phosphoribosyl)-4- imidazole carboxamide isomerase	IRR	priA	1 PRFP -> 1 PRLP
139	Histidine biosynthesis	2.4.2.-	imidazole glycerol phosphate synthase	IRR	hisH	1 PRLP + 1 GLN -> 1 DIMGP + 1 AICAR + 1 GLT
140	Histidine biosynthesis	4.2.1.19	Imidazoleglycerol-phosphate dehydratase	IRR	hisB	1 DIMGP -> 1 IMACP + 1 H2O

141	Histidine biosynthesis	2.6.1.9	Histidinol-phosphate aminotransferase	IRR	hisC3	1 IMACP + 1 GLT -> 1 HISOLP + 1 AKG
142	Histidine biosynthesis	3.1.3.15	Histidinol-phosphatase	IRR		1 HISOLP + 1 H2O -> 1 HISOL + 1 PI
143	Histidine biosynthesis	1.1.1.23	Histidinol dehydrogenase	IRR	hisD	1 HISOL + 2 NAD + 1 H2O -> 1 HIS + 2 NADH + 2 H
144	Shikimate pathway	2.5.1.54	2-dehydro-3-deoxyphosphoheptonate aldolase	IRR	aroH	1 PEP + 1 E4P + 1 H2O -> 1 DAHP + 1 PI
145	Shikimate pathway	4.2.3.4	3-dehydroquinate synthase	IRR	aroB	1 DAHP -> 1 PI + 1 DQT
146	Shikimate pathway	4.2.1.10	3-dehydroquinate dehydratase	REV	aroQ	1 DQT -> 1 DSHIK + 1 H2O
147	Shikimate pathway	1.1.1.25	Shikimate 5-dehydrogenase	REV	aroE	1 NADPH + 1 DSHIK + 1 H -> 1 NADP + 1 SME
148	Shikimate pathway	2.7.1.71	shikimate-kinase	IRR	aroK	1 SHE + 1 ATP -> 1 SME3P + 1 ADP
149	Shikimate pathway	2.5.1.19	3-phosphoshikimate 1-carboxyvinyltransferase	REV	aroA2	1 SME3P + 1 PEP -> 1 3PSME + 1 PI
150	Shikimate pathway	4.2.3.5	CHOR synthase	IRR	aroF	1 3PSME -> 1 PI + 1 CHOR
151	Phenylalanine, tyrosine biosynthesis	5.4.99.5	CHOR mutase	IRR		1 CHOR -> 1 PHEN
152	Phenylalanine, tyrosine biosynthesis	4.2.1.51	PHEN dehydratase	IRR	pheA	1 PHEN -> 1 PHPYR + 1 CO2 + 1 H2O

153	Phenylalanine, tyrosine biosynthesis	2.6.1.1	aspartate transaminase	REV		1 PHPYR + 1 GLU -> 1 AKG + 1 PHE
154	Phenylalanine, tyrosine biosynthesis	1.3.1.12	PHEN dehydrogenase	IRR		1 PHEN + 1 NAD -> 1 PHPPYR + 1 CO2 + 1 NADH + 1 H
155	Phenylalanine, tyrosine biosynthesis	2.6.1.1	aspartate transaminase	REV		1 PHPPYR + 1 GLU -> 1 AKG + 1 TYR
156	Tryptophan biosynthesis	4.1.3.27	Anthranilate synthase	IRR	(trpG,trpE2)	1 CHOR + 1 GLN -> 1 AN + 1 PYR + 1 GLT
157	Tryptophan biosynthesis	2.4.2.18	Anthranilate phosphoribosyltransferase	IRR	trpD1	1 AN + 1 PRPP -> 1 NPRAN + 1 PPI
158	Tryptophan biosynthesis	5.3.1.24	Phosphoribosylanthranilate isomerase	IRR	trpF	1 NPRAN -> 1 CPAD5P
159	Tryptophan biosynthesis	4.1.1.48	indole-3-glycerol-phosphate synthase	IRR	trpC1	1 CPAD5P -> 1 IGP + 1 CO2 + 1 H2O
160	Tryptophan biosynthesis	4.2.1.20	Tryptophan synthase alpha subunit	IRR	(trpA,trpB)	1 IGP + 1 SER -> 1 TRP + 1 GAP + 1 H2O
161	Tryptophan biosynthesis	4.2.1.20	Tryptophan synthase alpha subunit	IRR	(trpA,trpB)	1 IGP -> 1 INDOLE + 1 GAP
162	Tryptophan biosynthesis	4.2.1.20	Tryptophan synthase alpha subunit	IRR	(trpA,trpB)	1 INDOLE + 1 SER -> 1 TRP + 1 H2O
163	Glutamate metabolism	4.1.1.15	Glutamate decarboxylase	IRR	gad	1 GLT -> 1 CO2 + 1 GABA
164	Glutamate	2.6.1.19	4-aminobutyrate aminotransferase	IRR	gabT	1 AKG + 1 GABA -> 1 GLT + 1 SUCCALD

	metabolism					
165	Glutamate metabolism	2.6.1.19	4-aminobutyrate aminotransferase	IRR	gabT	1 GABA + 1 PYR -> 1 SUCCALD + 1 ALA
166	Glutamate metabolism	1.2.1.16	Succinate-semialdehyde dehydrogenase (NAD(P)+)	IRR	gabD1	1 SUCCALD + 1 NAD + 1 H2O -> 1 NADH + 1 SUCC + 1 H
167	Arginine metabolism	3.5.3.6	Arginine deiminase	IRR	arcA	1 ARG + 1 H2O -> 1 NH3 + 1 CITR
168	Arginine metabolism	4.1.1.19	Arginine decarboxylase	IRR		1 ARG -> 1 CO2 + 1 AGMATINE
169	Arginine metabolism	3.5.3.11	Agmatinase	IRR	speB	1 AGMATINE + 1 H2O -> 1 UREA + 1 PUTRESCINE
170	Arginine metabolism	2.6.1.13	Ornithine--oxo-acid aminotransferase	IRR	rocD	1 ORN + 1 AKG -> 1 GLT + 1 GLUGSAL
171	Spermine biosynthesis	4.1.1.17	Ornithine decarboxylase	IRR	SC1C3.23	1 ORN -> 1 CO2 + 1 PUTRESCINE
172	Spermine biosynthesis	2.5.1.16	Spermidine synthase	IRR		1 PUTRESCINE + 1 SAMETA -> 1 SPERMIDINE + 1 MTADENOSINE + 1 H
173	Urate metabolism	3.5.1.5	Urease	IRR	(ureD AND ureG AND ureF) OR (ureAB AND ureC AND ureC AND ureB AND ureA)	1 UREA + 1 H2O -> 2 NH3 + 1 CO2
174	Proline metabolism	1.5.1.2	pyrroline-5-carboxylate reductase	IRR	proC	1 P35C + 1 NADPH -> 1 HPRO + 1 NADP
175	Proline	4.1.2.14	4-hydroxy-2-oxoglutarate aldolase	IRR		1 HYDROXYAKG -> 1 GLX + 1 PYR

	metabolism					
176	Proline metabolism	2.6.1.1	Aspartate aminotransferase	REV	aspC	1 HYDROXYAKG + 1 GLT -> 1 L4HG + 1 AKG
177	Proline metabolism	1.5.1.12	1-pyrroline-5-carboxylate dehydrogenase	IRR	SC1C2.01	1 P35C + 1 NAD + 2 H2O -> 1 NADH + 1 E4HG + 1 H
178	Proline metabolism	1.5.1.12	1-pyrroline-5-carboxylate dehydrogenase	IRR	SC1C2.01	1 P35C + 1 NADP + 2 H2O -> 1 NADPH + 1 E4HG + 1 H
179	Proline metabolism	1.5.1.12	1-pyrroline-5-carboxylate dehydrogenase	IRR	SC1C2.01	1 NADH + 1 E4HG + 1 H -> 1 L4HGSA + 1 NAD + 1 H2O
180	Proline metabolism	1.5.1.12	1-pyrroline-5-carboxylate dehydrogenase	IRR	SC1C2.01	1 P5C + 1 NAD + 2 H2O -> 1 NADH + 1 GLT + 1 H
181	Proline metabolism	1.5.1.12	1-pyrroline-5-carboxylate dehydrogenase	IRR	SC1C2.01	1 GLUGSAL + 1 NAD + 1 H2O -> 1 NADH + 1 GLT + 1 H
182	Asparagine metabolism	3.5.1.1	Asparaginase	IRR		1 ASN + 1 H2O -> 1 NH3 + 1 ASP
183	Aspartate metabolism	4.1.1.11	Aspartate 1-decarboxylase	IRR	panD	1 ASP -> 1 BALA + 1 CO2
184	Aspartate metabolism	1.4.3.16	L-aspartate oxidase	IRR	nadB	1 ASP + 1 O2 + 1 H2O -> 1 OA + 1 NH3 + 1 H2O2
185	Lysine metabolism	4.1.1.18	lysine decarboxylase	IRR		1 LYS -> 1 CADA + 1 CO2
186	Lysine metabolism	add1	cadaverine aminotransferase	IRR		1 CADA + 1 AKG -> 1 PIPER + 1 GLT + 1 H2O
187	Lysine metabolism	add2	piperideine dehydrogenase	IRR		1 PIPER + 1 NAD + 2 H2O -> 1 AMINOVAL + 1 NADH + 1 H
188	Lysine	2.6.1.48	5-aminovalerate transaminase	IRR		1 AMINOVAL + 1 AKG -> 1 GLUTSEMI + 1 GLT

	metabolism					
189	Lysine metabolism	1.2.1.20	glutarate-semialdehyde dehydrogenase	IRR		1 GLUTSEMI + 1 NAD + 1 H2O -> 1 GLUT + 1 NADH + 1 H
190	Lysine metabolism	6.2.1.6	glutarate:CoA ligase (ADP-forming)	IRR		1 GLUT + 1 ATP + 1 COA -> 1 GLUTCOA + 1 ADP + 1 PI
191	Lysine metabolism	1.3.99.7	glutaryl-CoA dehydrogenase	IRR		1 GLUTCOA + 2 NAD -> 1 CROTCOA + 2 NADH + 1 CO2
192	Lysine metabolism (Propanoate metabolism)	4.2.1.17	Enoyl-CoA hydratase	REV		1 CROTCOA + 1 H2O -> 1 H3BUTCOA
193	Lysine metabolism (Butanoate metabolism)	1.1.1.35	3-hydroxybutyryl-CoA dehydrogenase	REV		1 NAD + 1 H3BUTCOA -> 1 NADH + 1 AACCOA + 1 H
194	Lysine metabolism (Butanoate metabolism)	1.1.1.157	3-hydroxybutyryl-CoA dehydrogenase	REV		1 NADP + 1 H3BUTCOA -> 1 NADPH + 1 AACCOA + 1 H
195	Lysine metabolism (Butanoate metabolism)	5.1.2.3	3-hydroxybutyryl-CoA epimerase	REV		1 H3BUTCOA -> 1 RH3BUTCOA
196	Lysine metabolism (Butanoate	1.1.1.30	3-hydroxybutyrate dehydrogenase	REV		1 RH3BUT + 1 NAD -> 1 ACTAC + 1 NADH + 1 H

	metabolism)					
197	Lysine metabolism (Leucine metabolism)	6.2.1.16	Acetoacetate--CoA ligase	IRR	acsA	1 COA + 1 ACTAC + 1 ATP -> 1 AACCOA + 1 PPI + 1 AMP
198	Lysine metabolism (Leucine metabolism)	2.8.3.5	3-oxoacid CoA-transferase scoA, scoB	REV		1 SUCCOA + 1 ACTAC -> 1 SUCC + 1 AACCOA
199	Methionine metabolism	2.5.1.6	Methionine adenosyltransferase	IRR	metK	1 ATP + 1 MET + 1 H2O -> 1 PI + 1 PPI + 1 SAMET
200	Methionine metabolism	3.3.1.1	Adenosylhomocysteinase	REV	sahH	1 SAH + 1 H2O -> 1 HOMOCYC + 1 ADN
201	Methionine metabolism	2.1.1.37	SAM-dependent methyltransferase	IRR		1 SAMET + 1 CYTOSINE -> 1 SAH + 1 M5CSN
202	Methionine metabolism	3.5.4.1	5-Methylcytosine aminohydrolase	IRR		1 M5CSN + 1 H2O -> 1 NH3 + 1 THY
203	Methionine metabolism	2.5.1.16	Spermidine synthase	IRR		1 SPERMINE + 1 MTADENOSINE + 1 H -> 1 SPERMIDINE + 1 SAMETA
204	Methionine metabolism	2.4.2.28	methylthio-D-ribosyltransferase	IRR		1 PI + 1 MTADENOSINE -> 1 AD + 1 MTRP
205	Methionine metabolism	2.5.1.16	Spermidine synthase	IRR		1 MTRP -> 1 MTRIP
206	Threonine metabolism	1.1.1.103	L-threonine 3-dehydrogenase	IRR	tdh	1 THR + 1 NAD -> 1 AOXOBUT + 1 NADH + 1 H

207	Threonine metabolism	spnts5	spontaneous	IRR		1 AOXOBUT -> 1 AACETONE + 1 CO2
208	Threonine metabolism	2.3.1.29	Glycine C-acetyltransferase	IRR	kbl	1 AOXOBUT + 1 COA -> 1 GLY + 1 ACCOA
209	Threonine metabolism	1.1.1.75	(R)-aminopropanol dehydrogenase	IRR		1 AACETONE + 1 NADH + 1 H -> 1 APROPANOL + 1 NAD
210	Glycine metabolism	1.8.1.4	Dihydrolipoamide dehydrogenase	IRR		1 DDLGCVH + 1 NAD -> 1 NADH + 1 PLIPOYLLYSINE + 1 H
211	Glycine metabolism	1.4.4.2	Glycine dehydrogenase (decarboxylating)	IRR		1 PLIPOYLLYSINE + 1 GLY -> 1 SAP + 1 CO2
212	Glycine metabolism	2.1.2.10	Aminomethyltransferase	IRR	gcvT	1 SAP + 1 THF -> 1 DDLGCVH + 1 METHTHF + 1 NH3
213	Glycine metabolism	2.1.2.10	Aminomethyltransferase	IRR	gcvT	1 GLY + 1 THF + 1 NAD -> 1 CO2 + 1 METHTHF + 1 NH3 + 1 NADH + 1 H
214	Glycine metabolism	1.8.1.4	Dihydrolipoamide dehydrogenase	REV		1 DLIPO + 1 NAD -> 1 LIPO + 1 NADH + 2 H
215	Serine metabolism	4.3.1.17	L-serine ammonia-lyase	IRR	sdaA	1 SER -> 1 PYR + 1 NH3
216	Cystine metabolism	4.4.1.1	Cystathionine gamma-lyase	IRR	cysA	1 CYS + 1 H2O -> 1 H2S + 1 PYR + 1 NH3
217	Valine metabolism	1.4.1.8	valine dehydrogenase	IRR		1 VAL + 1 NAD -> 1 NADH + 1 NH3 + 1 KISOVALERATE + 1 H
218	Valine metabolism	1.2.4.4	3-methyl-2-oxobutanoate dehydrogenase	IRR		1 KISOVALERATE + 1 LIPO -> 1 IBUTLIPO + 1 CO2
219	Valine metabolism	2.3.1.12	dihydrolipoyllysine-residue acetyltransferase	IRR		1 IBUTLIPO + 1 COA -> 1 ISOBUTCOA + 1 DLIPO
220	Valine	1.3.99.3	Butyryl-CoA dehydrogenase	IRR		1 ISOBUTCOA + 1 MK -> 1 MCCOA + 1 MKH2

	metabolism					
221	Valine metabolism	4.2.1.17	Enoyl-CoA hydratase	IRR		1 MCCOA + 1 H2O -> 1 HISOBUTCOA
222	Valine metabolism	3.1.2.4	3-hydroxyisobutyryl-CoA hydrolase	IRR	add	1 HISOBUTCOA + 1 H2O -> 1 HISOBUT + 1 COA
223	Valine metabolism	1.1.1.35/1.1.1.31	3-hydroxyacyl-CoA dehydrogenase	IRR		1 NAD + 1 HISOBUT -> 1 NADH + 1 MMALONATEALD + 1 H
224	Valine metabolism	1.2.1.27	Methylmalonate-semialdehyde dehydrogenase (acylating)	IRR	msdA	1 NAD + 1 COA + 1 MMALONATEALD -> 1 NADH + 1 CO2 + 1 PROPIONYLCOA + 1 H
225	Valine metabolism	1.2.1.3	Aldehyde dehydrogenase (NAD+)	IRR	thcA	1 MMALONATEALD + 1 NAD + 1 H2O -> 1 MMAL + 1 NADH + 1 H
226	Valine metabolism	1.2.1.3	Aldehyde dehydrogenase (NAD+)	IRR		1 MMAL + 1 COA -> 1 MMCOA + 1 H2O
227	Isolecine metabolism	1.4.1.8	valine dehydrogenase	IRR		1 ILE + 1 NAD -> 1 NADH + 1 NH3 + 1 KMVALERATE
228	Isolecine metabolism	1.2.4.4	3-methyl-2-oxobutanoate dehydrogenase	IRR		1 KMVALERATE + 1 LIPO -> 1 2MBUTLIPO + 1 CO2
229	Isolecine metabolism	2.3.1.12	dihydrolipoyllysine-residue acetyltransferase	IRR		1 2MBUTLIPO + 1 COA -> 1 2MBCOA + 1 DLIPO
230	Isolecine metabolism	1.3.99.3	Butyryl-CoA dehydrogenase	IRR		1 2MBCOA + 1 MK -> 1 TIGCOA + 1 MKH2
231	Isolecine metabolism	4.2.1.17	Enoyl-CoA hydratase	IRR		1 TIGCOA + 1 H2O -> 1 MHBUTCOA
232	Isolecine metabolism	1.1.1.35	3-hydroxyacyl-CoA dehydrogenase	IRR		1 NAD + 1 MHBUTCOA -> 1 NADH + 1 MAACOA + 1 H
233	Isolecine	2.3.1.16	Acetyl-CoA C-acyltransferase	IRR		1 COA + 1 MAACOA -> 1 PROPIONYLCOA + 1 ACCOA

	metabolism					
234	Isolecine metabolism	6.4.1.3	Propionyl-CoA carboxylase	IRR	pccB	1 PROPIONYLCOA + 1 ATP + 1 HCO3 -> 1 SMMCOA + 1 PI + 1 ADP
235	Isolecine metabolism	5.1.99.1	methylmalonyl-CoA epimerase	REV		1 SMMCOA -> 1 MMCOA
236	Isolecine metabolism	5.4.99.2	Methylmalonyl-CoA mutase	IRR	(icmB OR mutA2 OR icmA OR SCO6832 OR mutA)	1 MMCOA -> 1 SUCCOA
237	Leucine metabolism	1.4.1.8	valine dehydrogenase	IRR		1 LEU + 1 NAD -> 1 NADH +1 NH3 + 1 MPENTANOATE
238	Leucine metabolism	1.2.4.4	3-methyl-2-oxobutanoate dehydrogenase	IRR		1 MPENTANOATE + 1 LIPO -> 1 3MBUTLIPO + 1 CO2
239	Leucine metabolism	2.3.1.12	dihydrolipoyllysine-residue acetyltransferase	IRR		1 3MBUTLIPO + 1 COA -> 1 3MBCOA + 1 DLIPO
240	Leucine metabolism	1.3.99.3	Butyryl-CoA dehydrogenase	IRR		1 3MBCOA + 1 MK -> 1 MCROTCOA + 1 MKH2
241	Leucine metabolism	4.2.1.17	Enoyl-CoA hydratase	IRR		1 MCROTCOA + 1 H2O -> 1 ISOVALCOA
242	Leucine metabolism	6.4.1.4	Methylcrotonyl-CoA carboxylase	IRR		1 HCO3 + 1 MCROTCOA + 1 ATP -> 1 PI + 1 T3METGLUCOA + 1 ADP
243	Leucine metabolism	4.2.1.18	putative enoyl-CoA hydratase	IRR		1 T3METGLUCOA + 1 H2O -> 1 HMGCOA
244	Leucine metabolism	4.1.3.4	Hydroxymethylglutaryl-CoA lyase	IRR	hmgL	1 HMGCOA -> 1 ACTAC + 1 ACCOA
245	Histidine	4.3.1.3	Histidine ammonia-lyase	IRR	hutH	1 HIS -> 1 NH3 + 1 UROCANATE

	metabolism					
246	Histidine metabolism	4.2.1.49	Urocanate hydratase	IRR	hutU	1 UROCANATE + 1 H2O -> 1 IMI5PROP
247	Histidine metabolism	3.5.2.7	Imidazolonepropionase	IRR		1 IMI5PROP + 1 H2O -> 1 NFORMIGLU
248	Histidine metabolism	3.5.3.8	formimidoylglutamase	IRR		1 NFORMIGLU + 1 H2O -> 1 GLT + 1 FORMAMIDE
249	Tryptophan metabolism	1.13.11.11	tryptophan 2,3-dioxygenase	IRR		1 TRP + 1 O2 -> 1 FKYN
250	Tryptophan metabolism	3.5.1.9	N-Formylkynurenine amidohydrolase	IRR		1 FKYN + 1 H2O -> 1 FOR + 1 KYN
251	Tryptophan metabolism	3.7.1.3	L-Kynurenine hydrolase	IRR		1 KYN + 1 H2O -> 1 ALA + 1 AN
252	Tryptophan metabolism	3.7.1.3	Formylkynurenine hydrolase	IRR		1 FKYN + 1 H2O -> 1 FAN + 1 ALA
253	Tryptophan metabolism	3.5.1.9	N-Formylanthranilate amidohydrolase	IRR		1 FAN + 1 H2O -> 1 FOR + 1 AN
254	Tyrosine metabolism	2.6.1.9	Tyrosine aminotransferase	REV		1 TYR + 1 AKG -> 1 GLT + 1 PHPPYR
255	Tyrosine metabolism	1.13.11.27	4-hydroxyphenylpyruvate dioxygenase	IRR		1 PHPPYR + 1 O2 -> 1 CO2 + 1 HOMOGENTISATE
256	Tyrosine metabolism	1.13.11.5	Homogentisate 1,2-dioxygenase	IRR	hgd	1 O2 + 1 HOMOGENTISATE -> 1 MACETOACETATE
257	Tyrosine metabolism	5.2.1.2	maleylacetoacetate isomerase	REV		1 MACETOACETATE -> 1 FACETOACETATE
258	Tyrosine	3.7.1.2	Fumarylacetoacetase	IRR		1 FACETOACETATE + 1 H2O -> 1 FUM + 1 ACTAC

	metabolism					
259	Phenylalanine metabolism	2.6.1.9	aspartate transaminase	REV		1 PHE + 1 AKG -> 1 PHPYR + 1 GLT
260	Phenylalanine metabolism	4.1.1.43	phenylpyruvate decarboxylase	IRR		1 PHPYR -> 1 PHAC + 1 CO2
261	Phenylalanine metabolism	1.2.1.39	phenylpyruvate decarboxylase	IRR		1 PHAC + 1 NAD + 1 H2O -> 1 PAC + 1 NADH + 1 H
262	Alanine metabolism	1.4.1.1	alanine dehydrogenase	REV		1 ALA + 1 NAD + 1 H2O -> 1 PYR + 1 NH3 + 1 NADH + 1 H
263	Alanine metabolism	2.6.1.2	aminotransferase AlaT	REV	alaT	1 ALA + 1 AKG -> 1 PYR + 1 GLT
264	Pyrimidines biosynthesis	6.3.5.5	carbamoylphosphate synthetase large chain/carbamoyl-phosphate synthase, pyrimidine-specific, small chain	IRR	(pyrA,pyrAA)	1 HCO3 + 1 GLN + 2 ATP + 1 H + 1 H2O -> 1 CAP + 1 GLT + 1 PI + 2 ADP
265	Pyrimidines biosynthesis	2.1.3.2	Aspartate carbamoyltransferase	IRR	pyrB	1 ASP + 1 CAP -> 1 CAASP + 1 PI
266	Pyrimidines biosynthesis	3.5.2.3	Dihydroorotase	REV	pyrC	1 CAASP -> 1 DOROA + 1 H2O
267	Pyrimidines biosynthesis	1.3.3.1	Dihydroorotate oxidase	IRR	pyrD	1 MK + 1 DOROA -> 1 MKH2 + 1 OROA
268	Pyrimidines biosynthesis	1.3.3.1	DihydroOROA oxidase	IRR	pyrD	1 O2 + 1 DOROA -> 1 H2O2 + 1 OROA
269	Pyrimidines biosynthesis	2.4.2.10	Orotate phosphoribosyltransferase	REV	pyrE	1 PRPP + 1 OROA -> 1 OMP + 1 PPI
270	Pyrimidines biosynthesis	4.1.1.23	Orotidine-5'-phosphate decarboxylase	IRR	pyrF	1 OMP -> 1 CO2 + 1 UMP

271	Pyrimidines biosynthesis	2.7.4.14	cytidylate kinase	REV		1 ATP + 1 UMP -> 1 ADP + 1 UDP
272	Pyrimidines biosynthesis	2.7.1.48	ADDED	IRR		1 GTP + 1 URI -> 1 GDP + 1 UMP
273	Pyrimidines biosynthesis	3.1.3.5	Adenosine kinase	IRR		1 UMP + 1 H2O -> 1 URI + 1 PI
274	Pyrimidines biosynthesis	2.4.2.3	Uridine phosphorylase	REV		1 PI + 1 URI -> 1 R1P + 1 URA
275	Pyrimidines biosynthesis	2.7.4.6	Nucleoside-diphosphate kinase	REV	ndk	1 UDP + 1 ATP -> 1 UTP + 1 ADP
276	Pyrimidines biosynthesis	6.3.4.2	CTP synthetase	IRR	pyrG	1 GLN + 1 UTP + 1 ATP + 1 H2O -> 1 CTP + 1 PI + 1 ADP + 1 GLT
277	Pyrimidines biosynthesis	6.3.4.2	CTP synthetase	IRR	pyrG	1 ATP + 1 UTP + 1 NH3 -> 1 ADP + 1 PI + 1 CTP
278	Pyrimidines biosynthesis	2.7.4.6	nucleoside-diphosphate kinase	REV	ndk	1 CTP + 1 ADP -> 1 CDP + 1 ATP
279	Pyrimidines biosynthesis	1.17.4.1	ribonucleoside-diphosphate reductase	IRR		1 RTHIO + 1 CDP -> 1 OTHIO + 1 DCDP + 1 H2O
280	Pyrimidines biosynthesis	3.1.3.5	Adenosine kinase	IRR		1 DCMP + 1 H2O -> 1 DC + 1 PI
281	Pyrimidines biosynthesis	2.7.4.6	nucleoside-diphosphate kinase	REV	ndk	1 DCDP + 1 ATP -> 1 DCTP + 1 ADP
282	Pyrimidines biosynthesis	2.7.1.21	thymidine kinase	IRR		1 DU + 1 ATP -> 1 DUMP + 1 ADP
283	Pyrimidines biosynthesis	3.5.4.13	dCTP deaminase	IRR		1 CTP + 1 H2O -> 1 NH3 + 1 UTP

284	Pyrimidines biosynthesis	3.5.4.13	dCTP deaminase	IRR		1 DCTP + 1 H2O -> 1 NH3 + 1 DUTP
285	Pyrimidines biosynthesis	3.6.1.19	ucleoside triphosphate pyrophosphohydrolase	IRR	dut	1 DTTP + 1 H2O -> 1 PPI + 1 DTMP
286	Pyrimidines biosynthesis	3.6.1.19	ucleoside triphosphate pyrophosphohydrolase	IRR	dut	1 DGTP + 1 H2O -> 1 PPI + 1 DGMP
287	Pyrimidines biosynthesis	3.6.1.19	ucleoside triphosphate pyrophosphohydrolase	IRR	dut	1 DCTP + 1 H2O -> 1 PPI + 1 DCMP
288	Pyrimidines biosynthesis	3.6.1.19	ucleoside triphosphate pyrophosphohydrolase	IRR	dut	1 CTP + 1 H2O -> 1 PPI + 1 CMP
289	Pyrimidines biosynthesis	3.6.1.19	ucleoside triphosphate pyrophosphohydrolase	IRR	dut	1 ATP + 1 H2O -> 1 PPI + 1 AMP
290	Pyrimidines biosynthesis	3.6.1.19	ucleoside triphosphate pyrophosphohydrolase	IRR	dut	1 GTP + 1 H2O -> 1 PPI + 1 GMP
291	Pyrimidines biosynthesis	3.6.1.19	ucleoside triphosphate pyrophosphohydrolase	IRR	dut	1 ITP + 1 H2O -> 1 PPI + 1 IMP
292	Pyrimidines biosynthesis	3.6.1.19	ucleoside triphosphate pyrophosphohydrolase	IRR	dut	1 UTP + 1 H2O -> 1 PPI + 1 UMP
293	Pyrimidines biosynthesis	3.6.1.23	dUTP pyrophosphatase	IRR	dut	1 DUTP + 1 H2O -> 1 PPI + 1 DUMP
294	Pyrimidines biosynthesis	2.7.4.6	Nucleoside-diphosphate kinase	REV	ndk	1 DTDP + 1 ATP -> 1 DTTP + 1 ADP
295	Pyrimidines biosynthesis	2.7.4.9	Thymidylate kinase	REV		1 DTMP + 1 ATP -> 1 DTDP + 1 ADP
296	Pyrimidines biosynthesis	2.1.1.148	thymidylate synthase	IRR		1 METHTHF + 1 DUMP + 1 FADH2 -> 1 THF + 1 DTMP + 1 FAD

297	Pyrimidines biosynthesis	2.7.4.9	Thymidylate kinase	REV		1 TMP + 1 ATP -> 1 TDP + 1 ADP
298	Pyrimidines biosynthesis	2.7.4.14	Cytidylate kinase	REV		1 DCMP + 1 ATP -> 1 DCDP + 1 ADP
299	Pyrimidines biosynthesis	2.7.4.14	Cytidylate kinase	REV		1 CMP + 1 ATP -> 1 CDP + 1 ADP
300	Pyrimidines biosynthesis	3.1.3.5	Adenosine kinase	IRR		1 DTMP + 1 H2O -> 1 DT + 1 PI
301	Pyrimidines biosynthesis	3.1.3.5	Adenosine kinase	IRR		1 CMP + 1 H2O -> 1 CYTD + 1 PI
302	Pyrimidines biosynthesis	3.5.4.5	Cytidine deaminase	IRR		1 DC + 1 H2O -> 1 NH3 + 1 DU
303	Pyrimidines biosynthesis	3.5.4.5	Cytidine deaminase	IRR		1 CYTD + 1 H2O -> 1 NH3 + 1 URI
304	Pyrimidines biosynthesis	2.7.1.21	Thymidine kinase	IRR		1 DT + 1 ATP -> 1 TMP + 1 ADP
305	Pyrimidines biosynthesis	2.4.2.4	Thymidine phosphorylase	REV	deoA	1 PI + 1 DT -> 1 DR1P + 1 THY
306	Pyrimidines biosynthesis	2.4.2.9	Uracil phosphoribosyltransferase	IRR		1 PRPP + 1 URA -> 1 PPI + 1 UMP
307	Purine biosynthesis	2.7.6.1	ribose-phosphate diphosphokinase	REV	prsA2	1 ATP + 1 R5P -> 1 PRPP + 1 AMP
308	Purine biosynthesis	2.4.2.14	Amidophosphoribosyltransferase	IRR	purF	1 GLN + 1 PRPP + 1 H2O -> 1 PRAM + 1 PPI + 1 GLT
309	Purine biosynthesis	6.3.4.13	Phosphoribosylamine--glycine ligase	REV	purD	1 PRAM + 1 ATP + 1 GLY -> 1 ADP + 1 PI + 1 GAR

310	Purine biosynthesis	2.1.2.2	Phosphoribosylglycinamide formyltransferase	REV	purN	1 F10THF + 1 GAR -> 1 THF + 1 FGAR
311	Purine biosynthesis	6.3.5.3	phosphoribosylformylglycinamidine synthetase	IRR	(purS,purQ,purL)	1 ATP + 1 FGAR + 1 GLN + 1 H2O -> 1 ADP + 1 PI + 1 FGAM + 1 GLT
312	Purine biosynthesis	6.3.3.1	phosphoribosylformylglycinamidine cyclo-ligase	IRR	purM	1 ATP + 1 FGAM -> 1 ADP + 1 PI + 1 AIR
313	Purine biosynthesis	4.1.1.21	Phosphoribosylaminoimidazole carboxylase	IRR	purE	1 AIR + 1 CO2 -> 1 CAIR
314	Purine biosynthesis	6.3.2.6	Phosphoribosylaminoimidazole-succinocarboxamide synthetase	REV	purC	1 ATP + 1 CAIR + 1 ASP -> 1 ADP + 1 PI + 1 SAICAIR
315	Purine biosynthesis	4.3.2.2	Adenylosuccinate lyase	REV	purB	1 SAICAIR -> 1 FUM + 1 AICAR
316	Purine biosynthesis	2.4.2.7	Adenine phosphoribosyltransferase	IRR	apt	1 PRPP + 1 AIC -> 1 AICAR + 1 PPI
317	Purine biosynthesis	2.1.2.3	Phosphoribosylaminoimidazolecarboxamide formyltransferase	REV	purH	1 F10THF + 1 AICAR -> 1 THF + 1 PRFICA
318	Purine biosynthesis	3.5.4.10	IMP cyclohydrolase	REV	purH	1 PRFICA -> 1 IMP + 1 H2O
319	Purine biosynthesis	2.7.1.20	Adenosine kinase	IRR		1 ADN + 1 ATP -> 1 AMP + 1 ADP
320	Purine biosynthesis	3.1.3.5	Adenosine kinase	IRR		1 AMP + 1 H2O -> 1 ADN + 1 PI
321	Purine biosynthesis	3.1.3.5	Xanthenosine kinase	IRR		1 XMP + 1 H2O -> 1 XNSN + 1 PI
322	Purine biosynthesis	3.1.3.5	Guanosine kinase	IRR		1 GMP + 1 H2O -> 1 GSN + 1 PI

323	Purine biosynthesis	3.1.3.5	Inosine kinase	IRR		1 IMP + 1 H2O -> 1 INS + 1 PI
324	Purine biosynthesis	3.1.3.5	Adenosine kinase	IRR		1 DAMP + 1 H2O -> 1 DA + 1 PI
325	Purine biosynthesis	3.1.3.5	Guanosine kinase	IRR		1 DGMP + 1 H2O -> 1 DG + 1 PI
326	Purine biosynthesis	2.4.2.1	Thymidine phosphorylase	REV	deoA	1 PI + 1 INS -> 1 R1P + 1 HYXN
327	Purine biosynthesis	2.4.2.1	Thymidine phosphorylase	REV	deoA	1 PI + 1 DIN -> 1 DR1P + 1 HYXN
328	Purine biosynthesis	2.4.2.1	Thymidine phosphorylase	REV	deoA	1 PI + 1 DA -> 1 DR1P + 1 AD
329	Purine biosynthesis	2.7.4.8	Guanylate kinase	REV		1 DGMP + 1 ATP -> 1 DGDP + 1 ADP
330	Purine biosynthesis	4.6.1.1	Adenylate cyclase	IRR		1 ATP -> 1 CAMP + 1 PPI
331	Purine biosynthesis	4.6.1.1	Adenylate cyclase	IRR		1 GTP -> 1 CGMP + 1 PPI
332	Purine biosynthesis	2.7.4.6	Nucleoside-diphosphate kinase	REV	ndk	1 IDP + 1 ATP -> 1 ITP + 1 ADP
333	Purine biosynthesis	2.7.4.6	Nucleoside-diphosphate kinase	REV	ndk	1 DUDP + 1 ATP -> 1 DUTP + 1 ADP
334	Purine biosynthesis	1.17.4.1	ribonucleoside-diphosphate reductase	IRR		1 RTHIO + 1 UDP -> 1 OTHIO + 1 DUDP + 1 H2O
335	Purine biosynthesis	2.4.2.8	Hypoxanthine phosphoribosyltransferase	IRR	hprT	1 PRPP + 1 AD -> 1 AMP + 1 PPI

336	Purine biosynthesis	2.4.2.8	Hypoxanthine phosphoribosyltransferase	IRR	hprT	1 PRPP + 1 HYXN -> 1 IMP + 1 PPI
337	Purine biosynthesis	2.4.2.8	Xanthine-guanine phosphoribosyltransferase	IRR	hprT	1 PRPP + 1 GN -> 1 GMP + 1 PPI
338	Purine biosynthesis	2.4.2.8	Xanthine-guanine phosphoribosyltransferase	IRR	hprT	1 XAN + 1 PRPP -> 1 XMP + 1 PPI
339	Purine biosynthesis	2.4.2.1	Purine-nucleoside phosphorylase	REV		1 GN + 1 DR1P -> 1 DG + 1 PI
340	Purine biosynthesis	2.4.2.1	putative purine nucleoside phosphorylase	REV		1 XNSN + 1 PI -> 1 R1P + 1 XAN
341	Purine biosynthesis	2.4.2.1	Purine-nucleoside phosphorylase	REV		1 GSN + 1 PI -> 1 R1P + 1 GN
342	Purine biosynthesis	2.4.2.1	Purine-nucleoside phosphorylase	REV		1 ADN + 1 PI -> 1 R1P + 1 AD
343	Purine biosynthesis	2.4.2.7	Adenine phosphoribosyltransferase	IRR	apt	1 PRPP + 1 AD -> 1 AMP + 1 PPI
344	Purine biosynthesis	3.5.4.4	Adenosine deaminase	IRR	add	1 ADN + 1 H2O -> 1 NH3 + 1 INS
345	Purine biosynthesis	3.5.4.4	Adenosine deaminase	IRR	add	1 DA + 1 H2O -> 1 NH3 + 1 DIN
346	Purine biosynthesis	2.4.2.4	Purine-nucleoside phosphorylase	REV		1 URA + 1 DR1P -> 1 DU + 1 PI
347	Purine biosynthesis	1.17.1.4	Xanthine dehydrogenase	IRR		1 NAD + 1 HYXN + 1 H2O -> 1 NADH + 1 XAN + 1 H
348	Purine biosynthesis	1.17.1.4	Xanthine dehydrogenase	IRR		1 NAD + 1 XAN + 1 H2O -> 1 NADH + 1 URATE + 1 H

349	Purine biosynthesis	1.7.3.3	urate oxidase	IRR		1 URATE + 1 O2 + 1 H2O -> 1 HIU + 1 CO2 + 1 H2O2
350	Purine biosynthesis	spnt	spontaneous	IRR		1 HIU + 1 H2O -> 1 ATN
351	Purine biosynthesis	3.5.2.5	Allantoinase	IRR		1 ATN + 1 H2O -> 1 ATT
352	Purine biosynthesis	3.5.3.4	Allantoicase	IRR		1 ATT + 1 H2O -> 1 UGC + 1 UREA
353	Purine biosynthesis	3.5.3.19	Ureidoglycolate hydrolase	IRR		1 UGC + 1 H2O -> 1 CO2 + 2 NH3 + 1 GLX
354	Purine biosynthesis	3.1.5.1	Deoxyguanosinetriphosphate triphosphohydrolase	IRR		1 GTP + 1 H2O -> 1 P3I + 1 GSN
355	Purine biosynthesis	3.1.5.1	Deoxyguanosinetriphosphate triphosphohydrolase	IRR		1 DGTP + 1 H2O -> 1 P3I + 1 DG
356	Purine biosynthesis	2.7.6.5	GTP diphosphokinase	IRR	rshA	1 GTP + 1 ATP -> 1 GDPTP + 1 AMP
357	Purine biosynthesis	3.6.1.40	pppGpp 5'-phosphohydrolase	IRR		1 GDPTP + 1 H2O -> 1 GU5DP3DP + 1 PI
358	Purine biosynthesis	3.1.7.2	Guanosine-3',5'-bis(diphosphate) 3'-pyrophosphohydrolase	IRR	rshA	1 GU5DP3DP + 1 H2O-> 1 PPI + 1 GDP
359	ATP, dATP biosynthesis	6.3.4.4	adenylosuccinate synthetase	IRR	purA	1 ASP + 1 IMP + 1 GTP -> 1 ADENYLOSUCC + 1 PI + 1 GDP
360	ATP, dATP biosynthesis	4.3.2.2	Adenylosuccinate lyase	REV	purB	1 ADENYLOSUCC -> 1 FUM + 1 AMP
361	ATP, dATP biosynthesis	2.7.4.3	Adenylate kinase	REV	adk	1 DAMP + 1 ATP -> 1 DADP + 1 ADP

362	ATP, dATP biosynthesis	2.7.4.3	Adenylate kinase	REV	adk	1 AMP + 1 ATP -> 2 ADP
363	ATP, dATP biosynthesis	1.17.4.1	ribonucleoside-diphosphate reductase	IRR	(nrdM,nrmL)	1 RTHIO + 1 ADP -> 1 OTHIO + 1 DADP + 1 H2O
364	ATP, dATP biosynthesis	2.7.4.6	nucleoside-diphosphate kinase	REV	ndk	1 DADP + 1 ATP -> 1 DATP + 1 ADP
365	GTP, dGTP biosynthesis	1.1.1.205	IMP dehydrogenase	IRR	(guaB,SCD63.03)	1 NAD + 1 IMP + 1 H2O -> 1 XMP + 1 NADH + 1 H
366	GTP, dGTP biosynthesis	6.3.5.2	GMP synthetase (glutamine-hydrolysing)	IRR	guaA	1 XMP + 1 GLN + 1 ATP + 1 H2O -> 1 GMP + 1 PPI + 1 AMP + 1 GLT
367	GTP, dGTP biosynthesis	2.7.4.8	Guanylate kinase	REV		1 GMP + 1 ATP -> 1 GDP + 1 ADP
368	GTP, dGTP biosynthesis	2.7.4.6	nucleoside-diphosphate kinase	REV	ndk	1 GDP + 1 ATP -> 1 GTP + 1 ADP
369	GTP, dGTP biosynthesis	1.17.4.1	ribonucleoside-diphosphate reductase	IRR	(nrdM,nrmL)	1 RTHIO + 1 GDP -> 1 OTHIO + 1 DGDP + 1 H2O
370	GTP, dGTP biosynthesis	2.7.4.6	nucleoside-diphosphate kinase	REV	ndk	1 DGDP + 1 ATP -> 1 DGTP + 1 ADP
371	Fatty acid biosysnthesis	2.3.1.39	[acyl-carrier-protein] S-malonyltransferase	REV		1 MALCOA + 1 ACP -> 1 MALACP + 1 COA
372	Fatty acid biosysnthesis	2.3.1.41	3-oxoacyl-[acyl-carrier-protein] synthase	IRR		1 ACCOA + 1 ACP -> 1 ACACP + 1 COA
373	Fatty acid biosysnthesis	2.3.1.41/1.1.1.100/1.3.1.9	Tetradecanoyl-[acyl-carrier protein] synthesis (lumped reaction)	IRR		1 ACACP + 6 MALACP + 12 NADPH -> 12 NADP + 1 C140ACP + 6 CO2 + 6 ACP
374	Fatty acid biosysnthesis	2.3.1.41/1.1.1.100/1.3.1.9	Hexadecanoyl-[acyl-carrier protein] synthesis (lumped reaction)	IRR		1 ACACP + 7 MALACP + 14 NADPH -> 14 NADP + 1 C160ACP + 7 CO2 + 7 ACP

375	Fatty acid biosysnthesis	2.3.1.41/1.1.1.100/1.3.1.9	Oleoyl-[acyl-carrier protein] synthesis (lumped reaction)	IRR		1 ACACP + 8 MALACP + 15 NADPH -> 15 NADP + 1 C181ACP + 8 CO2 + 8 ACP
376	Fatty acid biosysnthesis	2.3.1.41/1.1.1.100/1.3.1.9	[acyl-carrier-protein] S-malonyltransferase	IRR		1 PROPIONYLCOA + 1 ACP -> 1 PROPIONYLACP + 1 COA
377	Fatty acid biosysnthesis	2.3.1.41/1.1.1.100/1.3.1.9	Pentadecanoyl-[acyl-carrier protein] synthesis (lumped reaction)	IRR		1 PROPIONYLACP + 6 MALACP + 12 NADPH -> 12 NADP + 1 C150ACP + 6 CO2 + 6 ACP
378	Fatty acid biosysnthesis	2.3.1.41/1.1.1.100/1.3.1.9	Heptadecanoyl-[acyl-carrier protein] synthesis (lumped reaction)	IRR		1 PROPIONYLACP + 7 MALACP + 14 NADPH -> 14 NADP + 1 C170ACP + 7 CO2 + 7 ACP
379	Fatty acid biosysnthesis	2.3.1.9	acetoacetyl-CoA thiolase	REV		2 ACCOA -> 1 COA + 1 AACCOA
380	Fatty acid biosysnthesis	1.1.1.35/4.2.1.17	butanoyl-CoA synthesis (lumped reaction)	REV		1 AACCOA + 2 NADH -> 2 NAD + 1 C040COA
381	Fatty acid biosysnthesis	1.1.1.35/4.2.1.17/5.1.2.3	hexanoyl-CoA synthesis (lumped reaction)	REV		1 AACCOA + 1 ACCOA + 4 NADH -> 4 NAD + 1 C060COA + 1 COA
382	Fatty acid biosysnthesis	1.1.1.35/4.2.1.17/5.1.2.3	octanoyl-CoA synthesis (lumped reaction)	REV		1 AACCOA + 2 ACCOA + 6 NADH -> 6 NAD + 1 C080COA + 2 COA
383	Fatty acid biosysnthesis	1.1.1.35/4.2.1.17/5.1.2.3	decanoyl-CoA synthesis (lumped reaction)	REV		1 AACCOA + 3 ACCOA + 8 NADH -> 8 NAD + 1 C100COA + 3 COA
384	Fatty acid biosysnthesis	1.1.1.35/4.2.1.17/5.1.2.3	dodecanoyl-CoA synthesis (lumped reaction)	REV		1 AACCOA + 4 ACCOA + 10 NADH -> 10 NAD + 1 C120COA + 4 COA
385	Fatty acid biosysnthesis	1.1.1.35/4.2.1.17/5.1.2.3	tetradecanoyl-CoA synthesis (lumped reaction)	REV		1 AACCOA + 5 ACCOA + 12 NADH -> 12 NAD + 1 C140COA + 5 COA
386	Fatty acid biosysnthesis	1.1.1.35/4.2.1.17/5.1.2.3	hexadecanoyl-CoA synthesis (lumped reaction)	REV		1 AACCOA + 6 ACCOA + 14 NADH -> 14 NAD + 1 C160COA + 6 COA
387	Fatty acid biosysnthesis	6.2.1.3	acylCoA synthases	IRR		1 HEXANOATE + 1 COA + 1 ATP -> 1 C060COA + 1 AMP + 1 PPI

388	Fatty acid biosysnthesis	6.2.1.3	acylCoA synthases	IRR		1 OCTANOATE + 1 COA + 1 ATP -> 1 C080COA + 1 AMP + 1 PPI
389	Fatty acid biosysnthesis	6.2.1.3	acylCoA synthases	IRR		1 DECANOATE + 1 COA + 1 ATP -> 1 C100COA + 1 AMP + 1 PPI
390	Fatty acid biosysnthesis	6.2.1.3	acylCoA synthases	IRR		1 DODECANOATE + 1 COA + 1 ATP -> 1 C120COA + 1 AMP + 1 PPI
391	Fatty acid biosysnthesis	6.2.1.3	acylCoA synthases	IRR		1 TETRADECANOATE + 1 COA + 1 ATP -> 1 C140COA + 1 AMP + 1 PPI
392	Fatty acid biosysnthesis	6.2.1.3	acylCoA synthases	IRR		1 HEXADECANOATE + 1 COA + 1 ATP -> 1 C160COA + 1 AMP + 1 PPI
393	Fatty acid biosysnthesis	6.2.1.3	acylCoA synthases	IRR		1 HEPTADECANOATE + 1 COA + 1 ATP -> 1 C160COA + 1 AMP + 1 PPI
394	Fatty acid metabolism	1.3.99.3 AND 4.2.1.17 AND 1.1.1.35 AND 2.3.1.16	acyl-CoA dehydrogenase fadE1-36 AND enoyl-CoA hydratase/isomerase echA1-21 AND 3-hydroxyacyl-CoA dehydrogenase fadB2-5 AND acetyl-CoA C-acetyltransferase fadA2-12	IRR		1 C170COA + 1 FAD + 1 NAD + COA -> 1 C160COA + 1 FADH2 + 1 NADH + 1 AACCOA
395	Fatty acid metabolism	1.3.99.3 AND 4.2.1.17 AND 1.1.1.35 AND 2.3.1.16	acyl-CoA dehydrogenase fadE1-36 AND enoyl-CoA hydratase/isomerase echA1-21 AND 3-hydroxyacyl-CoA dehydrogenase fadB2-5 AND acetyl-CoA C-acetyltransferase fadA2-12	IRR		1 C160COA + 1 FAD + 1 NAD + COA -> 1 C140COA + 1 FADH2 + 1 NADH + 1 AACCOA
396	Fatty acid metabolism	1.3.99.3 AND 4.2.1.17 AND 1.1.1.35 AND 2.3.1.16	acyl-CoA dehydrogenase fadE1-36 AND enoyl-CoA hydratase/isomerase echA1-21 AND 3-hydroxyacyl-CoA dehydrogenase fadB2-5 AND acetyl-CoA	IRR		1 C140COA + 1 FAD + 1 NAD + COA -> 1 C120COA + 1 FADH2 + 1 NADH + 1 AACCOA

			C-acetyltransferase fadA2-13			
397	Fatty acid metabolism	1.3.99.3 AND 4.2.1.17 AND 1.1.1.35 AND 2.3.1.16	acyl-CoA dehydrogenase fadE1-36 AND enoyl-CoA hydratase/isomerase echA1-21 AND 3-hydroxyacyl-CoA dehydrogenase fadB2-5 AND acetyl-CoA C-acetyltransferase fadA2-14	IRR		1 C120COA + 1 FAD + 1 NAD + COA -> 1 C100COA + 1 FADH2 + 1 NADH + 1 AACCOA
398	Fatty acid metabolism	1.3.99.3 AND 4.2.1.17 AND 1.1.1.35 AND 2.3.1.16	acyl-CoA dehydrogenase fadE1-36 AND enoyl-CoA hydratase/isomerase echA1-21 AND 3-hydroxyacyl-CoA dehydrogenase fadB2-5 AND acetyl-CoA C-acetyltransferase fadA2-15	IRR		1 C100COA + 1 FAD + 1 NAD + COA -> 1 C080COA + 1 FADH2 + 1 NADH + 1 AACCOA
399	Fatty acid metabolism	1.3.99.3 AND 4.2.1.17 AND 1.1.1.35 AND 2.3.1.16	acyl-CoA dehydrogenase fadE1-36 AND enoyl-CoA hydratase/isomerase echA1-21 AND 3-hydroxyacyl-CoA dehydrogenase fadB2-5 AND acetyl-CoA C-acetyltransferase fadA2-16	IRR		1 C080COA + 1 FAD + 1 NAD + COA -> 1 C060COA + 1 FADH2 + 1 NADH + 1 AACCOA
400	Fatty acid metabolism	1.3.99.3 AND 4.2.1.17 AND 1.1.1.35 AND 2.3.1.16	acyl-CoA dehydrogenase fadE1-36 AND enoyl-CoA hydratase/isomerase echA1-21 AND 3-hydroxyacyl-CoA dehydrogenase fadB2-5 AND acetyl-CoA C-acetyltransferase fadA2-17	IRR		1 C060COA + 1 FAD + 1 NAD + COA -> 1 C040COA + 1 FADH2 + 1 NADH + 1 AACCOA

401	Fatty acid metabolism	1.3.99.3 AND 4.2.1.17 AND 1.1.1.35 AND 2.3.1.16	acyl-CoA dehydrogenase fadE1-36 AND enoyl-CoA hydratase/isomerase echA1-21 AND 3-hydroxyacyl-CoA dehydrogenase fadB2-5 AND acetyl-CoA C-acetyltransferase fadA2-18	IRR		1 C040COA + 1 FAD + 1 NAD + COA -> 1 FADH2 + 1 NADH + 2 AACCOA
402	Phosphatidylglycerol biosynthesis	1.1.1.94	Glycerol-3-phosphate dehydrogenase (NAD(P)+)	REV	gpsA	1 DHAP + 1 NADPH + 1 H -> 1 GL3P + 1 NADP
403	Phosphatidylglycerol biosynthesis	2.3.1.15	glycerol-3-phosphate O-acyltransferase	IRR		1 GL3P + 0.094 C140ACP + 0.294 C150ACP + 0.262 C160ACP + 0.293 C170ACP + 0.057 C181ACP -> 1 AGL3P + 1 ACP
404	Phosphatidylglycerol biosynthesis	2.3.1.42	glycerone-phosphate O-acyltransferase	IRR		1 DHAP + 0.094 C140ACP + 0.294 C150ACP + 0.262 C160ACP + 0.293 C170ACP + 0.057 C181ACP -> 1 ADHAP + 1 ACP
405	Phosphatidylglycerol biosynthesis	1.1.1.101	acylglycerone-phosphate reductase	IRR		ADHAP + NADPH + 1 H -> AGL3P + NADP
406	Phosphatidylglycerol biosynthesis	2.3.1.51	1-acylglycerol-3-phosphate O-acyltransferase	IRR		1 AGL3P + 0.094 C140ACP + 0.294 C150ACP + 0.262 C160ACP + 0.293 C170ACP + 0.057 C181ACP -> 1 PHOSPHATIDATE + 1 ACP
407	Phosphatidylglycerol biosynthesis	2.7.7.41	phosphatidate cytidylyltransferase	REV		1 CTP + 1 PHOSPHATIDATE -> 1 CDPDIACYLGLYCEROL + 1 PPI
408	Phosphatidylglycerol biosynthesis	2.7.8.8	CDP-diacylglycerol--serine O-phosphatidyltransferase	REV		1 CDPDIACYLGLYCEROL + 1 SER -> 1 CMP + 1 PSER
409	Phosphatidylglycerol biosynthesis	4.1.1.65	Phosphatidylserine decarboxylase	IRR		1 PSER -> 1 PE + 1 CO2
410	Phosphatidylglycerol biosynthesis	2.7.8.5	CDP-diacylglycerol--glycerol-3-phosphate 3-phosphatidyltransferase	REV		1 CDPDIACYLGLYCEROL + 1 GL3P -> 1 PGP + 1 CMP
411	Phosphatidylglycerol biosynthesis	3.1.3.27	Phosphatidylglycerophosphatase	IRR		1 PGP + 1 H2O -> 1 PG + 1 PI
412	Phosphatidylglyce	2.7.8.-	cardiolipin synthase	IRR		2 PG -> 1 GL + 1 CDL

	rol biosynthesis					
413	Phosphatidylglycerol biosynthesis	2.7.7.39	glycerol-3-phosphate cytidylyltransferase	IRR		GL3P + CTP -> PPI + CDPGL
414	Phosphatidylglycerol biosynthesis	2.7.8.12	CDP-glycerol glycerophosphotransferase	IRR		12 CDPGL + 1 POLYGP -> 12 CMP + 1 POLYGP
415	Polyprenoids, Steroid, Phytoene biosynthesis	2.2.1.7	1-deoxy-D-xylulose-5-phosphate synthase	IRR		1 PYR + 1 GAP -> 1 DX5P + 1 CO2
416	Polyprenoids, Steroid, Phytoene biosynthesis	1.1.1.267	1-deoxy-D-xylulose-5-phosphate reductoisomerase	IRR	dxr	1 DX5P + 1 NADPH + 1 H -> 1 MDE4P + 1 NADP
417	Polyprenoids, Steroid, Phytoene biosynthesis	2.7.7.60	2-C-methyl-D-erythritol 4-phosphate cytidylyltransferase	IRR		1 MDE4P + 1 CTP -> 1 CDPMDE + 1 PPI
418	Polyprenoids, Steroid, Phytoene biosynthesis	2.7.1.148	4-(cytidine 5'-diphospho)-2-C-methyl-D-erythritol kinase	IRR		1 CDPMDE + 1 ATP -> 1 2PCDPMDE + 1 ADP
419	Polyprenoids, Steroid, Phytoene biosynthesis	4.6.1.12	2-C-methyl-D-erythritol 2,4-cyclodiphosphate synthase	IRR		1 2PCDPMDE -> 1 MDECPP + 1 CMP
420	Polyprenoids, Steroid, Phytoene biosynthesis	1.17.4.3	4-hydroxy-3-methylbut-2-en-1-yl diphosphate synthase	IRR	gcpE	1 MDECPP + 1 NADH + 1 H -> 1 HMB4PP + 1 NAD + 1 H2O
421	Polyprenoids, Steroid, Phytoene biosynthesis	1.17.1.2	4-hydroxy-3-methylbut-2-enyl diphosphate reductase	IRR	lytB	1 HMB4PP + 1 NADPH + 1 H -> 1 IPP + 1 NADP + 1 H2O

422	Polyprenoids, Steroid, Phytoene biosynthesis	1.17.1.2	4-hydroxy-3-methylbut-2-enyl diphosphate reductase	IRR	lytB	1 HMB4PP + 1 NADH + 1 H -> 1 IPP + 1 NAD + 1 H2O
423	Polyprenoids, Steroid, Phytoene biosynthesis	1.17.1.2	4-hydroxy-3-methylbut-2-enyl diphosphate reductase	IRR	lytB	1 HMB4PP + 1 NADH + 1 H -> 1 DMPP + 1 NAD + 1 H2O
424	Polyprenoids, Steroid, Phytoene biosynthesis	1.17.1.2	4-hydroxy-3-methylbut-2-enyl diphosphate reductase	IRR	lytB	1 HMB4PP + 1 NADPH + 1 H -> 1 DMPP + 1 NADP + 1 H2O
425	Polyprenoids, Steroid, Phytoene biosynthesis	5.3.3.2	Isopentenyl-diphosphate delta-isomerase	IRR		1 IPP -> 1 DMPP
426	Polyprenoids, Steroid, Phytoene biosynthesis	2.5.1.1	Dimethylallyltransferase	IRR	gtr	1 DMPP + 1 IPP -> 1 PPI + 1 GPP
427	Polyprenoids, Steroid, Phytoene biosynthesis	2.5.1.10	geranyltranstransferase	IRR	gtr	1 GPP + 1 IPP -> 1 PPI + 1 FPP
428	Polyprenoids, Steroid, Phytoene biosynthesis	2.5.1.29	farnesyltranstransferase	IRR	gtr	1 FPP + 1 IPP -> 1 GGPP + 1 PPI
429	Polyprenoids, Steroid, Phytoene biosynthesis	spnts2	spontaneous	IRR		1 GGPP + 1 IPP -> 1 PPPP + 1 PPI
430	Polyprenoids, Steroid, Phytoene	2.5.1.33	trans-pentaprenyltranstransferase	IRR		1 PPPP + 1 IPP -> 1 HPPP + 1 PPI

	biosynthesis					
431	Polyprenoids, Steroid, Phytoene biosynthesis	2.5.1.30	trans-hexaprenyltranstransferase	IRR		1 HPPP + 1 IPP -> 1 HEPPP + 1 PPI
432	Polyprenoids, Steroid, Phytoene biosynthesis	2.5.1.30	trans-hexaprenyltranstransferase	IRR		1 HEPPP + 1 IPP -> 1 OPP + 1 PPI
433	Polyprenoids, Steroid, Phytoene biosynthesis	2.5.1.11	trans-octaprenyltranstransferase	IRR		1 OPP + 1 IPP -> 1 NPP + 1 PPI
434	NAG & NAM biosynthesis	2.6.1.16	Glucosamine--fructose-6-phosphate aminotransferase (isomerizing)	IRR	glmS2	1 F6P + 1 GLN -> 1 GA6P + 1 GLT
435	NAG & NAM biosynthesis	3.5.99.6	Glucosamine-6-phosphate isomerase	IRR	nagB	1 GA6P + 1 H2O -> 1 NH3 + 1 F6P
436	NAG & NAM biosynthesis	3.5.1.25	N-acetylglucosamine-6-phosphate deacetylase	IRR		1 NADGLUCOSA6P + 1 H2O -> 1 GA6P + 1 AC
437	NAG & NAM biosynthesis	5.4.2.10	phosphoglucosamine mutase	REV		1 GA6P -> 1 GA1P
438	NAG & NAM biosynthesis	2.3.1.157	Glucosamine-1-phosphate N-acetyltransferase	IRR		1 GA1P + 1 ACCOA -> 1 NAGA1P + 1 COA
439	NAG & NAM biosynthesis	2.7.7.23	UDP-N-acetylglucosamine pyrophosphorylase	IRR		1 NAGA1P + 1 UTP -> 1 UDPNAG + 1 PPI
440	NAG & NAM biosynthesis	2.5.1.7	UDP-N-acetylglucosamine 1-carboxyvinyltransferase	IRR	murA	1 UDPNAG + 1 PEP -> 1 UDPNACVG + 1 PI
441	NAG & NAM	1.1.1.158	UDP-N-acetylmuramate dehydrogenase	IRR	murB	1 NADPH + 1 UDPNACVG + 1 H -> 1 NADP + 1 UDPNAM

	biosynthesis					
442	NAG & NAM biosynthesis	6.3.2.8	UDP-N-acetylmuramate--alanine ligase	IRR	murC	1 ALA + 1 UDPNAM + 1 ATP -> 1 UDPACMURALA + 1 PI + 1 ADP
443	NAG & NAM biosynthesis	5.1.1.3	Glutamate racemase	REV		1 GLT -> 1 GLU
444	NAG & NAM biosynthesis	6.3.2.9	UDP-N-acetylmuramoylalanine--D-glutamate ligase	IRR	murD	1 UDPACMURALA + 1 GLT + 1 ATP -> 1 UDPAAGLU + 1 PI + 1 ADP
445	NAG & NAM biosynthesis	6.3.2.9	UDP-N-acetylmuramoylalanyl-D-glutamate--2,6-diaminopimelate ligase	IRR	murE	1 UDPAAGLU + 1 MDAPIM + 1 ATP -> 1 UDPAAGMDHDIOATE + 1 PI + 1 ADP + 1 H
446	bAlanine metabolism	2.1.2.11	3-methyl-2-oxobutanoate hydroxymethyltransferase	IRR	panB	1 KISOVALERATE + 1 METHTHF + 1 H2O -> 1 DDPAN + 1 THF
447	bAlanine metabolism	1.1.1.169	2-dehydropantoate 2-reductase	IRR		1 DDPAN + 1 NADPH + 1 H -> 1 PANTOATE + 1 NADP
448	bAlanine metabolism	6.3.2.1	Pantoate--beta-alanine ligase	IRR		1 BALA + 1 PANTOATE + 1 ATP -> 1 PANT + 1 PPI + 1 AMP
449	bAlanine metabolism	2.6.1.19	4-aminobutyrate aminotransferase	REV	gabT	1 BALA + 1 AKG -> 1 MSALD + 1 GLT
450	bAlanine metabolism	1.2.1.15	malonate-semialdehyde dehydrogenase	IRR		1 MSALD + 1 H2O + 1 NAD -> 1 MALONATE + 1 NADH + 1 H
451	Coenzyme-A biosynthesis	2.7.1.33	Pantothenate kinase	IRR	coaA	1 PANT + 1 ATP -> 1 PANTOTHENP + 1 ADP
452	Coenzyme-A biosynthesis	6.3.2.5	Phosphopantothenate--cysteine ligase	IRR		1 PANTOTHENP + 1 CYS + 1 CTP -> 1 R4PPTCYC + 1 PPI + 1 CMP
453	Coenzyme-A biosynthesis	4.1.1.36	Phosphopantothenoylcysteine decarboxylase	IRR		1 R4PPTCYC -> 1 PANTP + 1 CO2

454	Coenzyme-A biosynthesis	2.7.7.3	Pantetheine-phosphate adenylyltransferase	IRR	coaD	1 PANTP + 1 ATP -> 1 DPCOA + 1 PPI
455	Coenzyme-A biosynthesis	2.7.1.24	Dephospho-CoA kinase	IRR	coaE	1 DPCOA + 1 ATP -> 1 COA + 1 ADP
456	Coenzyme-A biosynthesis	2.7.8.7	Holo-[acyl-carrier protein] synthase	IRR	acpS	1 COA -> 1 PAP + 1 ACP
457	Coenzyme-A biosynthesis	3.1.3.7	3',5'-bisphosphate nucleotidase	IRR		1 PAP + 1 H2O -> 1 PI + 1 AMP
458	Coenzyme-A biosynthesis	3.1.4.14	[acyl-carrier-protein] phosphodiesterase	IRR		1 ACP -> 1 PANTP
459	Nicotinamidenucleotides NAD+, NADP+ biosynthesis	3.5.1.19	nicotinamidase	IRR		1 NICOTINAMIDE + 1 H2O -> 1 NAC + 1 NH3
460	Nicotinamidenucleotides NAD+, NADP+ biosynthesis	2.4.2.1	Purine-nucleoside phosphorylase	IRR		1 R1P + 1 NAC -> 1 PI + 1 NACD
461	Nicotinamidenucleotides NAD+, NADP+ biosynthesis	2.4.2.11	Nicotinate phosphoribosyltransferase	IRR		1 NAC + 1 PRPP -> 1 NACN + 1 PPI
462	Nicotinamidenucleotides NAD+, NADP+ biosynthesis	3.1.3.5	Nicotinate D-ribonucleotide phosphohydrolase	IRR		1 NACD + 1 PI -> 1 NACN + 1 H2O

463	Nicotinamidenucleotides NAD+, NADP+ biosynthesis	3.6.1.22	Deamino-NAD+ nucleotidohydrolase	IRR		1 H2O + 1 NAAD -> 1 AMP + 1 NACN
464	Nicotinamidenucleotides NAD+, NADP+ biosynthesis	2.7.7.18	Nicotinate-nucleotide adenylyltransferase	IRR		1 ATP + 1 NACN -> 1 PPI + 1 NAAD
465	Nicotinamidenucleotides NAD+, NADP+ biosynthesis	2.4.2.19	Nicotinate-nucleotide pyrophosphorylase (carboxylating)	IRR	nadC	1 PRPP + 1 QUINOLINATE + 1 H -> 1 CO2 + 1 PPI + 1 NACN
466	Nicotinamidenucleotides NAD+, NADP+ biosynthesis	SCO2162	quinolinate synthetase	IRR		1 ISUCC + 1 DHAP -> 1 QUINOLINATE + 2 H2O + 1 PI
467	Nicotinamidenucleotides NAD+, NADP+ biosynthesis	1.4.3.16	L-aspartate oxidase	IRR	nadB	1 FAD + 1 ASP -> 1 FADH2 + 1 ISUCC
468	Nicotinamidenucleotides NAD+, NADP+ biosynthesis	1.4.3.16	L-aspartate oxidase	IRR	nadB	1 O2 + 1 ASP -> 1 H2O2 + 1 ISUCC
469	Nicotinamidenucleotides NAD+,	1.4.3.16	L-aspartate oxidase	IRR	nadB	1 UBIQON + 1 ASP -> 1 UBIQOL + 1 ISUCC

	NADP+ biosynthesis					
470	Nicotinamidenucleotides NAD+, NADP+ biosynthesis	1.4.3.16	L-aspartate oxidase	IRR	nadB	1 MK + 1 ASP -> 1 MKH2 + 1 ISUCC
471	Nicotinamidenucleotides NAD+, NADP+ biosynthesis	6.3.5.1	NAD(+) synthetase (glutamine-hydrolysing)	IRR	nadE1	1 ATP + 1 NAAD + 1 GLN + 1 H2O -> 1 AMP + 1 PPI + 1 NAD + 1 GLT
472	Nicotinamidenucleotides NAD+, NADP+ biosynthesis	3.6.1.22	NAD+ diphosphatase	IRR		1 H2O + 1 NAD -> 1 AMP + 1 NMN
473	Nicotinamidenucleotides NAD+, NADP+ biosynthesis	2.7.7.18	Nicotinate-nucleotide adenylyltransferase	IRR		1 ATP + 1 NMN + 1 H -> 1 PPI + 1 NAD
474	Nicotinamidenucleotides NAD+, NADP+ biosynthesis	3.1.3.5	Adenosine kinase	IRR		1 RNICOT + 1 PI -> 1 NMN + 1 H2O
475	Nicotinamidenucleotides NAD+, NADP+ biosynthesis	2.4.2.1	Purine-nucleoside phosphorylase	REV		1 R1P + 1 NICOTINAMIDE -> 1 PI + 1 RNICOT

476	Nicotinamidenucleotides NAD+, NADP+ biosynthesis	1.6.1.2	NAD(P) transhydrogenase	REV	pntA AND pntB	1 NADH + 1 NADP -> 1 NAD + 1 NADPH
477	Nicotinamidenucleotides NAD+, NADP+ biosynthesis	2.7.1.23	NAD kinase	IRR		1 NAD + 1 ATP -> 1 NADP + 1 ADP
478	Nicotinamidenucleotides NAD+, NADP+ biosynthesis	1.6.5.3	NADH dehydrogenase (ubiquinone)	IRR		1 UBIQON + 1 NADH + 1 H -> 1 UBIQOL + 1 NAD
479	Nicotinamidenucleotides NAD+, NADP+ biosynthesis	1.8.1.9	thioredoxin reductase (NADPH)	IRR		1 OTHIO + NADPH + 1 H -> 1 RTHIO + NADP
480	Biotin biosynthesis	2.3.1.47	8-amino-7-oxononanoate synthase	REV		1 ALA + 1 CHEXANOYLCOA -> 1 CO2 + 1 COA + 1 A8OXO7NONANOATE
481	Biotin biosynthesis	2.6.1.62	Adenosylmethionine--8-amino-7-oxononanoate aminotransferase	REV	bioA	1 SAMET + 1 A8OXO7NONANOATE -> 1 SA4M2OXBUTANOATE + 1 DIAMINONONANOATE
482	Biotin biosynthesis	6.3.3.3	dethiobiotin synthetase	REV	bioD	1 CO2 + 1 DIAMINONONANOATE + 1 ATP -> 1 DETHIOBIOTIN + 1 PI + 1 ADP
483	Biotin biosynthesis	2.8.1.6	biotin synthase	REV	bioB	1 S + 1 DETHIOBIOTIN + 2 SAMET -> 1 BIOTIN + 2 CH33ADO + 2 MET
484	Biotin biosynthesis	6.3.4.15	biotin-[acetyl-CoA carboxylase] synthetase	IRR		1 ATP + 1 BIOTIN -> 1 PPI + 1 B5AMP

485	Biotin biosynthesis	6.3.4.15	biotin-[acetyl-CoA carboxylase] synthetase	IRR		1 B5AMP + 1 ACARB -> 1 AMP + 1 BCCP
486	Biotin biosynthesis	6.3.4.14	biotin-carboxyl-carrier-protein:carbon-dioxide ligase (ADP-forming)	IRR		1 ATP + 1 BCCP + 1 HCO3 -> 1 ADP + 1 CCCP + 1 PI
487	Folate biosynthesis	3.5.4.16	GTP cyclohydrolase I	IRR	folE	1 GTP + 1 H2O -> 1 FPNTP
488	Folate biosynthesis	3.5.4.16	GTP cyclohydrolase I	IRR	folE	1 H2O + 1 FPNTP -> 1 DPNTP + 1 FOR
489	Folate biosynthesis	3.5.4.16	GTP cyclohydrolase I	IRR	folE	1 DPNTP -> 1 DTTOAO
490	Folate biosynthesis	3.5.4.16	GTP cyclohydrolase I	IRR	folE	1 DTTOAO -> 1 AHTD + 1 H2O
491	Folate biosynthesis	3.1.3.1	alkaline phosphatase	IRR		1 AHTD + 3 H2O -> 1 DHP + 3 PI
492	Folate biosynthesis	4.1.2.25	Dihydroneopterin aldolase	IRR	folB	1 DHP -> 1 GLAL + 1 AHHMP
493	Folate biosynthesis	2.7.6.3	2-amino-4-hydroxy-6-hydroxymethyldihydropteridine pyrophosphokinase	IRR	folK	1 AHHMP + 1 ATP -> 1 AHHMD + 1 AMP
494	Folate biosynthesis	6.3.5.8	AminodeoxyCHOR synthase	IRR		1 GLN + 1 CHOR -> 1 ADCHOR + 1 GLT
495	Folate biosynthesis	4.1.3.38	AminodeoxyCHOR lyase	IRR	add	1 ADCHOR -> 1 PABA + 1 PYR
496	Folate biosynthesis	2.5.1.15	Dihydropteroate synthase	IRR	folP	1 PABA + 1 AHHMD -> 1 DHPT + 1 PPI
497	Folate	2.5.1.15	Dihydropteroate synthase	IRR	folP	1 PABA + 1 AHHMP -> 1 DHPT + 1 H2O

	biosynthesis					
498	Folate biosynthesis	6.3.2.12or6.3.2.17	dihydrofolate synthetase	IRR	fpgS	1 GLT + 1 DHPT + 1 ATP -> 1 DHF + 1 PI + 1 ADP
499	Folate biosynthesis	1.5.1.3	Dihydrofolate reductase	REV	add	1 NADPH + 1 DHF + 1 H -> 1 NADP + 1 THF
500	Folate biosynthesis	6.3.2.17	tetrahydrofolate synthase	IRR	fpgS	1 THF + 1 GLT + 1 ATP -> 1 THFGLU + 1 ADP + 1 PI + 1 H
501	One carbon pool	3.5.4.9	Methenyltetrahydrofolate cyclohydrolase	REV	folD	1 METHENYLTHF + 1 H2O -> 1 F10THF + 1 H
502	One carbon pool	1.5.1.5	Methylenetetrahydrofolate dehydrogenase (NADP+)	REV	folD	1 METHTHF + 1 NADP -> 1 NADPH + 1 METHENYLTHF
503	One carbon pool	2.1.2.10	Aminomethyltransferase	IRR	gcvT	1 F10THF + 1 H2O -> 1 F5THF + 1 H
504	One carbon pool	2.1.2.10	Aminomethyltransferase	IRR	gcvT	1 METHENYLTHF + 1 H2O -> 1 F5THF + 1 H
505	One carbon pool	1.5.1.20	Methylenetetrahydrofolate reductase (NADPH)	IRR	metF	1 METHTHF + 1 NADPH + 1 H -> 1 MTHF + 1 NADP
506	One carbon pool	3.5.1.10	Formyltetrahydrofolate deformylase	IRR		1 F10THF + 1 H2O -> 1 THF + 1 FOR
507	Riboflavinvitamin B2, FMN and FAD biosynthesis	3.5.4.25	3,4-dihydroxy-2-butanone 4-phosphate synthase	IRR	ribAB	1 RL5P -> 1 HBUTP + 1 FOR
508	Riboflavinvitamin B2, FMN and FAD biosynthesis	3.5.4.25	GTP cyclohydrolase II	IRR	ribAB	1 GTP + 3 H2O -> 1 PPI + 1 D6RP5P + 1 FOR
509	Riboflavinvitamin B2, FMN and FAD biosynthesis	3.5.4.26	diaminohydroxyphosphoribosylaminopyrimidine deaminase	IRR	ribD	1 D6RP5P + 1 H2O -> 1 A6RP5P + 1 NH3

510	Riboflavinvitamin B2, FMN and FAD biosynthesis	1.1.1.193	5-amino-6-(5-phosphoribosylamino)uracil reductase	IRR		1 A6RP5P + 1 NADPH + 1 H-> 1 A6RP5P2 + 1 NADP
511	Riboflavinvitamin B2, FMN and FAD biosynthesis	3.1.3.-	pyrimidine phosphatase	IRR		1 A6RP5P2 + 1 H2O -> 1 A6RP + 1 PI
512	Riboflavinvitamin B2, FMN and FAD biosynthesis	2.5.1.9	Riboflavin synthase	IRR		1 A6RP + 1 HBUTP -> 1 D8RL + 2 H2O + 1 PI
513	Riboflavinvitamin B2, FMN and FAD biosynthesis	2.5.1.9	Riboflavin synthase	IRR		2 D8RL -> 1 A6RP + 1 RIBOFLAVIN
514	Riboflavinvitamin B2, FMN and FAD biosynthesis	2.7.1.26	Riboflavin kinase	IRR		1 RIBOFLAVIN + 1 ATP -> 1 FMN + 1 ADP
515	Riboflavinvitamin B2, FMN and FAD biosynthesis	2.7.7.2	FMN adenylyltransferase	IRR		1 FMN + 1 ATP -> 1 FAD + 1 PPI
516	Thiamine metabolism	2.7.1.89	Thiamine kinase	REV	add	1 THIAMINE + 1 ATP -> 1 THP + 1 ADP
517	Thiamine metabolism	2.7.1.49	Hydroxymethylpyrimidine kinase	REV		1 ATP + 1 HMP -> 1 ADP + 1 AHMMPYRP
518	Thiamine metabolism	2.7.4.7	Phosphomethylpyrimidine kinase	REV	SC7A1.07	1 AHMMPYRP + 1 ATP -> 1 AHMMPYRPP + 1 ADP
519	Thiamine metabolism	2.5.1.3	Thiamine-phosphate pyrophosphorylase	REV		1 THZP + 1 AHMMPYRPP + 1 H -> 1 THP + 1 PPI

520	Thiamine metabolism	ADD	ADD	IRR		1 GAP + 1 PYR -> 1 HZP
521	Thiamine metabolism	2.7.1.50	ATP:4-methyl-5-(2-hydroxyethyl)-thiazole 2-phosphotransferase	IRR		1 HZP + 1 ATP -> 1 THZP + 1 ADP
522	Thiamine metabolism	2.7.4.16	Thiamine-phosphate kinase	REV		1 THP + 1 ATP -> 1 THPP + 1 ADP
523	Thiamine metabolism	3.6.1.15	nucleoside triphosphate phosphohydrolase	IRR		1 THPP + 1 H2O -> 1 THP + 1 PI
524	L-Arabinose metabolism	1.1.1.21	aldehyde reductase	REV		1 LARABINOSE + 1 NADH + 1 H -> 1 ARABITOL + 1 NAD
525	L-Arabitol metabolism	1.1.1.13	L-arabinitol 2-dehydrogenase	REV		1 ARABITOL + 1 NAD -> 1 LRIBULOSE + 1 NADH + 1 H
526	L-Arabitol metabolism	1.1.1.12	L-arabinitol 4-dehydrogenase	REV		1 ARABITOL + 1 NAD -> 1 LXYLULOSE + 1 NADH + 1 H
527	L-Arabinose synthesis	5.3.1.4	L-arabinose isomerase	REV		1 LRIBULOSE -> 1 LARABINOSE
528	L-Ribulose synthesis	2.7.1.16	ribulokinase	IRR		1 LRIBULOSE + 1 ATP -> 1 LRIBULOSE5P + 1 ADP
529	L-Ribulose synthesis	5.-.-.-	isomerase	REV		1 LRIBULOSE5P -> 1 LXYLULOSE5P
530	L-Ribulose synthesis	5.1.3.4	L-ribulose-5-phosphate 4-epimerase	REV		1 LRIBULOSE5P -> 1 X5P
531	L-Lyxose metabolism	2.7.1.53	L-xylulokinase	REV		1 LXYLULOSE5P + 1 ADP -> 1 LXYLULOSE + 1 ATP
532	L-Lyxose metabolism	5.3.1.-	sugar isomerase	REV		1 LLYXOSE -> 1 LXYLULOSE

533	L-Lyxose metabolism	5.3.1.15	D-lyxose ketol-isomerase	REV		1 LLYXOSE -> 1 DLYXOSE
534	L-Xylitol metabolism	1.1.1.15	D-iditol 2-dehydrogenase	IRR		1 XYLITOL + 1 NAD -> 1 LXYLULOSE + 1 NADH + 1 H
535	L-Xylitol metabolism	1.1.1.9	D-xylulose reductase	IRR		1 XYLITOL + 1 NAD -> 1 DXYL + 1 NADH + 1 H
536	L-Xylose metabolism	1.1.1.21	aldehyde reductase	REV		1 XYL + 1 NADH + 1 H -> 1 XYLITOL + 1 NAD
537	L-Xylose metabolism	5.3.1.5	Xylose isomerase	REV	xylA	1 XYL -> 1 DXYL
538	Arabitol metabolism	1.1.1.11	D-arabinitol 4-dehydrogenase	IRR		1 DARABITOL + 1 NAD -> 1 DXYL + 1 NADH + 1 H
539	Xylose metabolism	5.3.1.15	D-lyxose ketol-isomerase	REV		1 DXYL -> 1 DLYXOSE
540	Xylose metabolism	2.7.1.17	Xylulokinase	IRR	xylB	1 DXYL + 1 ATP -> 1 X5P + 1 ADP
541	Mannose Metabolism	2.7.1.7	Mannokinase	IRR		1 MAN + 1 ATP -> 1 MAN6P + 1 ADP
542	Mannose Metabolism	5.3.1.8	Mannose-6-phosphate isomerase	REV	manA	1 MAN6P -> 1 F6P
543	Mannose Metabolism	5.4.2.8	Phosphomannomutase	REV		1 MAN1P -> 1 MAN6P
544	Mannose Metabolism	2.7.7.13	Mannose-1-phosphate guanylyltransferase	IRR		1 MAN1P + 1 GTP -> 1 GMAN + 1 PPI
545	Mannose Metabolism	4.2.1.47	GDP-mannose 4,6-dehydratase	IRR	galE1	1 GMAN -> 1 G4D6DMAN + 1 H2O

546	Fructose metabolism	2.7.1.13	D-fructose 1-phosphotransferase	IRR		1 ATP + 1 FRU -> 1 ADP + 1 F1P
547	Fructose metabolism	2.7.1.56	1-phosphofructokinase	IRR		1 ATP + 1 F1P -> 1 ADP + 1 FDP
548	Fructose metabolism	4.1.2.13	Fructose-bisphosphate aldolase	REV	fba	1 F1P -> 1 T3 + 1 GAP
549	Fructose metabolism	2.7.1.4	Fructokinase	IRR		1 FRU + 1 ATP -> 1 F6P + 1 ADP
550	Fructose metabolism	5.3.1.5	Xylose isomerase	REV		1 GLC -> 1 FRU
551	Galactose metabolism	2.7.1.6	Galactokinase	REV	galK	1 GALC + 1 ATP -> 1 GALC1P + 1 ADP
552	Galactose metabolism	2.7.7.10	UTP--hexose-1-phosphate uridylyltransferase	IRR	galT	1 GALC1P + 1 UTP -> 1 UDPGAL + 1 PPI
553	Galactose metabolism	5.1.3.2	UDP-glucose 4-epimerase	REV	galE2	1 UDPGAL -> 1 UDPGLU
554	Galactose metabolism	1.1.1.22	UDP-glucose 6-dehydrogenase	IRR		1 UDPGLU + 2 NAD + 1 H2O -> 1 UDPGLUCUR + 2 NADH + 2 H
555	Galactose metabolism	2.7.7.9	UTP--glucose-1-phosphate uridylyltransferase	REV	gtaB	1 G1P + 1 UTP -> 1 UDPGLU + 1 PPI
556	Galactose metabolism	2.4.1.15	trehalose-phosphate synthase	IRR		1 UDPGLU + 1 G6P -> 1 UDP + TRE6P
557	Galactose metabolism	5.4.99.16	trehalose synthase treS (TreS Pathway)	REV		1 MLT -> 1 TRE
558	Galactose metabolism	3.2.1.22	Alpha-galactosidase	IRR		1 GALACTINOL + 1 H2O -> 1 GLAC + 1 MYOINOSITOL

559	Galactose metabolism	3.2.1.22	Alpha-galactosidase	IRR		1 RAF + 1 H2O -> 1 GLAC + 1 SUCROSE
560	Galactose metabolism	3.2.1.22	Alpha-galactosidase	IRR		1 STACHYOSE + 1 H2O -> 1 RAF + 1 GLAC
561	Galactose metabolism	3.2.1.22	Alpha-galactosidase	IRR		1 EPM + 1 H2O -> 1 MAN + 1 GLAC
562	Galactose metabolism	3.2.1.22	Alpha-galactosidase	IRR		1 GGL + 1 H2O -> 1 GL + 1 GLAC
563	Galactose metabolism	3.2.1.22	Alpha-galactosidase	IRR		1 MELIT + 1 H2O -> 1 SOT + 1 GLAC
564	Mannitol metabolism	1.1.1.17	mannitol-1-phosphate 5-dehydrogenase	IRR		1 MANNITOL1P + 1 NAD -> 1 F6P + 1 NADH
565	L-Rhamnose metabolism	5.3.1.14	L-rhamnose isomerase	REV		1 RMN -> 1 RHAMN
566	L-Rhamnose metabolism	2.7.1.5	rhamnulokinase	IRR		1 RHAMN + 1 ATP -> 1 ADP + 1 RHAMN1P
567	L-Rhamnose metabolism	4.1.2.19	rhamnulose-1-phosphate aldolase	REV		1 RHAMN1P -> 1 DHAP + 1 LACTAL
568	L-Rhamnose metabolism	1.2.1.22	lactaldehyde dehydrogenase	REV		1 LACTAL + 1 NAD + 1 H2O -> 1 LLAC + 1 NADH + 1 H
569	Salicin metabolism	2.7.1.63	Polyphosphate-glucose phosphotransferase	IRR	ppgK	1 bGLC + 1 PIn -> 1 bG6P + 1 PIn-1
570	Salicin metabolism	2.7.1.63	Polyphosphate-glucose phosphotransferase	IRR	ppgK	1 GLC + 1 PIn -> 1 G6P + 1 PIn-1
571	Salicin metabolism	5.1.3.3	Aldose 1-epimerase	REV		1 bGLC -> 1 GLC

572	Salicin metabolism	3.2.1.86	6-phospho-beta-glucosidase	IRR		1 SAL6P + 1 H2O -> 1 G6P + 1 HXMP
573	Salicin metabolism	3.2.1.86	6-phospho-beta-glucosidase	IRR		1 SAL6P + 1 H2O -> 1 bG6P + 1 HXMP
574	Maltose metabolism	3.2.1.20	Alpha-glucosidase	IRR		1 MLT + 1 H2O -> 2 GLC
575	Sucrose metabolism	3.2.1.20	Alpha-glucosidase	IRR		1 SUCROSE + 1 H2O -> 1 GLC + 1 FRU
576	Lactose metabolism	3.2.1.23	Beta-galactosidase	IRR		1 LACTOSE + 1 H2O -> 1 GLAC + 1 GLC
577	Melibiose metabolism	3.2.1.22	Alpha-galactosidase	IRR		1 MELI + 1 H2O -> 1 GLC + 1 GLAC
578	Cellobiose metabolism	3.2.1.21	beta-glucosidase	IRR		1 CELB + 1 H2O -> 2 GLC
579	Trehalose biosynthesis	3.1.3.12	trehalose phosphatase	IRR		1 TRE6P + 1 H2O -> 1 TRE + 1 PI
580	Trehalose biosynthesis	3.2.1.28	alpha,alpha-trehalase	IRR		1 TRE + 1 H2O -> 2 GLC
581	Nucleotide sugar metabolism	2.7.7.33	glucose-1-phosphate cytidylyltransferase	IRR	glgCI	1 G1P + 1 DTTP -> 1 TDPDGLUCOSE + 1 PPI
582	Nucleotide sugar metabolism	5.1.3.2	UDP-glucose 4-epimerase	REV	(galE2 OR galE2 OR galE2 OR galE2)	1 TDPDGLUCOSE -> 1 TDPGAL
583	Starch metabolism	2.4.1.1	starch phosphorylase	IRR	glgP	1 STARCHn + 1 PI -> 1 STARCHn-1 + 1 G1P
584	Glyoxylate and dicarboxylate	4.1.1.47	Tartronate-semialdehyde synthase	IRR		2 GLX -> 1 CO2 + 1 TARTALD

	metabolism					
585	Glyoxylate and dicarboxylate metabolism	2.6.1.45	L-Serine:glyoxylate aminotransferase	IRR		1 GLX + SER -> 1 HYDROXYPYRUVATE + 1 GLY
586	Glyoxylate and dicarboxylate metabolism	5.3.1.22	hydroxypyruvate isomerase	REV		1 HYDROXYPYRUVATE -> 1 TARTALD
587	Glyoxylate and dicarboxylate metabolism	1.1.1.60	2-hydroxy-3-oxopropionate reductase	IRR		1 NADPH + 1 TARTALD + 1 H -> 1 NADP + 1 DGLYCERATE
588	Glyoxylate and dicarboxylate metabolism	1.11.1.6	Catalase	IRR	(kata OR cpeB OR catB OR SCO6204 OR katA2)	2 H2O2 -> 2 H2O + 1 O2
589	Glyoxylate and dicarboxylate metabolism	1.2.1.21	glycolaldehyde dehydrogenase	IRR		1 GLAL + 1 NAD + 1 H2O -> 1 NADH + 1 GLYCOLATE + 1 H
590	Glyoxylate and dicarboxylate metabolism	1.1.3.15	(S)-2-hydroxy-acid oxidase	IRR		1 UBIQON + 1 GLYCOLATE -> 1 UBIQOL + 1 GLX
591	Glyoxylate and dicarboxylate metabolism	1.1.3.15	(S)-2-hydroxy-acid oxidase	IRR		1 MK + 1 GLYCOLATE -> 1 MKH2 + 1 GLX
592	Glyoxylate and dicarboxylate	1.1.3.15	(S)-2-hydroxy-acid oxidase	IRR		1 O2 + 1 GLYCOLATE -> 1 H2O2 + 1 GLX

	metabolism					
593	Glycerate metabolism	2.7.1.31	Glycerate kinase	IRR		1 DGLYCERATE + 1 ATP -> 1 3PG + 1 ADP
594	Glycerol metabolism	2.7.1.30	Glycerol kinase	IRR	glpK2	1 GL + 1 ATP -> 1 GL3P + 1 ADP
595	Glycerol metabolism	1.1.99.5	Glycerol-3-phosphate dehydrogenase	IRR	glpD	1 GL3P + 1 UBIQON -> 1 DHAP + 1 UBIQOL
596	Glycerol metabolism	1.1.99.5	Glycerol-3-phosphate dehydrogenase	IRR	glpD	1 GL3P + 1 MK -> 1 DHAP + 1 MKH2
597	Glycerol metabolism	1.1.99.5	Glycerol-3-phosphate dehydrogenase	IRR	glpD	1 GL3P + 1 FAD -> 1 DHAP + 1 FADH2
598	Glycerol metabolism	1.1.1.2	Alcohol dehydrogenase (NADP+)	REV		1 NADP + 1 GL -> 1 NADPH + 1 T3 + 1 H
599	Glycerol metabolism	1.2.1.3	Aldehyde dehydrogenase (NAD+)	IRR	thcA	1 T3 + 1 NAD + 1 H2O -> 1 DGLYCERATE + 1 NADH + 1 H
600	Glycerol metabolism	1.1.1.1	alcohol dehydrogenase	REV		1 T3 + 1 NADH + 1 H -> 1 GL + 1 NAD
601	Glycerol metabolism	1.1.1.6	glycerol dehydrogenase	IRR		1 GL + 1 NAD -> 1 GLYCERON + 1 NADH + 1 H
602	Glycerol metabolism	2.7.1.29	glycerone kinase	IRR		1 GLYCERON + 1 ATP -> 1 ADP + 1 DHAP
603	Propanoate metabolism	added	phosphate acetyltransferase	IRR		1 OXOBUTANOATE + 1 PI + 1 O2 -> 1 PROPIONYLP + 1 CO2 + 1 H2O
604	Propanoate metabolism	2.7.2.1	acetate kinase	REV	proB	1 PROPIONYLP + 1 ADP -> 1 ATP + 1 PROPANOATE
605	Propanoate	6.2.1.1	acetyl-CoA synthetase	IRR		1 ATP + 1 PROPANOATE -> 1 PPI + 1 PADNLT

	metabolism					
606	Propanoate metabolism	6.2.1.1	acetyl-CoA synthetase	IRR		1 PADNLT + 1 COA -> 1 AMP + 1 PROPIONYLCOA
607	Propanoate metabolism	2.3.1.8	phosphate acetyltransferase	REV	pta	1 PROPIONYLCOA + 1 PI -> 1 PROPIONYLP + 1 COA
608	Propanoate metabolism	1.3.99.3	Butyryl-CoA dehydrogenase	IRR		1 PROPIONYLCOA + 1 FAD -> 1 ACRCOA + 1 FADH2
609	Propanoate metabolism	4.2.1.17	enoyl-CoA hydratase	IRR		1 ACRCOA + 1 H2O -> 1 HPCOA
610	Propanoate metabolism	add	unknown enzyme	IRR		1 HPCOA + 1 NADP -> 1 OPCOA + 1 NADPH + 1 H
611	Propanoate metabolism	add5	unknown enzyme	IRR		1 OPCOA + 1 O2 + 1 NADPH + 1 H -> 1 MALCOA + 1 NADP + 1 H2O
612	Benzoate metabolism	1.14.13.82	vanillate monooxygenase	IRR		1 VANILLATE + 0.5 O2 + 1 NADH + 1 H -> 1 DIBENZOATE + 1 NAD + 1 H2O + 1 FOR
613	Benzoate metabolism	1.13.11.3	Protocatechuate 3,4-dioxygenase	IRR	(pcaG,pcaH)	1 O2 + 1 DIBENZOATE -> 1 C3MUCO
614	Benzoate metabolism	5.5.1.2	3-carboxy-cis,cis-muconate cycloisomerase	IRR	pcaB	1 C3MUCO -> 1 C2DOF5ACET
615	Benzoate metabolism	4.1.1.44	4-carboxymuconolactone decarboxylase	IRR	pcaL	1 C2DOF5ACET -> 1 CO2 + 1 OAELAC
616	Benzoate metabolism	3.1.1.24	3-oxoadipate enol-lactonase	IRR	pcaL	1 OAELAC + 1 H2O -> 1 KADIPATE
617	Benzoate metabolism	2.8.3.6	3-oxoacid CoA-transferase	IRR		1 SUCCOA + 1 KADIPATE -> 1 SUCC + 1 KADCOA
618	Benzoate	2.3.1.16	acetyl-CoA acetyltransferase	IRR		1 KADCOA + 1 COA -> 1 ACCOA + 1 SUCCOA

	metabolism					
619	Benzoate metabolism	1.14.13.1	Salicylate 1-monooxygenase	IRR		1 O2 + 1 NADH + 1 SALICYLATE + 1 H -> 1 CO2 + 1 NAD + 1 CATECHOL + 1 H2O
620	Sulphate metabolism	2.7.7.4	Sulfate adenylyltransferase	IRR	moeB	1 SLF + 1 ATP + 1 H2O -> 1 APS + 1 PPI + 1 H
621	Sulphate metabolism	2.7.1.25	Adenylylsulfate kinase	IRR	cysC	1 APS + 1 ATP -> 1 PAPS + 1 ADP
622	Sulphate metabolism	1.8.4.8	Phosphoadenylyl-sulfate reductase (thioredoxin)	IRR	cysH	1 PAPS + 1 RTHIO -> 1 PAP + 1 H2SO3 + 1 OTHIO
623	Sulphate metabolism	1.8.1.2	Sulfite reductase (NADPH)	REV	add	3 NADPH + 1 H2SO3 -> 3 H2O + 3 NADP + 1 H2S
624	Nitrogen metabolism	1.7.99.4	nitrate reductase	IRR		1 NO3 + 1 MKH2 -> 1 MK + 1 NO2 + 2 H
625	Nitrogen metabolism	1.7.7.1	nitrite/sulphite reductase	IRR		1 NO2 + 6 FERO + 7 H -> 1 NH3 + 6 FERI + 2 H2O
626	Nitrogen metabolism	1.7.1.4	nitrite/sulphite reductase	IRR		1 NO2 + 3 NADPH + 3 H -> 1 NH3 + 3 NADP + 1 H2O
627	Nitrogen metabolism	4.2.1.1	Carbonate dehydratase	REV		1 CO2 + 1 H2O -> 1 H2CO3
628	Glutathione metabolism	1.11.1.9	glutathione peroxidase	IRR		2 GSH + 1 H2O2 -> 1 GSSG + 2 H2O
629	Glutathione metabolism	2.3.2.2	gamma-glutamyltransferase	IRR		1 GSH + 1 H2O -> 1 CYSGLY + 1 GLT
630	Glutathione metabolism	3.4.11.2	putative gamma-glutamyltranspeptidase	IRR		1 CYSGLY + 1 H2O -> 1 CYS + 1 GLY
631	Betaine	1.1.99.1	Choline dehydrogenase	IRR		1 CHOLINE + 1 FAD -> 1 BETAINEALD + 1 FADH2

	biosynthesis					
632	Betaine biosynthesis	1.1.3.17	betaine aldehyde:oxygen oxidoreductase	IRR		1 BETAINEALD + 1 H2O + 1 O2 -> 1 BETAINE + 1 H2O2
633	Betaine biosynthesis	1.2.1.8	Betaine-aldehyde dehydrogenase	IRR		1 BETAINEALD + 1 NAD + 1 H2O -> 1 BETAINE + 1 NADH + 2 H
634	Menaquinone biosynthesis	5.4.4.2	isoCHOR synthase	IRR	menF	1 CHOR -> 1 ICHOR
635	Menaquinone biosynthesis	2.2.1.9	2-succinyl-6-hydroxy-2,4-cyclohexadiene-1-carboxylate synthase	IRR	menD	1 ICHOR + 1 AKG -> 1 SHCHC + 1 PYR + 1 CO2
636	Menaquinone biosynthesis	4.2.1.-	o-succinylbenzoate synthase	IRR	menC	1 SHCHC -> 1 OSB + 1 H2O
637	Menaquinone biosynthesis	6.2.1.26	o-succinylbenzoate-CoA ligase	IRR		1 OSB + 1 ATP + 1 COA -> 1 AMP + 1 PPI + 1 OSBCOA
638	Menaquinone biosynthesis	4.1.3.36	naphthoate synthase	IRR		1 OSBCOA -> 1 DHN + 1 COA
639	Menaquinone biosynthesis	2.5.1.-	1,4-dihydroxy-2-naphthoate prenyltransferase	IRR		1 DHN + 1 NPP -> 1 PPI + 1 CO2 + 1 DMK
640	Menaquinone biosynthesis	2.1.1.37	demethylmenaquinone methyltransferase	IRR		1 DMK + 1 SAMET -> 1 MKH2 + 1 SAH
641	Superoxide metabolism	1.15.1.1	Superoxide dismutase	IRR	(sodF2 OR sodF OR sodN)	2 SUPEROXIDE + 2 H -> 1 H2O2 + 1 O2
642	Ethanol metabolism	1.11.1.21	catalase	IRR	(kata OR cpeB OR catB OR SCO6204 OR katA2)	1 ETH + 1 H2O2 -> 2 H2O + 1 ACAL

643	Methanol metabolism	1.11.1.6	catalase	IRR	katA	1 METH + 1 H2O2 -> 1 FORMAL + 2 H2O
644	Methanol metabolism	1.2.1.2	formate dehydrogenase	IRR		1 FOR + 1 NAD -> 1 CO2 + 1 NADH + 1 H
645	Toluene metabolism	1.14.12.11	Toluene dioxygenase	IRR		1 TOLUENE + 1 O2 + 1 NADH + 1 H -> 1 NAD + 1 TOLUENECISDHDIOL
646	Toluene metabolism	4.1.3.-	cyclase	IRR		1 HKVALERATE -> 1 ACAL + 1 PYR
647	Phenylalanine metabolism	4.2.1.80	2-oxopent-4-enoate hydratase	IRR		1 OXOPENTENOATE + 1 H2O-> 1 HKVALERATE
648	Phenylalanine metabolism	4.2.1.-	2-oxopent-4-enoate hydratase	IRR		1 OXOPENTENOATE + 1 H2O -> 1 PYR + 1 ACAL
649	Porphyrin and chlorophyll Metabolsim	2.3.1.37	5-aminolevulinate synthase	IRR	add	1 GLY + 1 SUCCOA -> 1 A5LEVULINATE + 1 COA + 1 CO2
650	Porphyrin and chlorophyll Metabolsim	6.1.1.17	glutamate---tRNA ligase	IRR	gltX	1 ATP + 1 GLT -> 1 trnaGLT + 1 AMP + 1 PPI
651	Porphyrin and chlorophyll Metabolsim	1.2.1.70	Glutamate-1-semialdehyde 2,1-aminomutase	IRR	hemA	1 trnaGLT + 1 NADPH -> 1 GLUGSAL + 1 NADP
652	Porphyrin and chlorophyll Metabolsim	5.4.3.8	Glutamate-1-semialdehyde 2,1-aminomutase	IRR	(hemL OR hemL)	1 GLUGSAL -> 1 A5LEVULINATE
653	Porphyrin and chlorophyll	4.2.1.24	Porphobilinogen synthase	IRR	hemB	2 A5LEVULINATE -> 1 PORPHOBILINOGEN + 2 H2O

	Metabolsim					
654	Porphyrin and chlorophyll Metabolsim	2.5.1.61	Hydroxymethylbilane synthase	IRR	hemC	4 PORPHOBILINOGEN + 1 H2O -> 4 NH3 + 1 HYDROXYMETHYLBILANE
655	Porphyrin and chlorophyll Metabolsim	4.2.1.75	uroporphyrinogen-III synthase	IRR		1 HYDROXYMETHYLBILANE -> 1 UROPORPHYRINOGEN3 + 1 H2O
656	Porphyrin and chlorophyll Metabolsim	4.1.1.37	Uroporphyrinogen decarboxylase	IRR	hemE	1 UROPORPHYRINOGEN3 -> 4 CO2 + 1 COPROPORPHYRINOGEN
657	Porphyrin and chlorophyll Metabolsim	1.3.99.22	putative oxygen-independent coproporphyrinogen III oxidase	IRR		1 COPROPORPHYRINOGEN + 2 SAMET -> 1 PROTOPORPHYRINOGEN + 2 CO2 + 2 MET + 2 CH33ADO
658	Porphyrin and chlorophyll Metabolsim	1.3.3.4	Protoporphyrinogen oxidase	IRR		3 O2 + 2 PROTOPORPHYRINOGEN -> 2 PROTOPORPHYRIN + 6 H2O
659	Porphyrin and chlorophyll Metabolsim	4.99.1.1	Ferrochelatase	IRR	hemH	1 FE + 1 PROTOPORPHYRIN -> 2 H + 1 PROTOHEME
660	Porphyrin and chlorophyll Metabolsim	2.5.1.-	cytochrome oxidase assembly factor	IRR		1 PROTOHEME + 1 FPP + 1 H2O -> 1 HEMEO + 1 PPI
661	Porphyrin and chlorophyll Metabolsim	2.5.1.-	cytochrome oxidase assembly factor	IRR		1 HEMEO -> 1 HEME
662	Porphyrin and	2.1.1.107	uroporphyrin-III methyltransferase	IRR		1 UROPORPHYRINOGEN3 + 1 SAMET -> 1 SAH + 1 PRECORRIN1

	chlorophyll Metabolsim					
663	Porphyrin and chlorophyll Metabolsim	2.1.1.107	uroporphyrin-III methyltransferase	IRR		1 PRECORRIN1 + 1 SAMET -> 1 DSHCLORIN + 1 SAH
664	Porphyrin and chlorophyll Metabolsim	2.1.1.130	Precorrin-2 C20-methyltransferase	IRR	cobI	1 DSHCLORIN + 1 SAMET -> 1 SAH + 1 PRECORRIN3A
665	Porphyrin and chlorophyll Metabolsim	1.14.13.83	precorrin-3B synthase	IRR	add	1 PRECORRIN3A + 1 O2 + 1 NADH + 1 H -> 1 PRECORRIN3B + 1 NAD + 1 H2O
666	Porphyrin and chlorophyll Metabolsim	2.1.1.131	Precorrin-3B C17-methyltransferase	IRR		1 PRECORRIN3B + 1 SAMET -> 1 PRECORRIN4B + 1 SAH
667	Porphyrin and chlorophyll Metabolsim	2.1.1.133	Precorrin-4 C11-methyltransferase	IRR		1 PRECORRIN4B + 1 SAMET -> 1 PRECORRIN5 + 1 SAH
668	Porphyrin and chlorophyll Metabolsim	2.1.1.152	S-adenosyl-L-methionine:precorrin-5 C1-methyltransferase	IRR	cobF	1 PRECORRIN5 + 1 SAMET + 1 H2O -> 1 PRCN + 1 SAH + 1 AC
669	Porphyrin and chlorophyll Metabolsim	1.3.1.54	Precorrin-6X reductase	IRR		1 PRCN + 1 NADPH + 1 H -> 1 PRECORRIN6B + 1 NADP
670	Porphyrin and chlorophyll Metabolsim	2.1.1.132	Precorrin-6Y C5,15-methyltransferase (decarboxylating)	IRR		1 PRECORRIN6B + 2 SAMET -> 1 PRECORRIN8X + 2 SAH + 1 CO2

671	Porphyrin and chlorophyll Metabolsim	5.4.1.2	Precorrin-8X methylmutase	IRR		1 PRECORRIN8X -> 1 HGBYRINATE
672	Porphyrin and chlorophyll Metabolsim	6.3.5.9	Hydrogenobyrinic acid a,c-diamide synthase (glutamine-hydrolyzing)	IRR	cobB	1 HGBYRINATE + 2 GLN + 2 ATP + 2 H2O -> 1 HGBYRINATED + 2 GLT + 2 ADP + 2 PI
673	Porphyrin and chlorophyll Metabolsim	6.6.1.2	Cobaltochelatase	IRR	cobN	1 HGBYRINATED + 1 COBALT + 1 ATP + 1 H2O -> 1 CAACDIM2 + 1 ADP + 1 PI + 1 H
674	Porphyrin and chlorophyll Metabolsim	1.16.8.1	cob(II)yrinic acid a,c-diamide reductase	IRR		1 CAACDIM2 + 1 NADH -> 1 CAACDIM1 + 1 NAD
675	Porphyrin and chlorophyll Metabolsim	2.5.1.17	cob(I)yrinic acid a,c-diamide adenosyltransferase	REV		1 CAACDIM1 + 1 ATP -> 1 ACACDIM + 1 PI + 1 PPI
676	Porphyrin and chlorophyll Metabolsim	6.3.5.10	Adenosylcobyric acid synthase (glutamine-hydrolyzing)	IRR	cobQ	1 ACACDIM + 4 GLN + 4 ATP + 4 H2O -> 1 ACOBYRATE + 4 GLT + 4 ADP + 4 PI
677	Porphyrin and chlorophyll Metabolsim	6.3.1.10	adenosylcobinamide-phosphate synthase CobD	IRR		1 ACOBYRATE + 1 APROPANOL -> 1 ACOBINAMIDE
678	Porphyrin and chlorophyll Metabolsim	2.7.1.156	adenosylcobinamide kinase	IRR		1 ACOBINAMIDE + 1 ATP -> 1 ACOBINAMIDEP + 1 ADP
679	Porphyrin and chlorophyll	2.7.7.62	Adenosylcobinamide-phosphate guanylyltransferase	IRR		1 ACOBINAMIDEP + 1 GTP -> 1 ACOBINAMIDEGDP + 1 PPI

	Metabolsim					
680	Porphyrin and chlorophyll Metabolsim	2.4.2.21	Nicotinate-nucleotide-dimethylbenzimidazole phosphoribosyltransferase	IRR		1 NACN + 1 DBENZIMIDAZOLE -> 1 NAC + 1 ARIBAZOLE5P + 1 H
681	Porphyrin and chlorophyll Metabolsim	3.1.3.73	Nicotinate-nucleotide-dimethylbenzimidazole phosphoribosyltransferase	IRR	add	1 ARIBAZOLE5P + 1 H2O -> 1 ARIBAZOLE + 1 PI
682	Porphyrin and chlorophyll Metabolsim	2.7.8.26	adenosylcobinamide-GDP ribazoletransferase	IRR		1 ACOBINAMIDEGDP + 1 ARIBAZOLE -> 1 ACOBALAMIN + 1 GMP
683	Energy metabolism	3.6.3.14	H(+)-transporting two-sector ATPase	REV		1 H + 1 ATP + 1 H2O -> 4 Hxt + 1 PI + 1 ADP
684	Energy metabolism	3.6.1.1	putative inorganic pyrophosphatase	IRR	(ppa or hppa)	1 PPI + 1 H2O -> 2 PI
685	Energy metabolism	2.7.4.1	Polyphosphate kinase	REV	ppk	1 PIn-1 + 1 ATP -> 1 PIn + 1 ADP
686	Energy metabolism	1.18.1.2	putative ferredoxin/ferredoxin-NADP reductase	REV		1 FERO + 1 NADP -> 1 FERI + 1 NADPH + 1 H
687	Energy metabolism	1.10.3.-	cytochrome bd oxidase	IRR		1 MKH2 + 0.5 O2 + 4 H -> 1 MK + 4 Hxt + 1 H2O
688	Energy metabolism	1.10.3.-	cytochrome bc1-aa3 supercomplex	IRR		1 MKH2 + 0.5 O2 + 2 H -> 1 MK + 2 Hxt + 1 H2O
689		1.5.1.29	flavin reductase	REV		1 FAD + 1 NADH -> 1 FADH2 + 1 NAD
690	DHCHC biosynthesis		FkbO (chorismatase)	IRR	fkbO	1 CHOR + 1 H2O -> 1 DCDC + 1 PYR

691	DHCHC biosynthesis		FkbO (chorismatase)		fkbO	1 DCDC + 1 NADPH -> DHCHC + 1 NADP
692	Pipecolate biosynthesis		FkbL (Predicted lysine cyclodeaminase)	IRR	fkbL	1 LYS -> 1 PIPECOLATE + 1 NH3
693	Methoxymalonyl-ACP biosynthesis		FkbH AND FkbK AND FkbI AND FkbG	IRR	fkbH AND fkbK AND fkbI AND fkbG	1 DPG + 1 ACPJ + 2 NADP + 1 SAMET + 1 H2O -> 1 METHOXYMACPJ + 2 NADPH + 2 PI + 1 SAH
694	Propylmalonyl-CoA biosynthesis		TcsA AND TcsB AND TcsC	IRR	tcsA AND tcsB AND tcsC	1 PROPIONYLCOA + 1 MALCOA + 2 NADPH + 1 ATP + 2 H -> 1 PROPYLMCOA + 1 COA + 2 NADP + 1 ADP + 1 PI + 1 H2O
695	Allylmalonyl-CoA biosynthesis		TcsD	IRR	tcsD	1 PROPYLMCOA + 1 FAD -> 1 ALLYLMCOA + 1 FADH2 + 1 H
696	Ethylmalonyl-CoA biosynthesis		TcsC	IRR	tcsC	1 CROTCOA + 1 NADPH + 1 ATP + 1 HCO3 + 2 H -> 1 ETHYLMCOA + 1 NADP + 1 ADP + 1 PI + 2 H2O
697	FK506 biosynthesis		FkbB	IRR	fkbB	1 DHCHC + 1 ACP +1 ATP +1 NADPH + 1 H -> 1 DHCHCACP + 1 AMP +1 PPI + 1 NADP
698	FK506 biosynthesis		FkbB AND FkbC AND FkbA AND FkbP	IRR	fkbB AND fkbC AND fkbA AND fkbP	1 DHCHCACP + 5 SMMCOA + 2 MALCOA + 1 ALLYLMCOA + 2 METHOXYMACPJ + 1 PIPECOLATE + 12 NADPH -> 1 PREFK506 + 12 NADP + 1 ACP + 2 ACPJ + 10 CO2 + 8 H2O
699	FK506 biosynthesis		FkbD (Cytochrome P450) AND FkbM (SAM-dependent methyltransferase)	IRR	fkbD AND fkbM	1 PREFK506 + 1 SAMET + 2 NADPH + 2 O2 + 2 H -> 1 FK506 + 1 SAH + 2 NADP + 2 H2O
700	FK506D biosynthesis		FkbB AND FkbC AND FkbA AND FkbP	IRR	fkbB AND fkbC AND fkbA AND fkbP	1 DHCHCACP + 5 SMMCOA + 2 MALCOA + 1 PROPYLMCOA + 2 METHOXYMACPJ + 1 PIPECOLATE + 12 NADPH -> 1 PREFK506D + 12 NADP + 1 ACP + 2 ACPJ + 10 CO2 + 8 H2O
701	FK506D biosynthesis		FkbD (Cytochrome P450) AND FkbM (SAM-dependent methyltransferase)	IRR	fkbD AND fkbM	1 PREFK506D + 1 SAMET + 2 NADPH + 2 O2 + 2 H -> 1 FK506D + 1 SAH + 2 NADP + 2 H2O

702	FK520 biosynthesis		FkbB AND FkbC AND FkbA AND FkbP	IRR	fkbB AND fkbC AND fkbA AND fkbP	1 DHCHCACP + 5 SMMCOA + 2 MALCOA + 1 ETHYLMCOA + 2 METHOXYMACPJ + 1 PIPECOLATE + 12 NADPH -> 1 PREFK520 + 12 NADP + 1 ACP + 2 ACPJ + 10 CO2 + 8 H2O
703	FK520 biosynthesis		FkbD (Cytochrome P450) AND FkbM (SAM-dependent methyltransferase)	IRR	fkbD AND fkbM	1 PREFK520 + 1 SAMET + 2 NADPH + 2 O2 + 2 H -> 1 FK520 + 1 SAH + 2 NADP + 2 H2O
704	Biomass biosynthesis	2.7.7.7	DNA synthesis (lumped reaction)	IRR		0.461 DATP + 1.157 DCTP + 0.461 DTTP + 1.157 DGTP + 4.4 ATP -> 4.4 ADP + 4.4 PI + 3.236 PPI + 1 DNA
705	Biomass biosynthesis	NA	peptidoglycan synthesis (lumped reaction)	IRR		1.007 UDPNAM + 1.197 UDPNAG + 1.900 ALAALA + 0.950 ALA + 1.140 MDAPIM + 1.014 GLU + 0.973 GLY + 5.026 ATP -> 1 PEPTIDOGLYCAN + 0.950 DALAxt + 1.197 UDP + 1.007 UMP + 5.026 ADP + 5.026 PI
706	Biomass biosynthesis	NA	phospholipids biosynthesis (artificial reaction)	IRR		1.086 PE + 0.218 PG + 0.052 CDL -> 1 PHOSPHOLIPID
707	Biomass biosynthesis	NA	carbohydrate biosynthesis	IRR		1.897 UDPNAG + 3.794 UDPGAL -> 5.691 UDP + 1 CARBOHYDRATE
708	Biomass biosynthesis	NA	protein synthesis (lumped reaction)	IRR		1.508 ALA + 0.256 ARG + 0.445 ASN + 0.448 ASP + 0.196 CYS + 0.612 GLN + 0.345 GLT + 1.378 GLY + 0.194 HIS + 0.603 ILE + 0.339 LEU + 0.173 LYS + 0.301 MET + 0.328 PHE + 0.916 PRO + 0.518 SER + 0.658 THR + 0.097 TRP + 0.159 TYR + 0.539 VAL + 40.0 ATP -> 40.0 ADP + 40.0 PI + 1 PROTEIN
709	Biomass biosynthesis	2.7.7.6	RNA synthesis (lumped reaction)	IRR		0.600 ATP + 0.826 GTP + 1.031 CTP + 0.662 UTP + 1.25 ATP -> 1.25 ADP + 1.25 PI + 1 RNA + 3.119 PPI
710	Biomass biosynthesis	NA	small molecules pool (artificial reaction)	IRR		0.188 NAD + 0.168 NADP + 0.163 COA + 0.012 ACP + 0.146 MK + 0.281 THF + 0.274 FMN + 0.159 FAD -> 1 SMALLMOLECULES
711	Biomass biosynthesis	NA	triacylglycerol (TAG) synthesis	IRR		1.244 GL3P + 0.050 C140ACP + 1.677 C150ACP + 0.421 C160ACP + 1.570 C170ACP + 0.014 C181ACP -> 1 TAG + 3.732 ACP + 1.244 PI

712	Biomass biosynthesis	NA	teichoic acid biosynthesis	IRR		0.518 POLYGP + 0.129 LYS + 0.129 UDPNAG + 0.129 ATP -> 1 TEICH + 0.129 UDP + 0.129 ADP + 0.129 PI
713	Biomass biosynthesis	NA	BIOMASS SYNTHESIS	IRR		0.446 PROTEIN + 0.163 RNA + 0.042 DNA + 0.027 PHOSPHOLIPID + 0.018 TAG + 0.03 SMALLMOLECULES + 0.137 PEPTIDOGLYCAN + 0.055 CARBOHYDRATE + 0.082 TEICH + 47 ATP -> 47 ADP + 47 PI
ACALinout	Transport Reaction	NA	acetaldehyde transport via facilitated diffusion	REV		1 ACALxt -> 1 ACAL
ACinout	Transport Reaction	NA	acetate transport via proton symport	REV		1 ACxt + 1 Hxt -> 1 AC + 1 H
ADinout	Transport Reaction	NA	adenine transport via proton symport	REV		1 ADxt + 1 Hxt -> 1 AD + 1 H
ADNinout	Transport Reaction	NA	adenosine transport via proton symport	REV		1 ADNxt + 1 Hxt -> 1 ADN + 1 H
AKGinout	Transport Reaction	NA	2-oxoglutarate transport via proton symport	REV		1 AKGxt + 1 Hxt -> 1 AKG + 1 H
ALAin	Transport Reaction	NA	L-alanine transport via ABC system	IRR		1 ALAxt + 1 ATP + 1 H2O -> 1 ALA + 1 ADP + 1 PI
ANout	Transport Reaction	NA	anthranilate transport via facilitated diffusion	IRR		1 AN -> 1 ANxt
ARGin	Transport Reaction	NA	L-arginine transport via ABC system	IRR		1 ARGxt + 1 ATP + 1 H2O -> 1 ARG + 1 ADP + 1 PI
ARGinORNout	Transport Reaction	NA	arginine/ornithine antiporter	REV		1 ARGxt + 1 ORN -> 1 ORNxt + 1 ARG
ASNin	Transport Reaction	NA	L-asparagine transport via ABC system/putative L-asparagine permease	IRR		1 ASNxt + 1 ATP + 1 H2O -> 1 ASN + 1 ADP + 1 PI

ASPin1	Transport Reaction	NA	L-aspartate transport via ABC system	IRR		1 ASPxt + 1 ATP + 1 H2O -> 1 ASP + 1 ADP + 1 PI
ASPin2	Transport Reaction	NA	L-aspartate transport in via proton symport	IRR		1 ASPxt + 1 Hxt -> 1 ASP + 1 H
ASPin3	Transport Reaction	NA	L-aspartate transport via proton symport (2 H)	REV		1 ASPxt + 2 Hxt -> 1 ASP + 2 H
ASPin4	Transport Reaction	NA	L-asparate transport via proton symport (3 H)	IRR		1 ASPxt + 3 Hxt -> 1 ASP + 3 H
CELBin	Transport Reaction	NA	cellobiose transport via ABC system/putative cellobiose transport permease	IRR		1 CELBxt + 1 ATP + 1 H2O -> 1 CELB + 1 ADP + 1 PI
CHOLINEin	Transport Reaction	NA	choline transport via ABC system	IRR		1 CHOLINExt + 1 ATP + 1 H2O -> 1 CHOLINE + 1 ADP + 1 PI
CHOLINEinout	Transport Reaction	NA	choline transport via proton symport	REV		1 CHOLINExt + 1 Hxt -> 1 CHOLINE + 1 H
CITinout	Transport Reaction	NA	citrate transport via protone symport	REV		1 CITxt + 3 Hxt -> 1 CIT + 3 H
CITinSUCCout	Transport Reaction	NA	citrate transport via succinate antiport	REV		1 CITxt + 1 SUCC -> 1 CIT + 1 SUCCxt
CITRinout	Transport Reaction	NA	citrulline transport via facilitated diffusion	REV		1 CITR -> 1 CITRxt
CO2inout	Transport Reaction	NA	CO2 transport via diffusion	REV		1 CO2xt -> 1 CO2
CYSin	Transport Reaction	NA	L-cysteine transport via ABC system	IRR		1 CYSxt + 1 ATP + 1 H2O -> 1 CYS + 1 ADP + 1 PI
CYTDino	Transport	NA	cytidine transport via proton symport	REV		1 CYTDxt + 1 Hxt -> 1 CYTD + 1 H

ut	Reaction					
CYTSinout	Transport Reaction	NA	cytosine transport via proton symport/putative cytosine permease	REV		1 CYTSxt + 1 Hxt -> 1 CYTS + 1 H
DAinout	Transport Reaction	NA	deoxyadenosine transport via proton symport	REV		1 DAxt + 1 Hxt -> 1 DA + 1 H
DALAinout	Transport Reaction	NA	D-alanine transport via proton symport	REV		1 DALAxt + 1 Hxt -> 1 DALA + 1 H
DAPIMin	Transport Reaction	NA	2,6-diaminopimelic acid transport via ABC system	IRR		1 DAPIMxt + 1 ATP + 1 H2O -> 1 DAPIM + 1 ADP + 1 PI
DARABINOSEin1	Transport Reaction	NA	D-arabinose transport via ABC system	IRR		1 DARABINOSExt + 1 ATP + 1 H2O -> 1 DARABINOSE + 1 ADP + 1 PI
DARABITOLin	Transport Reaction	NA	D-arabitol transport via ABC system	IRR		1 DARABITOLxt + 1 ATP + 1 H2O -> 1 DARABITOL + 1 ADP + 1 PI
DCinout	Transport Reaction	NA	deoxycytidine transport via proton symport	REV		1 DCxt + 1 Hxt -> 1 DC + 1 H
DGinout	Transport Reaction	NA	deoxyguanosine transport via proton symport	REV		1 DGxt + 1 Hxt -> 1 DG + 1 H
DGLYCERATEna	Transport Reaction	NA	D-glycerate transport via sodium symport	IRR		1 DGLYCERATExt + 1 Naxt -> 1 DGLYCERATE + 1 Na
DINinout	Transport Reaction	NA	deoxyinosine transport via proton symport	REV		1 DINxt + 1 Hxt -> 1 DIN + 1 H
DLYXOSEin	Transport Reaction	NA	D-lyxose transport via ABC system	IRR		1 DLYXOSExt + 1 ATP + 1 H2O -> 1 DLYXOSE + 1 ADP + 1 PI
DSERinout	Transport Reaction	NA	D-serine transport via proton symport	REV		1 DSERxt + 1 Hxt -> 1 DSER + 1 H

DTinout	Transport Reaction	NA	thymidine transport via proton symport	REV		1 DTxt + 1 Hxt -> 1 DT + 1 H
DUinout	Transport Reaction	NA	deoxyuridine transport via proton symport	REV		1 DUxt + 1 Hxt -> 1 DU + 1 H
ETHinout	Transport Reaction	NA	ethanol transport via proton symport	REV		1 ETHxt + 1 Hxt -> 1 ETH + 1 H
FANinout	Transport Reaction	NA	formyl-anthranilate transport via facilitated diffusion	REV		1 FANxt -> 1 FAN
FEin	Transport Reaction	NA	iron transport via ABC system	IRR		1 FExt + 1 ATP + 1 H2O -> 1 FE + 1 ADP + 1 PI
FK506out	Transport Reaction	NA	FK506 efflux via diffusion	IRR		1 FK506 -> 1 FK506xt
FK506Dout	Transport Reaction	NA	FK506D efflux via diffusion	IRR		1 FK506D -> 1 FK506Dxt
FK520out	Transport Reaction	NA	FK520 efflux via diffusion	IRR		1 FK520 -> 1 FK520xt
FORinout	Transport Reaction	NA	formate transport via diffusion	REV		1 FORxt -> 1 FOR
FRUin1	Transport Reaction	2.7.1.69	fructose transport via PEP:PTS/putative fructose-specific permease	IRR		1 FRUxt + 1 PEP -> 1 PYR + 1 F6P
FRUin2	Transport Reaction	2.7.1.69	fructose transport via PEP:PTS	IRR		1 FRUxt + 1 PEP -> 1 PYR + 1 F1P
FUMin1	Transport Reaction	NA	fumarate transport via proton symport (2 H)	IRR		1 FUMxt + 2 Hxt -> 1 FUM + 2 H
FUMin2	Transport Reaction	NA	fumarate transport via proton symport (3 H)	IRR		1 FUMxt + 3 Hxt -> 1 FUM + 3 H

FUMinSUCCout	Transport Reaction	NA	succinate/fumarate antiporter	REV		1 FUMxt + 1 SUCC -> 1 FUM + 1 SUCCxt
GABAinout	Transport Reaction	NA	4-aminobutyrate transport via proton symport	REV		1 GABAxt + 1 Hxt -> 1 GABA + 1 H
GL3Pin	Transport Reaction	NA	glycerol-3-phosphate/phosphate antiporter	IRR		1 GL3Pxt + 1 ATP + 1 H2O -> 1 GL3P + 1 PI + 1 ADP
GL3PinPIout	Transport Reaction	NA	glycerol-3-phosphate/phosphate antiporter	REV		1 GL3Pxt + 1 PI -> 1 GL3P + 1 PIxt
GLACin	Transport Reaction	NA	galactose transport via ABC system	IRR		1 GLACxt + 1 ATP + 1 H2O -> 1 GLAC + 1 ADP + 1 PI
GLACinH	Transport Reaction	NA	D-galactose transport in via proton symport	IRR		1 GLACxt + 1 Hxt -> 1 GLAC + 1 H
GLAMin	Transport Reaction	2.7.1.69	D-glucosamine transport via PEP:PTS	IRR		1 GLAMxt + 1 PEP -> 1 PYR + 1 GA6P
GLCin	Transport Reaction	2.7.1.69	glucose transport via PEP:PTS	IRR		1 GLCxt + 1 PEP -> 1 G6P + 1 PYR
GLCinH	Transport Reaction	NA	D-glucose uptake via proton symport	IRR		1 GLCxt + 1 Hxt -> 1 GLC + 1 H
GLinout	Transport Reaction	NA	glycerol transport via channel/putative glycerol uptake facilitator protein	REV	glpF	1 GLxt -> 1 GL
GLNin	Transport Reaction	NA	L-glutamine transport via ABC system	IRR		1 GLNxt + 1 ATP + 1 H2O -> 1 GLN + 1 ADP + 1 PI
GLTin	Transport Reaction	NA	glutamate uptake system ATP-binding protein	IRR	gluA	1 GLTxt + 1 ATP + 1 H2O -> 1 GLT + 1 ADP + 1 PI
GLTna	Transport Reaction	NA	Glutamate transport via sodium symport	IRR		1 Naxt + 1 GLTxt -> 1 Na + 1 GLT

GLTout	Transport Reaction	NA	L-glutamate efflux via proton symport	IRR		1 GLT + 1 H -> 1 GLTxt + 1 Hxt
GLUCinout	Transport Reaction	NA	D-gluconate transport via proton symport/putative gluconate permease	REV		1 GLUCxt + 1 Hxt -> 1 GLUC + 1 H
GLYCOLATEinout	Transport Reaction	NA	glycolate transport via diffusion	REV		1 GLYCOLATExt -> 1 GLYCOLATE
GLYCOLATEinoutH	Transport Reaction	NA	glycolate transport via proton transport	REV		1 GLYCOLATExt + 1 Hxt -> 1 GLYCOLATE + 1 H
GLYin	Transport Reaction	NA	glycine transport via proton symport	IRR		1 GLYxt + 1 ATP + 1 H2O -> 1 GLY + 1 ADP + 1 PI
GLYinout	Transport Reaction	NA	glycine transport via proton symport	REV		1 GLYxt + 1 Hxt -> 1 GLY + 1 H
GNinout	Transport Reaction	NA	guanine transport via facilitated diffusion	REV		1 GNxt -> 1 GN
GSNinout	Transport Reaction	NA	guanosine transport via proton symport	REV		1 GSNxt + 1 Hxt -> 1 GSN + 1 H
h2	Transport Reaction	NA	proton transport	IRR		1 H -> 1 Hxt
HISin1	Transport Reaction	NA	L-histidine transport via ABC system	IRR		1 HISxt + 1 ATP + 1 H2O -> 1 HIS + 1 ADP + 1 PI
HISinout	Transport Reaction	NA	L-histidine transport via proton symport	REV		1 HISxt + 1 Hxt -> 1 HIS + 1 H
HMPinout	Transport Reaction	NA	2-(Hydroxymethyl)phenol transport via diffusion	REV		1 HMPxt -> 1 HMP

HXMPt	Transport Reaction	NA	2-(hydroxymethyl)phenol transport in/out via proton symport	REV		1 HXMPxt + 1 Hxt -> 1 HXMP + 1 H
HYXNinout	Transport Reaction	NA	hypoxanthine transport via facilitated diffusion	REV		1 HYXNxt -> 1 HYXN
ILEin1	Transport Reaction	NA	L-isoleucine transport via ABC system	IRR		1 ILExt + 1 ATP + 1 H2O -> 1 ILE + 1 ADP + 1 PI
ILEinout	Transport Reaction	NA	L-isoleucine transport via proton symport	REV		1 ILExt + 1 Hxt -> 1 ILE + 1 H
INDOLEinout	Transport Reaction	NA	Iindole transport via proton transport	REV		1 INDOLExt + 1 Hxt -> 1 INDOLE + 1 H
INSinout	Transport Reaction	NA	inosine transport via proton symport	REV		1 INSxt + 1 Hxt -> 1 INS + 1 H
KDGinout	Transport Reaction	NA	2-dehydro-3-deoxy-D-gluconate transport via proton symport	REV		1 KDGxt + 1 Hxt -> 1 KDG + 1 H
Kinout	Transport Reaction	NA	potasium transport via proton transport	REV		1 Kxt + 1 Hxt -> 1 K + 1 H
LACinout	Transport Reaction	NA	D-lactate transport via proton symport	REV		1 LACxt + 1 Hxt -> 1 LAC + 1 H
LACTOSEin	Transport Reaction	NA	lactose transport via ABC system	IRR		1 LACTOSExt + 1 ATP + 1 H2O -> 1 LACTOSE + 1 ADP + 1 PI
LARABINOSEin1	Transport Reaction	NA	L-arabinose transport via ABC system	IRR		1 LARABINOSExt + 1 ATP + 1 H2O -> 1 LARABINOSE + 1 ADP + 1 PI
LARABINOSEin2	Transport Reaction	NA	L-arabinose transport via proton symport	IRR		1 LARABINOSExt + 1 Hxt -> 1 LARABINOSE + 1 H
LEUin1	Transport Reaction	NA	L-leucine transport via ABC system	IRR		1 LEUxt + 1 ATP + 1 H2O -> 1 LEU + 1 ADP + 1 PI

LEUinout	Transport Reaction	NA	L-leucine transport via proton symport	REV		1 LEUxt + 1 Hxt -> 1 LEU + 1 H
LLACinout	Transport Reaction	NA	L-Lactate metabolism transport via proton symport/putative L-Lactate metabolism permease	REV		1 LLACxt + 1 Hxt -> 1 LLAC + 1 H
LLYXOSEin	Transport Reaction	NA	L-lyxose transport via ABC system	IRR		1 LLYXOSExt + 1 ATP + 1 H2O -> 1 LLYXOSE + 1 ADP + 1 PI
LYSin1	Transport Reaction	NA	L-lysine transport via ABC system	IRR		1 LYSxt + 1 ATP + 1 H2O -> 1 LYS + 1 ADP + 1 PI
LYSinCADAout	Transport Reaction	NA	lysine/cadaverine antiporter	REV		1 CADA + 1 Hxt + 1 LYSxt -> 1 CADAxt + 1 LYS + 1 H
LYSinout	Transport Reaction	NA	L-lysine reversible transport via proton symport	REV		1 LYSxt + 1 Hxt -> 1 LYS + 1 H
MALin1	Transport Reaction	NA	malate transport via proton symport (2 H)	IRR		1 MALxt + 2 Hxt -> 1 MAL + 2 H
MALin2	Transport Reaction	NA	malate transport via proton symport (3 H)	IRR		1 MALxt + 3 Hxt -> 1 MAL + 3 H
MANin	Transport Reaction	2.7.1.69	D-mannose transport via PEP:PTS	IRR		1 MANxt + 1 PEP -> 1 PYR + 1 MAN6P
MANNITOLin	Transport Reaction	2.7.1.69	D-mannitol transport via PEP:PTS	IRR		1 MANNITOLxt + 1 PEP -> 1 MANNITOL1P + 1 PYR
MELIinout	Transport Reaction	NA	melibiose transport via proton symport	REV		1 MELIxt + 1 Hxt -> 1 MELI + 1 H
METin	Transport Reaction	NA	L-methionine transport via ABC system	IRR		1 METxt + 1 ATP + 1 H2O -> 1 MET + 1 ADP + 1 PI
MLTin	Transport	2.7.1.69	Maltose transport via PEP:PTS	IRR		1 MLTxt + 1 PEP -> 1 PYR + 1 MLT6P

	Reaction					
MLTinATP	Transport Reaction	NA	maltose transport via ABC system/putative maltose-binding protein	IRR	malE	1 MLTxt + 1 ATP + 1 H2O -> 1 MLT + 1 ADP + 1 PI
NACinout	Transport Reaction	NA	nicotinic acid transport via facilitated diffusion	REV		1 NACxt -> 1 NAC
Naout1	Transport Reaction	NA	sodium proton antiporter (H:NA is 1:1)/putative sodium:solute symporter	IRR		1 Hxt + 1 Na -> 1 Naxt + 1 H
Naout2	Transport Reaction	NA	sodium proton antiporter (H:NA is 2)/putative sodium:solute symporter	IRR		2 Hxt + 1 Na -> 1 Naxt + 2 H
Naout3	Transport Reaction	NA	sodium proton antiporter (H:NA is 1.5)/putative sodium:solute symporter	IRR		3 Hxt + 2 Na -> 1 Naxt + 3 H
NH3inout	Transport Reaction	NA	ammonia transport via diffusion	REV		1 NH3xt -> 1 NH3
NO2inout	Transport Reaction	NA	nitrite transport via proton transport	REV		1 NO2xt + 1 Hxt -> 1 NO2 + 1 H
NO3inout	Transport Reaction	NA	nitrate transport via diffusion	REV		1 NO3xt -> 1 NO3
O2inout	Transport Reaction	NA	oxygen transport via diffusion	REV		1 O2xt -> 1 O2
ORNin	Transport Reaction	NA	ornithine transport via ABC system	IRR		1 ORNxt + 1 ATP + 1 H2O -> 1 ORN + 1 ADP + 1 PI
PACout	Transport Reaction	NA	phenylacetic acid transport via facilitated diffusion	IRR		1 PAC -> 1 PACxt
PHEinout	Transport Reaction	NA	L-phenylalanine transport via proton symport	REV		1 PHExt + 1 Hxt -> 1 PHE + 1 H

PIin	Transport Reaction	NA	phosphate transport via ABC system/phosphate-binding protein precursor	IRR	pstS	1 PIxt + 1 ATP + 1 H2O -> 2 PI + 1 ADP
PIinout	Transport Reaction	NA	phosphate reversible transport via symport/ phosphate transport protein	REV	pitH	1 PIxt + 1 Hxt -> 1 PI + 1 H
PROin1	Transport Reaction	NA	L-proline transport via ABC system	IRR		1 PROxt + 1 ATP + 1 H2O -> 1 PRO + 1 ADP + 1 PI
PROinout	Transport Reaction	NA	L-proline transport via proton symport	REV		1 PROxt + 1 Hxt -> 1 PRO + 1 H
PROna	Transport Reaction	NA	L-proline transport via sodium symport	IRR		1 Naxt + 1 PROxt -> 1 Na + 1 PRO
PROPANOATEinout	Transport Reaction	NA	propanoate transport via proton symport	REV		1 PROPANOATExt + 1 Hxt -> 1 PROPANOATE + 1 H
PYRinout	Transport Reaction	NA	pyruvate transport via proton symport	REV		1 PYRxt + 1 Hxt -> 1 PYR + 1 H
REDout	Transport Reaction	NA	RED efflux via diffusion	IRR		1 PYRAPCP -> 1 PYRAPCPxt
RIBin	Transport Reaction	NA	D-ribose transport via ABC system	IRR		1 RIBxt + 1 ATP + 1 H2O -> 1 RIB + 1 ADP + 1 PI
RMNin	Transport Reaction	NA	potasium transport via proton transport	IRR		1 RMNxt + 1 Hxt -> 1 RMN + 1 H
SALICINin	Transport Reaction	2.7.1.69	salicin transport via PEP:PTS	IRR		1 SALICINxt + 1 PEP -> 1 SAL6P + 1 PYR
SERinout	Transport Reaction	NA	L-serine transport via proton symport	REV		1 SERxt + 1 Hxt -> 1 SER + 1 H

SERna	Transport Reaction	NA	L-serine transport via sodium symport	IRR		1 Naxt + 1 SERxt -> 1 Na + 1 SER
SLFin	Transport Reaction	NA	sulfate transport via ABC system	IRR		1 SLFxt + 1 ATP + 1 H2O -> 1 SLF + 1 ADP + 1 PI
SOTin	Transport Reaction	2.7.1.69	D-sorbitol transport via PEP:PTS	IRR		1 SOTxt + 1 PEP -> 1 SOT + 1 PYR
SPERMIDINEin	Transport Reaction	NA	SPERMIDINE transport via ABC system	IRR		1 SPERMIDINExt + 1 ATP + 1 H2O -> 1 SPERMIDINE + 1 ADP + 1 PI
STARCHinH	Transport Reaction	NA	Starch uptake via proton symport	IRR		1 STARCHnxt + 1 Hxt -> 1 STARCH + 1 H
SUCCin1	Transport Reaction	NA	succinate transport via proton symport (2 H)	IRR		1 SUCCxt + 2 Hxt -> 1 SUCC + 2 H
SUCCin2	Transport Reaction	NA	succintate transport via proton symport (3 H)	IRR		1 SUCCxt + 3 Hxt-> 1 SUCC + 3 H
SUCCout	Transport Reaction	NA	succinate efflux via proton symport	IRR		1 SUCC + 1 H -> 1 SUCCxt + 1 Hxt
SUCROSEin	Transport Reaction	NA	sucrose transport via ABC system	IRR		1 SUCROSExt + 1 ATP + 1 H2O -> 1 SUCROSE + 1 ADP + 1 PI
T3inout	Transport Reaction	NA	glyceraldehyde transport via facilitated diffusion	REV		1 T3xt -> 1 T3
TARTRATEinSUCCout	Transport Reaction	NA	tartrate/succinate antiporter	REV		1 TARTRATExt + 1 SUCC -> 1 SUCCxt + 1 TARTRATE
THRin1	Transport Reaction	NA	L-threonine transport via ABC system	IRR		1 THRxt + 1 ATP + 1 H2O -> 1 THR + 1 ADP + 1 PI

THRin2	Transport Reaction	NA	L-threonine transport via proton symport	REV		1 THRxt + 1 Hxt -> 1 THR + 1 H
THRna	Transport Reaction	NA	L-threonine transport via sodium symport	IRR		1 Naxt + 1 THRxt -> 1 Na + 1 THR
THYin	Transport Reaction	NA	THY transport via ABC system	IRR		1 THYxt + 1 ATP + 1 H2O -> 1 THY + 1 ADP + 1 PI
TREin	Transport Reaction	2.7.1.69	trehalose transport via PEP:PTS	IRR		1 TRExt + 1 PEP -> 1 TRE6P + 1 PYR
TRPinout	Transport Reaction	NA	L-tryptophan transport via proton symport	REV		1 TRPxt + 1 Hxt -> 1 TRP + 1 H
TYRinout	Transport Reaction	NA	L-tyrosine transport via proton symport	REV		1 TYRxt + 1 Hxt -> 1 TYR + 1 H
UDPNAGin	Transport Reaction	2.7.1.69	N-actetyl glocoseamine transport via PEP:PTS	IRR		1 UDPNAGxt + 1 PEP -> 1 PYR + 1 NADGLUCOSA6P
URAinout	Transport Reaction	NA	uracil transport via proton symport	REV		1 URAxt + 1 Hxt -> 1 URA + 1 H
UREAinout	Transport Reaction	NA	urea transport via facilitate diffusion	REV		1 UREAxt -> 1 UREA
URIinout	Transport Reaction	NA	uridine transport via proton symport	REV		1 URIxt + 1 Hxt -> 1 URI + 1 H
VALin1	Transport Reaction	NA	L-valine transport via ABC system	IRR		1 VALxt + 1 ATP + 1 H2O -> 1 VAL + 1 ADP + 1 PI
VALinout	Transport Reaction	NA	L-valine transport via proton symport	REV		1 VALxt + 1 Hxt -> 1 VAL + 1 H
H2Oin	Transport Reaction	NA	H2O transport	IRR		1 H2O -> 1 H2Oxt

XANinout	Transport Reaction	NA	xanthine transport via facilitated diffusion	REV		1 XANxt -> 1 XAN
XYLin1	Transport Reaction	NA	D-xylose transport via ABC system	IRR		1 XYLxt + 1 ATP + 1 H2O -> 1 XYL + 1 ADP + 1 PI
XYLin2	Transport Reaction	NA	D-xylose transport via proton symport	IRR		1 XYLxt + 1 Hxt -> 1 XYL + 1 H
XYLITOLin	Transport Reaction	NA	xylitol transport via ABC system	IRR		1 XYLITOLxt + 1 ATP + 1 H2O -> 1 XYLITOL + 1 ADP + 1 PI

Detailed metabolites list

Abbreviation	Metabolite
2MBCOA	(S)-2-Methylbutanoyl-CoA
2MBUTLIPO	(S)-2-Methylbutanoyldihydrolipoamide
2PCDPMDE	4-diphosphocytidyl-2-C-methylerythritol 2-phosphate
2PG	2-phosphoglycerate
3MBCOA	3-Methylbutanoyl-CoA
3MBUTLIPO	(S)-3-Methylbutanoyldihydrolipoamide
3PG	3-phosphoglycerate
3PSME	5-enolpyruvyl-shikimate-3-phosphate
A5LEVULINATE	5-Aminolevulinate
A6RP	5-amino-6-ribitylamino-2,4(1H,3H)-pyrimidinedione
A6RP5P	5-amino-6-(5'-phosphoribosylamino)uracil
A6RP5P2	5-amino-6-(5'-phosphoribitylamino)uracil
A8OXO7NONANOATE	8-amino-7-oxononanoate
AACCOA	acetoacetyl-CoA
AACETONE	aminoacetone
AC	acetate
ACACDIM	adenosyl-cobyrinic acid a,c-diamide
ACACP	Acyl-[acyl-carrier protein]
ACAL	acetaldehyde
ACARB	Apo-[carboxylase]
ACCOA	acetyl-CoA
ACETYLEP	acetylphosphate
ACOBALAMIN	coenzyme B2
ACOBINAMIDE	adenosylcobinamide
ACOBINAMIDEGDP	adenosylcobinamide-GDP
ACOBINAMIDEP	adenosylcobinamide-P
ACOBYRATE	adenosyl-cobyrate
ACP	Acyl-carrier protein
ACPJ	Acyl-carrier protein (FkbJ)
ACRCOA	Acryloyl-CoA
ACTAC	acetoacetate
AD	adenine
ADCHOR	4-amino-4-deoxychorismate
ADENYLOSUCC	adenylo-succinate
ADHAP	Dihydroxyacetone phosphate
ADLIPO	6-S-Acetyldihydrolipoamide
ADN	adenosine
ADP	ADP
AGL3P	1-Acyl-sn-glycerol 3-phosphate
AGMATINE	agmatine

AHBUT	2-aceto-2-hydroxy-butyrate
AHHMD	2-amino-4-hydroxy-6-hydroxymethyl-7,8-dihydropteridine diphosphate
AHHMP	6-hydroxymethyl-dehydropterin
AHMMPYRP	hydroxymethylpyrimidine phosphate
AHMMPYRPP	4-amino-5-hydroxymethyl-2-methylpyrimidine-pyrophosphate
AHTD	dihydroneopterin triphosphate
AIC	5-Amino-4-imidazolecarboxyamide
AICAR	1-(5'-Phosphoribosyl)-5-amino-4-imidazolecarboxamide
AIR	5-aminoimidazole ribonucleotide
AKG	α-ketoglutarate
ALA	L-alanine
ALAALA	D-alanyl-D-alanine
ALAC	2-acetolactate
ALLYLMCOA	Allylmalonyl-CoA
AMINOVAL	5-Aminovaleric acid
AMP	AMP
AN	anthranilate
AOXOBUT	2-amino-3-oxobutanoate
APROPANOL	1-amino-propan-2-ol
APS	Adenylylsulfate
ARABITOL	L-Arabitol
ARG	L-arginine
ARGSUCC	L-arginino-succinate
ARIBAZOLE	α-ribazole
ARIBAZOLE5P	α-ribazole-5'-P
ASER	O-acetyl-L-serine
ASN	L-asparagine
ASP	L-aspartate
ASPSA	L-aspartate-semialdehyde
ATN	allantoin
ATP	ATP
ATT	allantoate
B5AMP	Biotinyl-5'-AMP
BALA	β-alanine
BASP	L-aspartyl-4-phosphate
BCCP	Holo-[carboxylase]
BETAINE	glycine betaine
BETAINEALD	betaine aldehyde
bG6P	β-D-glucose-6-phosphate
bGLC	β-D-glucose
BIOTIN	biotin
C040COA	Butanoyl-CoA
C060COA	Hexanoyl-CoA

C080COA	Octanoyl-CoA
C100COA	Decanoyl-CoA
C120COA	dodecanoyl-CoA
C140ACP	Tetradecanoyl-[acyl-carrier protein]
C140COA	Tetradecanoyl-CoA
C150ACP	Pentadecanoyl-[acyl-carrier protein]
C160ACP	Hexadecanoyl-[acyl-carrier protein]
C160COA	Hexadecanoyl-CoA
C170ACP	Heptadecanoyl-[acyl-carrier protein]
C170COA	Heptadecanoyl-[acyl-carrier protein]
C181ACP	Oleoyl-[acyl-carrier protein]
C2DOF5ACET	2-carboxy-2,5-dihydro-5-oxofuran-2-acetate
C3MUCO	3-carboxy-cis,cis-muconate
CAACDIM1	cob(I)yrinic acid a,c-diamide
CAACDIM2	cob(II)yrinic acid a,c-diamide
CAASP	carbamoyl-L-aspartate
CADA	Cadaverine
CAIR	4-carboxyaminoimidazole ribonucleotide
CAMP	cyclic-AMP
CAP	carbamoyl-phosphate
CARBOHYDRATE	CARBOHYDRATE (Biomass component)
CATECHOL	catechol
CCCP	Carboxybiotin-carboxyl-carrier protein
CDL	cardiolipin
CDP	CDP
CDPDIACYLGLYCEROL	CDP-diacylglycerol
CDPGL	CDP-glycerol
CDPMDE	4-diphosphocytidyl-2-C-methylerythritol
CELB	cellobiose
CGMP	cGMP
CH33ADO	5'-Deoxyadenosine
CHEXANOYLCOA	pimeloyl-CoA
CHISOCARPOATE	3-carboxy-3-hydroxy-isocaproate
CHOLINE	choline
CHOR	chorismate
CIT	citrate
CITR	citrulline
CMO	3-Carboxy-4-methyl-2-oxopentanoate
CMP	CMP
CO2	CO2
COA	coenzyme A
COBALT	Cobalt
COPROPORPHYRINOGEN	coproporphyrinogen

CPAD5P	1-(o-carboxyphenylamino)-1'-deoxyribulose-5'-phosphate
CROTCOA	crotonyl-CoA
CTP	CTP
CYS	L-cysteine
CYSGLY	Cys-Gly
CYSTATHIONINE	cystathionine
CYTD	cytidine
CYTOSINE	Cytosine (in DNA)
CYTS	cytosine
D1PDICARBOXYLATE	L-2,3,4,5-tetrahydrodipicolinate
D6PGC	6-Phospho-D-gluconate
D6PGL	D-Glucono-1,5-lactone 6-phosphate
D6RP5P	2,5-diamino-6-(ribosylamino)-4-(3H)-pyrimidinone 5'-phosphate
D8RL	6,7-dimethyl-8-(1-D-ribityl)lumazine
DA	deoxyadenosine
DADP	dADP
DAHP	3-deoxy-D-arabino-heptulosonate-7-phosphate
DALA	D-alanine
DAMP	Deoxyadenosine monophosphate
DAPIM	L,L-diaminopimelate
DARABINOSE	D-arabinose
DARABITOL	D-arabitol
DATP	dATP
DBENZIMIDAZOLE	dimethylbenzimidazole
DC	deoxycytidine
DCDC	(4R;5R)-4,5-dihydroxycyclohexa-1,5-dienecarboxylic acid
DCDP	dCDP
DCMP	dCMP
DCTP	dCTP
DDCOL	L-2,3-dihydrodipicolinate
DDG	2-Deoxy-D-gluconate
DDLGCVH	Dihydrolipoylprotein
DDPAN	2-dehydropantoate
DECANOATE	DECANOATE
DETHIOBIOTIN	dethiobiotin
DG	deoxyguanosine
DGA	5-Deoxy glucuronic acid
DGDP	dGDP
DGLYCERATE	glycerate
DGMP	dGMP
DGTP	dGTP
DHAP	dihydroxy-acetone-phosphate
DHCHC	(4R;5R)-4,5-dihydroxycyclohex-1-enecarboxylic acid

DHCHCACP	(4R;5R)-4,5-dihydroxycyclohexanecarboxylic acid-ACP
DHF	7,8-dihydrofolate
DHN	1,4-Dihydroxy-2-naphthoate
DHP	dihydro-neo-pterin
DHPT	7,8-dihydropteroate
DIAMINONONANOATE	7,8-diaminononanoate
DIBENZOATE	protocatechuate
DIMGP	D-erythro-imidazole-glycerol-phosphate
DIN	deoxyinosine
DIVALER	2,3-dihydroxy-isovalerate
DKDI	D-2,3-Diketo-4-deoxy-epi-inositol
DKH	2-Deoxy-5-keto-D-gluconic acid
DKHP	2-Deoxy-5-keto-D-gluconic acid 6-phosphate
DLIPO	dihydrolipoamide
DLYXOSE	D-Lyxose
DMK	2-Demethylmenaquinone
DMPP	dimethylallyl-pyrophosphate
DNA	DNA (Biomass component)
DODECANOATE	dodecanoate
DOROA	dihydroorotate
DPCOA	dephospho-CoA
DPG	1,3-diphosphateglycerate
DPNTP	2,5-Diaminopyrimidine nucleoside triphosphate
DQT	3-dehydroquinate
DR1P	deoxyribose-1-phosphate
DR5P	deoxyribose-5-phosphate
DRIB	D-Ribose
DSER	D-serine
DSHCLORIN	dihydrosirohydrochlorin
DSHIK	3-dehydro-shikimate
DT	thymidine
DTDP	dTDP
DTMP	DTMP
DTTOAO	2,5-Diamino-6-(5'-triphosphoryl-3',4'-trihydroxy-2'-oxopentyl)- amino-4-oxopyrimidine
DTTP	dTTP
DU	deoxyuridine
DUDP	dUDP
DUMP	dUMP
DUTP	dUTP
DX5P	1-deoxy-D-xylulose 5-phosphate
DXYL	xylulose
E4HG	L-erythro-4-Hydroxyglutamate
E4P	D-erythrose-4-phosphate

ECTOINE	ectoine
EPM	Epimelibiose
ETH	ethanol
ETHYLMCOA	Ethylmalonyl-CoA
F10THF	N10-formyl-THF
F1P	fructose-1-phosphate
F5THF	Folinic acid
F6P	D-fructose-6-phosphate
FACETOACETATE	4-fumaryl-acetoacetate
FAD	FAD
FADH2	FADH2
FAN	Formylanthranilate
FDP	fructose-1,6-bisphosphate
FE	Fe
FERI	Oxidized ferredoxin
FERO	Reduced ferredoxin
FGAM	5-phosphoribosyl-N-formylglycineamidine
FGAR	5'-phosphoribosyl-N-formylglycineamide
FK506	FK506
FK506D	37,38-dihydroFK506
FK520	FK520
FKYN	L-Formylkynurenine
FMN	FMN
FOR	formate
FORMAL	formaldehyde
FORMAMIDE	formamide
FPNTP	Formamidopyrimidine nucleoside triphosphate
FPP	trans, trans-farnesyl diphosphate
FRU	fructose
FUM	fumarate
G1P	D-glucose-1-phosphate
G4D6DMAN	GDP-4-dehydro-6-deoxy-D-mannose
G6P	D-glucose-6-phosphate
GA1P	glucosamine-1P
GA6P	D-glucosamine-6-phosphate
GABA	4-aminobutyrate
GALACTINOL	galactinol
GALC	D-Galactose
GALC1P	α-D-galactose-1-phosphate
GAP	D-glyceraldehyde-3-phosphate
GAR	5-phospho-ribosyl-glycineamide
GDP	GDP
GDPTP	guanosine 3'-diphosphate 5'-triphosphate

GGL	Galactosylglycerol
GGPP	geranylgeranyl-PP
GL	glycerol
GL3P	glycerol-3-phosphate
GLAC	D-galactose
GLAL	glycolaldehyde
GLC	D-glucose
GLCLAC	glucono-δ-lactone
GLN	L-glutamine
GLT	L-glutamate
GLU	D-Glutamate
GLU5P	L-glutamate-5-phosphate
GLUC	gluconate
GLUGSAL	L-glutamate γ-semialdehyde
GLUT	Glutarate
GLUTCOA	Glutaryl-CoA
GLUTSEMI	Glutarate semialdehyde
GLX	glyoxylate
GLY	glycine
GLYCERON	Glycerone
GLYCOLATE	glycolate
GMAN	GDP-mannose
GMP	GMP
GN	guanine
GPP	geranyl-PP
GSH	Glutathione
GSN	guanosine
GSSG	Glutathione disulfide
GTP	GTP
GU5DP3DP	guanosine 5'-diphosphate,3'-diphosphate
H	H
H2CO3	H2CO3
H2O	Water
H2O2	H2O2
H2S	H2S
H2SO3	H2SO3
H3BUTCOA	3-hydroxybutyryl-CoA
HBUTP	3,4-dihydroxy-2-butanone-4-P
HCO3	HCO3
HEME	HEME
HEMEO	Heme O
HEPPP	all-trans-Heptaprenyl diphosphate
HEPTADECANOATE	heptadecanoate

HEXADECANOATE	hexadecanoate
HEXANOATE	hexanoate
HGBYRINATE	hydrogenobyrinate
HGBYRINATED	hydrogenobyrinate a,c-diamide
HIS	L-histidine
HISOBUT	3-hydroxy-isobutyrate
HISOBUTCOA	3-hydroxy-isobutyryl-CoA
HISOL	histidinol
HISOLP	L-histidinol-phosphate
HIU	5-Hydroxyisourate
HKVALERATE	4-hydroxy-2-ketovalerate
HMB4PP	1-hydroxy-2-methyl-2-(E)-butenyl 4-diphosphate
HMGCOA	3-hydroxy-3-methyl-glutaryl-CoA
HMP	hydroxymethylpyrimidine
HOMOCYC	homocysteine
HOMOGENTISATE	homogentisate
HOMOSER	homoserine
HPCOA	3-Hydroxypropanoyl coenzymeA
HPPP	all-trans-Hexaprenyl diphosphate
HPRO	trans-4-Hydroxy-L-proline
HXMP	2-(Hydroxymethyl)phenol
HYDROXYAKG	D-4-Hydroxy-2-oxoglutarate
HYDROXYMETHYLBILANE	hydroxymethylbilane
HYDROXYPYRUVATE	hydroxypyruvate
HYXN	hypoxanthine
HZP	5-(2-Hydroxyethyl)-4-methylthiazole
IBUTLIPO	S-Isobutanoyldihydrolipoamide
ICHOR	Isochorismate
ICIT	isocitrate
IDP	Inosine diphosphate
IGP	indole-3-glycerol-phosphate
ILE	L-isoleucine
IMACP	imidazole acetol-phosphate
IMI5PROP	4-imidazolone-5-propionate
IMP	IMP
INDOLE	indole
INS	inosine
IPP	Δ3-isopentenyl-PP
ISOBUTCOA	isobutyryl-CoA
ISOVALCOA	isovaleryl-CoA
ISUCC	iminoaspartate
ITP	Inosine triphosphate
K	potasium

K2MINOSITOL	2-keto-myo-inositol
KADCOA	β-ketoadipyl-CoA
KADIPATE	β-ketoadipate
KDG	2-dehydro-3-deoxy-D-gluconate
KDPG	2-keto-3-deoxy-6-phospho-gluconate
KISOVALERATE	2-keto-isovalerate
KMV	2,3-dihydroxy-3-methylvalerate
KMVALERATE	2-keto-3-methyl-valerate
KYN	L-Kynurenine
L4HG	L-erythro-4-Hydroxyglutamate
L4HGSA	L-4-Hydroxyglutamate semialdehyde
LAC	D-lactate
LACTAL	L-Lactaldehyde
LACTOSE	lactose
LARABINOSE	L-Arabinose
LEU	L-leucine
LIBUTANOATE	L-2,4-diaminobutanoate
LIPO	lipoamide
LLAC	lactate
LLYXOSE	L-Lyxose
LRIBULOSE	L-Ribulose
LRIBULOSE5P	L-Ribulose 5-phosphate
LXYLULOSE	L-Xylulose
LXYLULOSE5P	L-Xylulose 5-phosphate
LYS	L-lysine
M5CSN	5-Methylcytosine
M5THTGLU	5-methyltetrahydropteroyltri-L-glutamate
MAACOA	2-methylaceto-acetyl-CoA
MACETOACETATE	4-maleyl-acetoacetate
MAL	malate
MALACP	Malonyl-[acyl-carrier protein]
MALCOA	malonyl-CoA
MALONATE	Malonate
MAN	mannose
MAN1P	α-D-mannose 1-phosphate
MAN6P	mannose-6-phosphate
MANNITOL	mannitol
MANNITOL1P	D-Mannitol 1-phosphate
MCCOA	methacrylyl-CoA
MCROTCOA	3-Methylcrotonyl-CoA
MDAPIM	meso-diaminopimelate
MDE4P	2-C-methyl-D-erythritol-4-phosphate
MDECPP	2-C-methyl-D-erythritol-2,4-cyclodiphosphate

MELI	melibiose
MELIT	Melibiitol
MET	L-methionine
METH	Methanol
METHENYLTHF	5,10-methenyl-THF
METHOXYMACPJ	Methoxymalonyl-ACPJ
METHTHF	5,10-methylene-THF
MHBUTCOA	2-methyl-3-hydroxybutyryl-CoA
MINO1P	D-myo-inositol (3)-monophosphate
MK	menaquinone
MKH2	menaquinol
MLT	maltose
MLT6P	Maltose 6'-phosphate
MMAL	Methylmalonate
MMALONATEALD	methylmalonate-semialdehyde
MMCOA	(R)-methylmalonyl-CoA
MPENTANOATE	2-keto-4-methyl-pentanoate
MSALD	Malonate semialdehyde
MTADENOSINE	5'-methylthioadenosine
MTHF	5-methyl-THF
MTRIP	S-Methyl-5-thio-D-ribulose 1-phosphate
MTRP	S-Methyl-5-thio-D-ribose 1-phosphate
MYOINOSITOL	myo-inositol
Na	Na
NAACORN	N-α-acetylornithine
NAAD	deamido-NAD
NAC	nicotinate
NACD	nicotinate nucleoside
NACN	nicotinate nucleotide
NAD	NAD
NADBUT	N-acetyl-L-2,4-diaminobutanoate
NADGLUCOSA6P	N-acetyl-D-glucosamine-6-phosphate
NADH	NADH
NADP	NADP
NADPH	NADPH
NAGA1P	N-acetyl-glucosamine-1-phosphate
NAGLU	N-acetyl-L-glutamate
NAGLUP	N-acetylglutamyl-phosphate
NAGLUS	N-acetylglutamate semialdehyde
NFORMIGLU	N-formimino-L-glutamate
NH3	ammonia
NICOTINAMIDE	nicotinamide
NMN	Nicotinamide nucleotide

NO2	NO2
NO3	NO3
NPP	all-trans-Nonaprenyl diphosphate
NPRAN	N-(5-Phospho-D-ribosyl)anthranilate
O2	O2
OA	oxaloacetate
OAELAC	β-ketoadipate-enol-lactone
OCTANOATE	OCTANOATE
OMP	orotidine-5'-phosphate
OPCOA	3-oxopropionyl-CoA
OPP	all-trans-Octaprenyl diphosphate
ORN	L-ornithine
OROA	orotate
OSB	O-succinylbenzoate
OSBCOA	o-Succinylbenzoyl-CoA
OSLHSER	O-succinyl-L-homoserine
OTHIO	Oxidized thioredoxin
OXOBUTANOATE	2-oxobutanoate
OXOPENTENOATE	2-oxopent-4-enoate
P35C	L-1-Pyrroline-3-hydroxy-5-carboxylate
P3I	PPPi
P5C	1-pyrroline-5-carboxylate
P6GLUCONATE	6-phospho-D-gluconate
PABA	p-aminobenzoate
PAC	phenylacetate
PADNLT	Propinol adenylate
PANT	pantothenate
PANTOATE	L-pantoate
PANTOTHENP	D-4'-phosphopantothenate
PANTP	pantetheine 4'-phosphate
PAP	adenosine 3',5'-bisphosphate
PAPS	3'-Phosphoadenylylsulfate
PE	L-1-phosphatidyl-ethanolamine
PEP	phosphoenolpyruvate
PEPTIDOGLYCAN	PEPTIDOGLYCAN (Biomass componet)
PG	Phosphatidylglycerol
PGP	Phosphatidylglycerophosphate
PHAC	Phenylacetaldehyde
PHE	L-phenylalanine
PHEN	prephenate
PHOSPHATIDATE	Phosphatidate
PHOSPHOLIPID	PHOSPHOLIPID (Biomass component)
PHP	3-phospho-hydroxypyruvate

PHPPYR	p-hydroxyphenylpyruvate
PHPYR	phenylpyruvate
PHSER	O-phospho-L-homoserine
PI	phosphate
PIn	(Phosphate)n
PIn-1	(Phosphate)n-1
PIPECOLATE	Pipecolate
PIPER	Piperideine
PLIPOYLLYSINE	Lipoylprotein
POLYGP	Poly(glycerol phosphate)
PORPHOBILINOGEN	porphobilinogen
PPI	pyrophosphate
PPPP	all-trans-Pentaprenyl diphosphate
PRAM	5-phospho-β-D-ribosyl-amine
PRBAMP	phosphoribosyl-AMP
PRBATP	phosphoribosyl-ATP
PRCN	precorrin-6x
PRECORRIN1	Precorrin 1
PRECORRIN3A	precorrin-3A
PRECORRIN3B	precorrin-3B
PRECORRIN4B	precorrin-4B
PRECORRIN5	precorrin-5
PRECORRIN6B	precorrin-6y
PRECORRIN8X	precorrin-8x
PREFK506	FK506 intermediate
PREFK506D	FK506D intermediate
PREFK520	FK520 intermediate
PRFICA	phosphoribosyl-formamido-carboxamide
PRFP	phosphoribosylformiminoAICAR-phosphate
PRLP	phosphoribulosylformimino-AICAR-P
PRO	L-proline
PROPANOATE	Propanoate
PROPIONYLACP	Propionyl-[acyl-carrier protein]
PROPIONYLCOA	propionyl-CoA
PROPIONYLP	propionyl-P
PROPYLMCOA	Propylmalonyl-CoA
PROTEIN	Protein (biomass component)
PROTOHEME	protoheme
PROTOPORPHYRIN	protoporphyrin
PROTOPORPHYRINOGEN	Protoporphyrinogen IX
PRPP	PRPP
PSER	L-1-phosphatidyl-serine
PUTRESCINE	putrescine

PYR	pyruvate
PYRAPCP	Undecylprodigiosin
QUINOLINATE	quinolinate
R1P	ribose-1-phosphate
R4PPTCYC	R-4'-phosphopantothenoyl-L-cysteine
R5P	D-ribose-5-phosphate
RAF	Raffinose
RH3BUT	(R)-3-Hydroxybutanoate
RH3BUTCOA	(R)-3-Hydroxybutanoyl-CoA
RHAMN	L-Rhamnulose
RHAMN1P	L-Rhamnulose 1-phosphate
RIB	D-ribose
RIBOFLAVIN	riboflavin
RIBUL1P	D-ribulose-5-phosphate
RMN	L-Rhamnose
RNA	RNA (biomass component)
RNICOT	N-Ribosylnicotinamide
RTHIO	Reduced thioredoxin
S	sulfur donor
S17BP	D-Sedoheptulose1,7-bisphosphate
S7P	D-sedoheptulose-7-phosphate
SA4M2OXBUTANOATE	S-adenosyl-4-methylthio-2-oxobutanoate
SAH	S-adenosyl-homocysteine
SAICAIR	5'-phosphoribosyl-4-(N-succinocarboxamide)-5-aminoimidazole
SAL6P	Salicin 6-phosphate
SALICYLATE	salicylate
SAMET	S-adenosyl-L-methionine
SAMETA	S-Adenosylmethioninamine
SAOPIM	N-succinyl-2-amino-6-ketopimelate
SAP	S-Aminomethyldihydrolipoylprotein
SDAPIM	N-succinyl-L,L-2,6-diaminopimelate
SDLIPO	S-succinyl-dihydrolipoamide
SER	L-serine
SER3P	3-phospho-serine
SHCHC	2-succinyl-6-hydroxy-2,4-cyclohexadiene-1-carboxylate
SLF	Sulfate
SMALLMOLECULES	SMALLMOLECULES (biomass component)
SME	shikimate
SME3P	shikimate-3-phosphate
SMMCOA	(S)-Methylmalonyl-CoA
SOT	D-Sorbitol
SPERMIDINE	spermidine
SPERMINE	Spermine

STACHYOSE	Stachyose
STARCHn	Starch
SUCC	succinate
SUCCALD	succinate semialdehyde
SUCCOA	succinyl-CoA
SUCROSE	sucrose
SUPEROXIDE	superoxide
T3	glyceraldehyde
T3METGLUCOA	trans-3-methylglutaconyl-CoA
TAG	Triacylglycerols (biomass component)
TARTALD	tartronate semialdehyde
TARTRATE	tartrate
TDP	dTDP
TDPDGLUCOSE	dTDP-glucose
TDPGAL	dTDP-galactose
TEICH	Teichoic acid (biomass component)
TETRADECANOATE	TETRADECANOATE
THCISOCAPROATE	2-D-threo-hydroxy-3-carboxy-isocaproate
THF	tetrahydrofolate
THFGLU	THF-L-glutamate
THIAMINE	thiamine
THP	thiamine-phosphate
THPP	thiamin diphosphate
THR	L-threonine
THTGLU	tetrahydropteroyltri-L-glutamate
THY	thymine
THZP	4-methyl-5-(β-hydroxyethyl)thiazole phosphate
TIGCOA	tiglyl-CoA
TMP	dTMP
TOLUENE	toluene
TOLUENECISDHDIOL	toluene-cis-1,2-dihydrodiol
TRE	trehalose
TRE6P	alpha,alpha'-Trehalose 6-phosphate
trnaGLT	tRNA(GlT)
TRP	L-tryptophan
TYR	L-tyrosine
UBIQOL	ubiquinol-8
UBIQON	ubiquinone-8
UDP	UDP
UDPAAGLU	UDP-N-acetylmuramoyl-L-alanyl-D-glutamate
UDPAAGMDHDIOATE	UDP-N-acetylmuramoyl-L-alanyl-D-glutamyl-meso-2,6-diaminoheptanedioate
UDPACMURALA	UDP-N-acetylmuramoyl-L-alanine
UDPGAL	UDP-galactose

UDPGLU	UDP-D-glucose
UDPGLUCUR	UDP-D-glucuronate
UDPNACVG	UDP-GlcNAc-pyruvate enol ether
UDPNAG	UDP-N-acetyl-D-glucosamine
UDPNAM	UDP-N-acetylmuramate
UGC	(S)-ureidoglycolate
UMP	UMP
URA	uracil
URATE	urate
UREA	urea
URI	uridine
UROCANATE	Urocanate
UROPORPHYRINOGEN3	UROPORPHYRINOGEN3
UTP	UTP
VAL	L-valine
VANILLATE	Vanillate
XAN	xanthine
XMP	xanthosine-5-phosphate
XNSN	xanthosine
XYL	D-xylose
XYL5P	D-xylulose-5-phosphate
XYLITOL	Xylitol
