# Supplementary material for: Genome-scale metabolic network guided engineering of Streptomyces tsukubaensis for FK506 production improvement
Source: Microb Cell Fact. 2013 May 24;12:52. doi: 10.1186/1475-2859-12-52 (PMC3680238; doi:10.1186/1475-2859-12-52)
Supplement: Additional file 3: Table S1 — The specific FK506 production rate of various engineered strains. [file 1475-2859-12-52-S3.pdf]

Additional file 5

Table S3 The specific FK506 production rate of various engineered strains.

**Strains**	**Specific FK506 production rate (μmol/g DCW/h)**
D852	1.61±0.10
HT-ΔGDH	1.88±0.05
HT-ΔPPC	1.92±0.08
HT-DAHP	2.56±0.09
HT-PNT	2.34±0.11
HT-ACC	2.05±0.08
HT-ZWF	2.11±0.10
HT-ΔGDH-DAHP	2.54±0.05
HT-ΔGDH-PNT	2.60±0.06
HT-ΔGDH-ACC	2.06±0.04
HT-ΔGDH-ZWF	2.03±0.06
HT-ΔPPC-DAHP	2.58±0.09
HT-ΔPPC-PNT	2.39±0.10
HT-ΔPPC-ACC	2.01±0.07
HT-ΔGP	1.54±0.09
HT-ΔGP-DAHP	1.57±0.12
HT-ΔGP-PNT	1.58±0.06
HT-ΔGP-ACC	1.56±0.07
HT-ΔGP-ZWF	1.56±0.06

Results are represented as mean±SD of three independent observations.
