# Supplementary material for: Genome-scale metabolic network guided engineering of Streptomyces tsukubaensis for FK506 production improvement
Source: Microb Cell Fact. 2013 May 24;12:52. doi: 10.1186/1475-2859-12-52 (PMC3680238; doi:10.1186/1475-2859-12-52)

Additional file 1

Figure S1. The effect of glutamate or glutamine addition on cell growth for HT-ΔGDH strain.

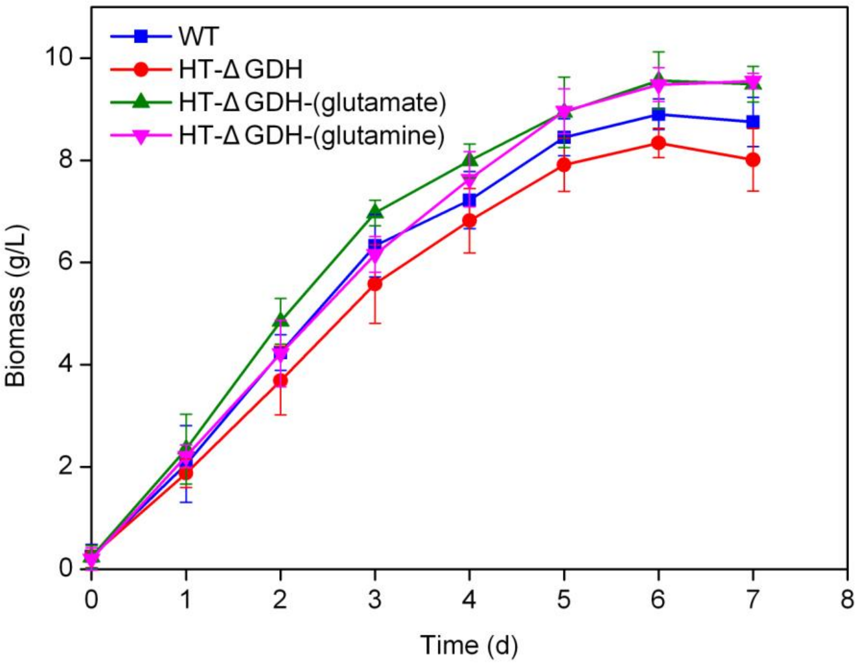

Figure S2. The effect of succinate, fumarate or malate addition on cell growth for HT-ΔPPC strain.

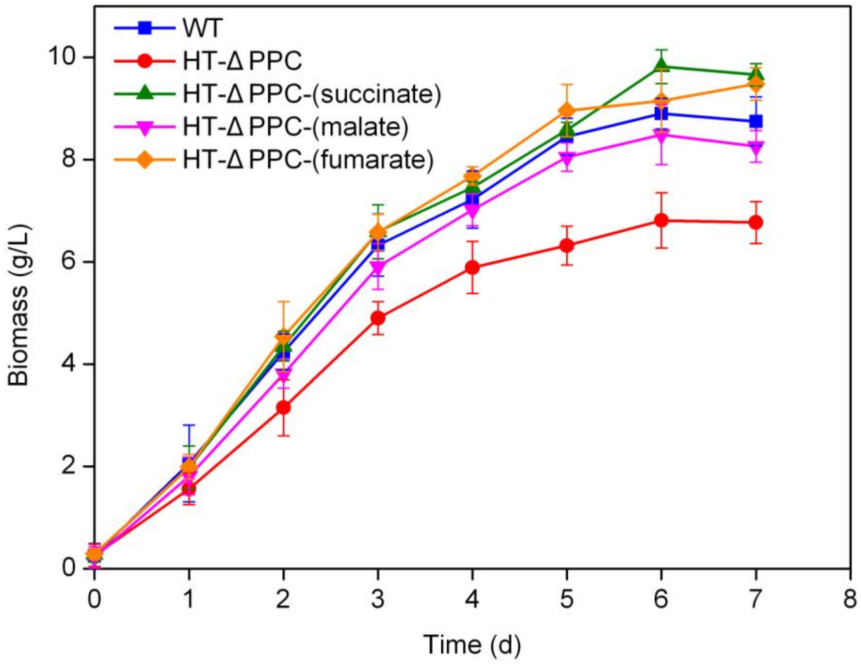

Supplement: Additional file 4: Figure S1 — The effect of glutamate or glutamine addition on cell growth for HT-ΔGDH strain. Figure S2. The effect of succinate, fumarate or malate addition on cell growth for HT-ΔPPC strain. [file 1475-2859-12-52-S4.pdf]
