# Supplementary material for: Genome-scale metabolic network guided engineering of Streptomyces tsukubaensis for FK506 production improvement
Source: Microb Cell Fact. 2013 May 24;12:52. doi: 10.1186/1475-2859-12-52 (PMC3680238; doi:10.1186/1475-2859-12-52)
Supplement: Additional file 5: Table S2 — Strains and plasmids used in this study. Table S3. Primers used in this work. [file 1475-2859-12-52-S5.pdf]

**Additional file 4**

**Table S1** Strains and plasmids used in this study

| **Strains/Plasmids** | **Relevant features**[a] | **Source/reference**[b] |
|---|---|---|
| **Strains** | | |
| *Streptomyces tsukubaensis* | | |
| D852 | Original FK506 producing strain | Laboratory stock CGMCC 7180 |
| HT-PIB139 | D852 transformed with pIB139 | This study |
| HT-DAHP | D852 transformed with pDAHP | This study |
| HT-PNT | D852 transformed with pPNT | This study |
| HT-ACC | D852 transformed with pACC | This study |
| HT-ZWF | D852 transformed with pZWF | This study |
| HT-DP | D852 transformed with pDP | This study |
| HT-DA | D852 transformed with pDA | This study |
| HT-DZ | D852 transformed with pDZ | This study |
| HT-PA | D852 transformed with pPA | This study |
| HT-PZ | D852 transformed with pPZ | This study |
| HT-AZ | D852 transformed with pAZ | This study |
| HT-DPA | D852 transformed with pDPA | This study |
| HT-DPZ | D852 transformed with pDPZ | This study |
| HT-DAZ | D852 transformed with pDAZ | This study |
| HT-PAZ | D852 transformed with pPAZ | This study |
| HT-DPAZ | D852 transformed with pDPAZ | This study |
| HT-ΔGDH | D852 with an in-frame deletion of *gdhA* | This study |
| HT-ΔPPC | D852 with an in-frame deletion of *ppc* | This study |
| HT-ΔGDH-DAHP | HT-ΔGDH transformed with pDAHP | This study |
| HT-ΔGDH-PNT | HT-ΔGDH transformed with pPNT | This study |
| HT-ΔGDH-ACC | HT-ΔGDH transformed with pACC | This study |

| | | |
|---|---|---|
| HT-ΔGDH-ZWF | HT-ΔGDH transformed with pZWF | This study |
| HT-ΔGDH-DP | HT-ΔGDH transformed with pDP | This study |
| HT-ΔGDH-DA | HT-ΔGDH transformed with pDA | This study |
| HT-ΔGDH-DZ | HT-ΔGDH transformed with pDZ | This study |
| HT-ΔGDH-PA | HT-ΔGDH transformed with pPA | This study |
| HT-ΔGDH-PZ | HT-ΔGDH transformed with pPZ | This study |
| HT-ΔGDH-AZ | HT-ΔGDH transformed with pAZ | This study |
| HT-ΔGDH-DPA | HT-ΔGDH transformed with pDPA | This study |
| HT-ΔGDH-DPZ | HT-ΔGDH transformed with pDPZ | This study |
| HT-ΔGDH-DAZ | HT-ΔGDH transformed with pDAZ | This study |
| HT-ΔGDH-PAZ | HT-ΔGDH transformed with pPAZ | This study |
| HT-ΔGDH-DPAZ | HT-ΔGDH transformed with pDPAZ | This study |
| HT-ΔPPC-DAHP | HT-ΔPPC transformed with pDAHP | This study |
| HT-ΔPPC-PNT | HT-ΔPPC transformed with pPNT | This study |
| HT-ΔPPC-ACC | HT-ΔPPC transformed with pACC | This study |
| HT-ΔPPC-ZWF | HT-ΔPPC transformed with pZWF | This study |
| HT-ΔPPC-DP | HT-ΔPPC transformed with pDP | This study |
| HT-ΔPPC-DA | HT-ΔPPC transformed with pDA | This study |
| HT-ΔPPC-DZ | HT-ΔPPC transformed with pDZ | This study |
| HT-ΔPPC-PA | HT-ΔPPC transformed with pPA | This study |
| HT-ΔPPC-PZ | HT-ΔPPC transformed with pPZ | This study |
| HT-ΔPPC-AZ | HT-ΔPPC transformed with pAZ | This study |
| HT-ΔPPC-DPA | HT-ΔPPC transformed with pDPA | This study |
| HT-ΔPPC-DPZ | HT-ΔPPC transformed with pDPZ | This study |
| HT-ΔPPC-DAZ | HT-ΔPPC transformed with pDAZ | This study |
| HT-ΔPPC-PAZ | HT-ΔPPC transformed with pPAZ | This study |
| HT-ΔPPC-DPAZ | HT-ΔPPC transformed with pDPAZ | This study |
| HT-ΔGP | HT-ΔGDH with an in-frame deletion of *ppc* | This study |
| HT-ΔGP-DAHP | HT-ΔGP transformed with pDAHP | This study |
| HT-ΔGP-PNT | HT-ΔGP transformed with pPNT | This study |

| | | |
|---|---|---|
| HT-ΔGP-ACC | HT-ΔGP transformed with pACC | This study |
| HT-ΔGP-ZWF | HT-ΔGP transformed with pZWF | This study |
| HT-ΔGP-DP | HT-ΔGP transformed with pDP | This study |
| HT-ΔGP-DA | HT-ΔGP transformed with pDA | This study |
| HT-ΔGP-DZ | HT-ΔGP transformed with pDZ | This study |
| HT-ΔGP-PA | HT-ΔGP transformed with pPA | This study |
| HT-ΔGP-PZ | HT-ΔGP transformed with pPZ | This study |
| HT-ΔGP-AZ | HT-ΔGP transformed with pAZ | This study |
| *Streptomyces coelicolor* | | |
| A3(2) | Original strain | ATCC |
| *Streptomyces roseosporus* | | |
| NRRL11379 | Original strain | ATCC |
| *Escherichia coli* | | |
| JM109 | Plasmid construction and general subcloning | TransGen Biotech (Beijing, China) |
| ET12567/pUZ8002 | Non-methylating ET12567 containing non-transmissible RP4 derivative plasmid pUZ8002, $Cm^R$, $Kan^R$ | [1] |
| **Plasmids** | | |
| pUC18 | *E. coli* cloning vector; $Amp^R$ | Laboratory stock |
| pIB139 | Intergrative plasmid containing *oriT*, *attP*, *int*, *aac(3)IV* and *ermEp** | [2] |
| pUC119-$Kan^R$ | pUC119 with $Kan^R$ | [3] |
| pKC1139 | Temperature-sensitive *E. coli-Streptomyces* shuttle vector containing *oriT*, *aac(3)IV* for gene disruption | [4] |
| pGH112 | *E. coli-Streptomyces* shuttle vector containing *oriT*, *bla* and *tsr* for gene disruption | [5] |
| pDAHP | pIB139 based integrative plasmid containing *dahp*, $Apr^R$ | This study |
| pPNT | pIB139 based integrative plasmid containing *pntAB*, $Apr^R$ | This study |
| pACC | pIB139 based integrative plasmid containing *accA2*, $Apr^R$ | This study |
| pDP | pIB139 based integrative plasmid containing *dahp* and *pntAB*, $Apr^R$ | This study |
| pDA | pIB139 based integrative plasmid containing *dahp* and *accA2*, $Apr^R$ | This study |
| pDZ | pIB139 based integrative plasmid containing *dahp* and *zwf2*, $Apr^R$ | This study |

| | | |
|---|---|---|
| pPA | pIB139 based integrative plasmid containing *pntAB* and *accA2*, $Apr^R$ | This study |
| pPZ | pIB139 based integrative plasmid containing *pntAB* and *zwf2*, $Apr^R$ | This study |
| pAZ | pIB139 based integrative plasmid containing *accA2* and *zwf2*, $Apr^R$ | This study |
| pDPA | pIB139 based integrative plasmid containing *dahp*, *pntAB* and *accA2*, $Apr^R$ | This study |
| pDPZ | pIB139 based integrative plasmid containing *dahp*, *pntAB* and *zwf2*, $Apr^R$ | This study |
| pDAZ | pIB139 based integrative plasmid containing *dahp*, *accA2* and *zwf2*, $Apr^R$ | This study |
| pPAZ | pIB139 based integrative plasmid containing *pntAB*, *accA2* and *zwf2*, $Apr^R$ | This study |
| pDPAZ | pIB139 based integrative plasmid containing *dahp*, *pntAB*, *accA2* and *zwf2*, $Apr^R$ | This study |
| pΔGDH | pKC1139 based deletion plasmid with in-frame deletion of 500-bp internal to *gdhA*, $Apr^R$, $Kan^R$ | This study |
| pΔPPC | pKC1139 based deletion plasmid with in-frame deletion of 1000-bp internal to *ppc*, $Apr^R$, $Thio^R$ | This study |
| pGDH | pIB139 based integrative plasmid containing *gdhA*, $Apr^R$ | This study |
| pPPC | pIB139 based integrative plasmid containing *ppc*, $Apr^R$ | This study |

[a] $Cm^R$, chloramphenicol resistance; $Kan^R$, kanamycin resistance; $Apr^R$, aparamycin resistance; $Amp^R$, ampicillin resistance; $Thio^R$, thiostrepton resistance; *attP*, plasmid ΦC31 attachment site; *int*, integrase gene, confer the plasmid to integrate in chromosome; *aac(3)IV*, apramycin resistance gene; *bla*, ampicillin resistance gene; *tsr*, thiostrepton resistance gene; *oriT*, origin of transfer; *ermEp**, promoter region of the erythromycin resistance gene.

[b] CGMCC, China General Microbiological Culture Collection; ATCC, American Type Culture Collection.

**Table S2** Primers used in this work

| Primer name | Sequence[a] 5'→3' |
|---|---|
| *dahp*-F | ATCACATATGTAAGGAGGTAATGTTGTGCACGTGA |
| *dahp*-R | TAATTCTAGATTTAGGATCCGGCGAAGACTTCTACTG |
| *pntAB*-F | ATTGACATATGTAAGAGATCTCCGGTGCGTCCAAGA |
| *pntAB*-R | TTATATCTAGATTTAGGATCCAGGCGTTCGAAACGG |
| *accA2*-F | CGTGGCATATGTAAGAGATCTTAGTCCCTAAACTCAGC |
| *accA2*-R | GTATATCTAGATTTAGGATCCCAGGATGCCACTGCG |
| *zwf2*-F | ATGACATATGTAAGAGATCTCAAGCGCCTCGCTCC |
| *zwf2*-R | TTATTCTAGATTTAGGATCCTTGCTGGCCGTGGTG |
| *gdhA*-LF | ACAATCTAGAAGGTCGCCAAGACCCG |
| *gdhA*-LR | CATAGGATCCCCGTCTTGCCGGAGAG |
| *gdhA*-RF | ATATGGTACCAGTCTCCCGCGATCTG |
| *gdhA*-RR | TCTGAATTCTTCGACCTCCACCAGG |
| *ppc*-LF | AATAAAGCTTCTCAAACACCGCAAG |
| *ppc*-LR | GCATTCTAGATGGAGTTGGAGAGC |
| *ppc*-RF | TTAGGATCCCGGCGAGATCAAGGTG |
| *ppc*-RR | TCTGAATTCCTCCGTCCCCCCATC |
| *gdhA*-F | AATACATATGTAACGCCGCCAAGGACGC |
| *gdhA*-R | TTATTCTAGAGGCCGGGCCAAGGGA |
| *ppc*-F | CATACATATGTAACGACTCCAGAACCCC |
| *ppc*-R | TTATTCTAGACTCCGTCCCCCCATC |
| *tsr*-F | TTATTCTAGATGGCGTAATAGCGAAG |
| *tsr*-R | TTAGGATCCTAGGGTTGCCTTTCT |
| pIB-F | TTGCGCCCGATGCTAGTCG |
| pIB-R | GCACGACAGGTTTCCCGACTG |

[a] Underline stand for the restriction sites

## References


1. Kieser T, Bibb MJ, Buttner MJ, Chater KF, Hopwood DA: **Practical *Streptomyces* Genetics.** *United Kingdom: John Innes Foundation;* 2000.
2. Wilkinson CJ, Hughes-Thomas ZA, Martin CJ, Bohm I, Mironenko T, Deacon M, Wheatcroft M, Wirtz G, Staunton J, Leadlay PF: **Increasing the efficiency of heterologous promoters in actinomycetes.** *J Mol Microbiol Biotechnol* 2002, **4:**417-426.
3. Xiang S-H, Li J, Yin H, Zheng J-T, Yang X, Wang H-B, Luo J-L, Bai H, Yang K-Q: **Application of a double-reporter-guided mutant selection method to improve clavulanic acid production in *Streptomyces clavuligerus*.** *Metab Eng* 2009, **11:**310-318.
4. Bierman M, Logan R, O'Brien K, Seno ET, Nagaraja Rao R, Schoner BE: **Plasmid cloning vectors for the conjugal transfer of DNA from *Escherichia coli* to *Streptomyces* spp.** *Gene* 1992, **116:**43-49.
5. Yin H, Xiang S, Zheng J, Fan K, Yu T, Yang X, Peng Y, Wang H, Feng D, Luo Y, et al: **Induction of holomycin production and complex metabolic changes by the *argR* mutation in *Streptomyces clavuligerus* NP1.** *Appl Environ Microbiol* 2012, **78:**3431-3441.
